# Integrated Approach of Life Cycle Assessment and Experimental Design in the Study of a Model Organic Reaction: New Perspectives in Renewable Vanillin-Derived Chemicals

**DOI:** 10.3390/molecules29092132

**Published:** 2024-05-03

**Authors:** Chiara Ruini, Erika Ferrari, Caterina Durante, Giulia Lanciotti, Paolo Neri, Anna Maria Ferrari, Roberto Rosa

**Affiliations:** 1 Department of Sciences and Methods for Engineering, University of Modena and Reggio Emilia, v. Amendola 2, 42122 Reggio Emilia, Italy; paolo.neri@unimore.it (P.N.); annamaria.ferrari@unimore.it (A.M.F.); roberto.rosa@unimore.it (R.R.); 2 Department of Chemical and Geological Sciences, University of Modena and Reggio Emilia, v. Campi 103, 41125 Modena, Italy; erika.ferrari@unimore.it (E.F.); caterina.durante@unimore.it (C.D.);; 3 Interdepartmental Center En&Tech, University of Modena and Reggio Emilia, Tecnopolo di Reggio Emilia, Piazzale Europa 1, 42123 Reggio Emilia, Italy; 4 Department of Economics, Science, Engineering and Design, University of San Marino Republic, v. Consiglio dei Sessanta 99, 47891 Dogana, San Marino

**Keywords:** organic synthesis, LCA, DoE, ReCiPe2016, vanillin

## Abstract

This work is focused on performing a quantitative assessment of the environmental impacts associated with an organic synthesis reaction, optimized using an experimental design approach. A nucleophilic substitution reaction was selected, employing vanillin as the substrate, a phenolic compound widely used in the food industry and of pharmaceutical interest, considering its antioxidant and antitumoral potential. To carry out the reaction, three different solvents have been chosen, namely acetonitrile (ACN), acetone (Ace), and dimethylformamide (DMF). The syntheses were planned with the aid of a multivariate experimental design to estimate the best reaction conditions, which simultaneously allow a high product yield and a reduced environmental impact as computed by Life Cycle Assessment (LCA) methodology. The experimental results highlighted that the reactions carried out in DMF resulted in higher yields with respect to ACN and Ace; these reactions were also the ones with lower environmental impacts. The multilinear regression models allowed us to identify the optimal experimental conditions able to guarantee the highest reaction yields and lowest environmental impacts for the studied reaction. The identified optimal experimental conditions were also validated by experimentally conducting the reaction in those conditions, which indeed led to the highest yield (i.e., 93%) and the lowest environmental impacts among the performed experiments. This work proposes, for the first time, an integrated approach of DoE and LCA applied to an organic reaction with the aim of considering both conventional metrics, such as reaction yield, and unconventional ones, such as environmental impacts, during its lab-scale optimization.

## 1. Introduction

Vanillin (4-hydroxy-3-methoxybenzaldehyde) is a flavor used worldwide in food manufacturing and a precious fragrance agent in cosmetics [1]. Vanillin can be chemically synthesized starting from eugenol, ferulic acid (from rice), coniferyl alcohol (from spruce tree lignin), and guaiacol. Although these approaches can have economic advantages, chemical synthesis is typically characterized by high environmental impacts. A more sustainable approach is provided by biotechnology-based manufacturing [2] or direct extraction from plants rich in its content in their essential oils, mainly *Vanilla plantifolia* L., *Vanilla tahitensis* L., and *Vanilla pompona* L. [3]. Currently, in view of the circular economy, recycling, and exploitation of natural wastes, direct extraction has received reduced interest in favor of more sustainable bio-production from lignin [4]. Indeed, among all the lignin-derived fine chemicals, vanillin represents the only commercialized mono-aromatic compound obtained on a large scale [5]. Nowadays, more than 20% of the overall production of vanillin starts from lignin compounds [6], representing an alternative option for biomass feedstock exploitation [7]. Vanillin derived from lignin has the advantage of being a renewable compound that does not compete with food sources, thus not leading to food security issues, the latter being recognized as a top priority worldwide [7].

Vanillin stands out for a plethora of properties and applications. Firstly, it is a safe compound with well-established therapeutic activities, such as antitumor [8,9], antimutagenic [10], antidiabetic [11], antioxidant [12], and antimicrobial [13] properties.

From a chemical point of view, vanillin can be classified as a bifunctional aromatic compound since it bears two reactive groups, namely the aldehyde and the phenol groups, that can be used for the synthesis of several second-generation vanillin-derived chemicals. Additionally, these compounds can be exploited as building-block monomers for preparing polymeric materials with tuned properties for tailored applications, including coatings, adhesives, elastomers, and optoelectronic materials [14]. For these reasons, vanillin has the potential to be a renewable building block in the cutting-edge chemical industry. Among the chemical reactions that may involve the phenolic group in polymer synthesis, alkylation is particularly interesting in order to add new reactive groups, such as epoxides and vinyl, or simply to bridge vanillin units by an aliphatic/aromatic linkage. The alkylation of the phenolic oxygen is mostly performed through a nucleophilic substitution reaction carried out under basic conditions in the presence of an alkylating agent with a good leaving group, typically a bromide. In order to enhance the conversion, a large excess of the alkylating agent can be used, as well as a long reaction time and a high temperature [15], the latter few all being conditions that affect both the economic burden and the environmental impact of the overall process. As these aspects are of utmost importance, new approaches exploiting Design of Experiment (DoE) and Life Cycle Assessment (LCA) methodologies can be applied to improve the process, both in terms of high reaction yields and low environmental impacts. Indeed, DoE represents an extremely efficient tool to comprehensively understand the effect of selected factors (e.g., solvent, substrate, reactants/reagents, temperature, time, etc.) on the outcome of a given chemical reaction, typically quantified by the sole yield parameter, without any considerations related to the associated environmental impacts. In this latter regard, LCA represents a prominent methodology to quantify, in a trustworthy manner, the potential environmental impacts associated with the whole life cycle of a given product or process [16], and its use has also been recognized as being of paramount importance in the early design stages of a lab-scale procedure [17]. The as-calculated environmental impacts can therefore be implemented among the responses to be analyzed by DoE.

However, some examples can be found in the literature combining the use of LCA and Experimental Design to better evaluate a process by also accounting for its environmental performances. Their fields of application mainly fall within materials science and nanomaterials [18,19], or within the optimization of industrial processes not directly related to synthetic organic chemistry [20,21]. On the other hand, the present study aims to investigate a model organic synthesis process by concurrently employing DoE and LCA methodologies.

Different approaches can be employed in the design of experiments; however, when the explored factors are characterized by distinct natures (quantitative and qualitative), a conventional experimental design [22] is not able to simultaneously examine them, hence a D-optimal response-surface design emerges as the most appropriate option as it enables the consideration of irregular experimental domains and various types of variables [23].

Concerning the assessment of the environmental impacts, LCA methodology can be used to reliably optimize the environmental performances of chemical reactions by comparing the same synthesis conducted with slightly different variations (e.g., different solvents, different reagent amounts, or different reaction times). This approach can help to highlight the synthetic conditions with lower environmental impacts. 

**Figure 1:**
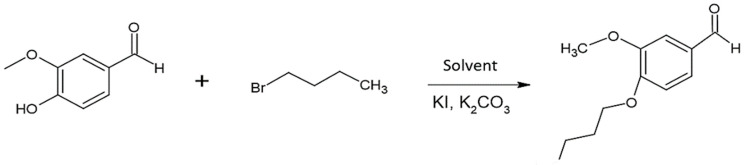
Scheme of the investigated reaction: O-alkylation of vanillin with 1-bromobutane.

In this work, the integrated LCA/DoE approach was applied to a model alkylation reaction (Figure 1) performed on vanillin in order to both improve the conversion and reduce the impacts associated to environmental issues, i.e., wastes, energy consumption, and use of renewable resources. Midpoint (i.e., problem-oriented) results obtained from LCA were exploratorily analyzed by means of Principal Component Analysis (PCA) [24] to gain initial insights and an overarching understanding of the variables under investigation. Subsequently, LCA endpoint (i.e., damage-oriented) results, alongside reaction yield, served as response variables in the Design of Experiments. The obtained dataset was inspected using multilinear regression analysis to obtain the best reaction conditions able to maximize the reaction yield while minimizing the associated environmental impacts.

The aim of this work is therefore to propose, for the first time, an integrated approach of DoE and LCA applied to an organic synthesis. In this way, it should be possible to best optimize a chemical reaction, an organic one in the case of this work, obtaining the optimal reaction conditions to concurrently maximize the reaction yield and minimize the environmental impacts associated with that synthesis by significantly reducing the number of experiments needed.

## 2. Results and Discussion

### 2.1. Synthesis

**Figure 2:**
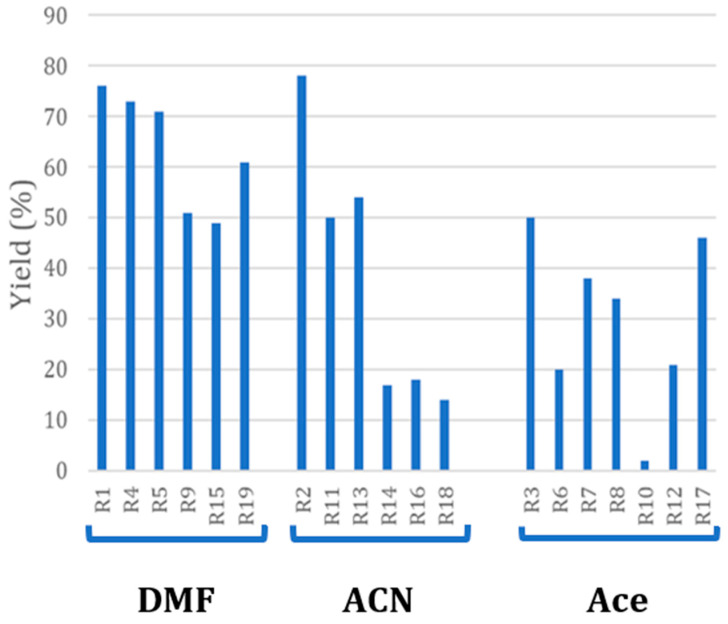
Bar diagram plot representing the yield of pure product (3-methoxy-4butoxy-benzaldehyde) in each experiment. Results are grouped according to the used solvent.

The model reaction selected for the investigation is a nucleophilic substitution performed in a basic environment (K_2_CO_3_) in three different solvents: acetonitrile (ACN), dimethylformamide (DMF), and acetone (Ace). The presence of KI allows the substitution of bromide, providing a better leaving group (iodine). According to the experimental plan, the reaction was performed in different conditions, as reported in Table 1, and the yields of the final product for each run are summarized in Figure 2 and Table S1 in the Supplementary Information.

A total of 26% of all experiments (5/19) provided a yield > 60%, and among them, four out of five runs were carried out in DMF and only one in ACN. None of the synthesis performed in acetone allowed a yield > 60%. A total of 47% of the experiments permitted a yield above 50%. Among them, 59% were carried out in DMF, 33% in ACN, and only 11% in Acetone. All the other experiments (10 out of 19) gave low yields (<50%) and were mostly carried out in Acetone (six out of ten).

Although it is not possible to directly compare the experiments, since they were performed in different conditions according to the experimental plan, it is possible to derive some interesting outcomes. Firstly, all the experiments carried out in DMF resulted in a higher averaged yield with respect to ACN and acetone (67.5% vs. 38.5% and 27.5% for ACN and acetone, respectively). The higher yield levels reached using DMF could be attributed to a higher solubility of reactants and products in the reaction medium as well as the higher temperature at which the reaction was carried out. The higher solubility of carbonate and iodide in ACN rather than in Ace, providing a more basic environment and higher availability of the good leaving group, may account for the greater yields observed in this solvent. Secondly, none of the experiments performed without KI reached a yield greater than 60%, suggesting that the presence of this salt is an important factor in triggering the reaction.

### 2.2. Green Chemistry Metrics

To preliminarily better understand the environmental acceptability of this reaction, some green chemistry metrics were determined. The ones considered are mass intensity (MI), atom economy (AE), atom efficiency (AEf), carbon efficiency (CE), reaction mass efficiency (RME), EcoScale, and environmental factor (E-Factor), calculated according to [25,26,27,28]. The results are reported in Table 2 and their graphical representation is reported in Figures S1–S4 in the Supplementary Information. The results of these green chemistry metrics are coherent with the yields obtained for the nineteen performed reactions. In particular, the MI is higher for reactions with lower yields (such as R10, the reaction with the lowest yield and the highest MI value) and lower for reactions with higher yields (as in the case of R1, R2, and R4). The same trend is also exhibited in the AEf and E-Factor results. For the CE parameter, the results are higher for reactions with higher yields, meaning that there is a much better conversion of reagents into the final product. The values obtained for the EcoScale parameter range from 19% to 57%, even in cases following the yield trend (higher yields for higher EcoScale results). Altogether, these results confirm that reactions with higher yields result in better parameters, i.e., reactions that are greener than the others. However, since the as-calculated metrics are focused on the sole synthesis phase, thus neglecting several contributions related to the production and transport of reagents, solvents, and the electricity mix employed, as well as equipment, emissions, and waste treatments, their environmental performances need to be assessed in a more reliable way by applying a more holistic methodology such as LCA. 

### 2.3. Life Cycle Assessment

**Figure 3:**
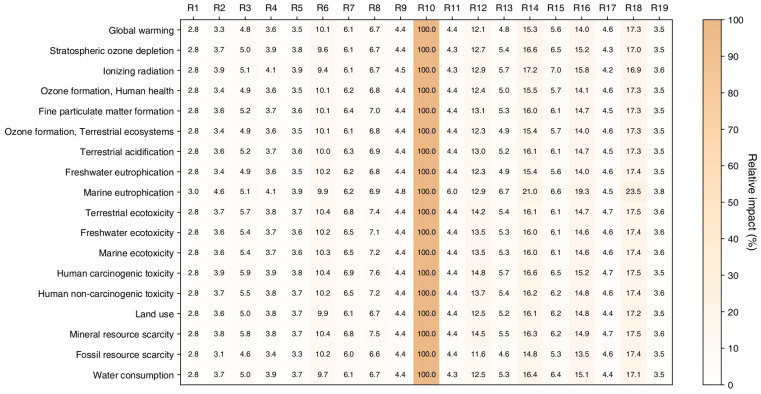
Relative percentage environmental impacts at midpoint level (ReCipe, 2016; H) associated with the production of 1 g of 4-butoxy-3-methoxybenzaldehyde for each of the nineteen reactions, referring to the eighteen impact categories considered.

The potential environmental impacts associated with the organic synthesis of 4-butoxy-3-methoxybenzaldehyde at midpoint level (i.e., problem-oriented) are depicted in Figure 3 as a heat map showing the relative percentage impacts among the nineteen syntheses assessed for each of the eighteen impact categories.

The quantitative results are instead detailed in Table S2 in the Supplementary Information. These results have been calculated for the synthesis of 1 g of product for each of the nineteen reactions. As can be seen, for every impact category considered by the ReCiPe 2016 midpoint method, the reaction with the highest relative impact is reaction R10, conducted in Acetone with a reaction time of 8 h and without using KI as a reagent. This reaction is the one with the lowest yield (2%) and, as a direct consequence of that, it is also the synthesis with the highest environmental impact. Indeed, with respect to the other reactions, the obtainment of 1 g of the final product would require higher amounts of reagents and higher energy consumption, thus necessarily leading to higher environmental impacts. 

By grouping the results of the eighteen impact categories into the opportune damage categories (i.e., Human health, Ecosystems, and Resources) and referring to them at the point at which the environmental effects potentially occur, the endpoint results are obtained. The endpoint Single Score results (thus obtained after normalization and weighting operations performed by the impact assessment method selected) associated with the preparation of 1 g of 4-butoxy-3-methoxybenzaldehyde for each of the nineteen reactions are reported in Table 3. The environmental impacts are expressed in terms of eco-indicator points, Pt (the smaller its value is, the lower the potential environmental impact of that product or process results).

From Table 3, it can immediately be seen that the most affected damage category is Human Health, independently from the reaction considered, followed by Ecosystems and then Resources. Even in this case, as for the midpoint results, it is reaction R10 that had the highest environmental impact. Instead, the reactions with the highest yields (i.e., R1, R2, R4, and R5, with a yield > 70%) are the ones with the lowest environmental impact; again, a high yield can be translated into a lower consumption of reagents, together with reduced energy demand, to obtain 1 g of the final product. 

### 2.4. Midpoint PCA Analysis

**Figure 4:**
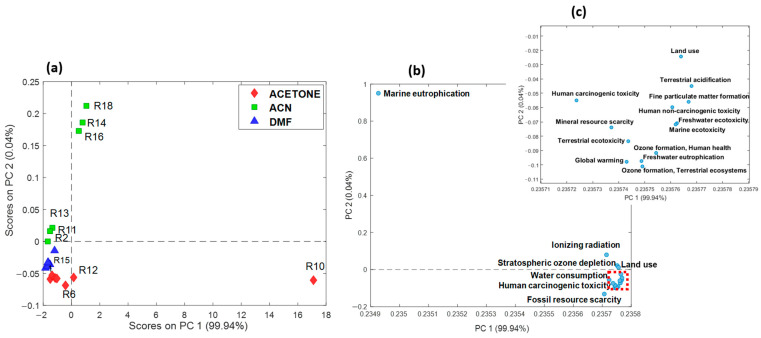
Score (**a**) and loading (**b**) plots of PCA applied on the LCA midpoint dataset. (**c**) Zoom-in of the loadings present within the red dashed area of (**b**).

To comprehensively understand the impact of selected factors, namely the solvent, substrate, reactants/agent, and reaction time, on both the yield of a nucleophilic substitution reaction on vanillin and its environmental implications, a D-optimal response-surface design was employed [23]. The dataset was inspected using multilinear regression analysis to obtain the best reaction conditions able to maximize yield while minimizing environmental impact. LCA midpoint results (Table S2) were subjected to principal component analysis (PCA) to gain insights into the relationships between different experiments and to identify the factors that most influence environmental impact. Data were autoscaled to ensure equal weighting of all midpoint variables and the PCA model was developed using two principal components (PCs), which explained a cumulative variance of 99%. From a synergistic evaluation of the scores and loadings plots, reported in Figure 4a and Figure 4b, respectively, it is possible to note that the first principal component (PC1) clearly distinguished experiment R10 (conducted in acetone) from the other experiments (Figure 4a). 

This experiment, with the highest PC1 scores, presented a higher environmental impact across all midpoint categories (positive PC1 loadings, Figure 4b,c). The second principal component (PC2) distinguished the products of experiments R14, R16, and R18 (conducted in acetonitrile) from the others. These experiments had higher PC2 scores, associated with a higher impact on marine eutrophication (highest positive PC2 score, Figure 4b). A decreasing trend in PC2 scores was observed from products in acetonitrile to those in DMF and finally to those in acetone, with the exception of experiment R10 in acetone. The Marine Eutrophication impact category had higher PC2 loadings for products obtained in acetonitrile, with a decrease in products obtained with acetone (except for experiment R10). Therefore, PCA identified experiment R10 as having the overall highest environmental impact, and marine eutrophication was identified as an impact category particularly sensitive to the type of solvent used.

### 2.5. D-Optimal Design Results

**Figure 5:**
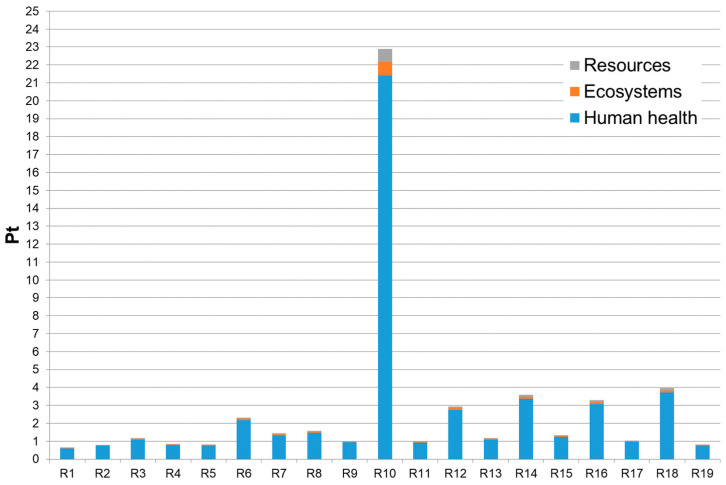
Endpoint single score results (ReCiPe, 2016; H, A) for the synthesis of 1 g of 4-butoxy-3-methoxybenzaldehyde for each of the nineteen reactions (R1–R19).

In Figure 5, the LCA Single Score endpoint results obtained for each reaction conducted according to the DoE experimental plan were reported. In the previous Table 3, the yields and Single Score endpoint results are reported together. 

In order to get a deep overview of the influence of all the investigated factors, four different regression models were developed separately for the four responses: yield (y1), human health (y2), ecosystems (y3), and resources (y4). The explained variance for each model and the significant coefficients are reported in Table 4.

Regarding the yield, a positive significant coefficient indicates that higher values of the corresponding factor led to higher yields. Conversely, a negative coefficient indicates that the lowest value of the factor results in the maximum yield. This is the opposite of the other LCA impact data responses, as their optimal values should be as low as possible.

**Figure 6:**
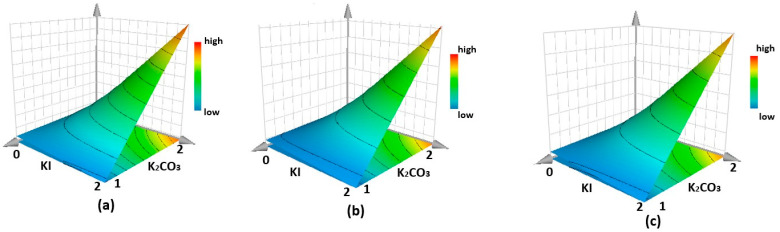
Surface plots showing the effects of KI and K_2_CO_3_ on human health (**a**), ecosystems (**b**), and resources (**c**) responses.

For yield, both the DMF and KI terms are positive and significant, suggesting that the highest yield is achieved when using DMF as a solvent and a high KI content. The other three LCA responses (human health, ecosystems, and resources) exhibit similar trends. Considering the significant coefficients, time, Br, and KI, K_2_CO_3_ content and the interaction between KI and K_2_CO_3_ play key roles in predicting LCA responses. Although the solvent exerts a profound impact on the yield of the reactions, from the MLR results, it appears to be insignificant based on LCA data compared to the other terms. To achieve the lowest possible LCA data values, it seems crucial to use longer reaction times and higher Br quantities. Determining the optimal values for KI and K_2_CO_3_ requires considering their interaction term, as significant interactions impact the optimum. In multivariate models with significant interactions, surface plots are crucial for understanding the effect of factors. The optimum may depend on the interaction between factors, not just on their individual effects. Therefore, surface plots illustrating the effects of KI and K_2_CO_3_ on the three LCA data responses are presented in Figure 6a–c. Across all cases, the best compromise to achieve optimal results (lowest values) seems to be with the highest value for KI and the lowest value for K_2_CO_3_.

Therefore, in this preliminary phase of the study, it is hypothesized that to obtain the optimal results in terms of highest yield and lowest LCA data, the following conditions should be applied: DMF for the conduction reaction, a high molar ratio of potassium iodide to vanillin, a high molar ratio of Br to vanillin, a long reaction time, and a low molar ratio of K_2_CO_3_ to vanillin. Considering these optimal conditions, the theoretical yield predicted using the MLR model is 95.8% ± 8.7%. In order to validate the MLR model, an additional run was carried out following the conditions reported in Table 5. As reported in Table 5, the experimentally obtained yield for the additional synthesis performed was 93%, which is in the predicted variability range.

Using these optimal conditions and considering the experimental yield of 93%, the environmental impacts, at endpoint level, were calculated and reported in Table 5. 

With respect to the endpoint results associated with the nineteen reactions, previously reported in Table 3, the values characterizing the optimal reaction are indeed the lowest ones, independently by the damage category considered (i.e., Human Health, Ecosystems, and Resources). In particular, the experimentally obtained increase in the reaction yield (up to 93%) led to a reduction in the overall potential environmental impacts ranging from 35.5% (with respect to R1) up to 98.2% (with respect to R10).

## 3. Materials and Methods 

All the chemicals and solvents were purchased from Merck (Merck KGaA, Darmstadt, Germany) in the highest purity grade available and used without further purification unless otherwise specified. Nuclear Magnetic Resonance (NMR) spectra were recorded on a Bruker Biospin FT-NMR AVANCE III HD (600 MHz) spectrometer equipped with a CryoProbe BBO H&F 5 mm in inverse detection (Bruker corporation 40 Manning Road Billerica, MA, USA). The nominal frequencies were 150.90 MHz for ^13^C and 600.13 MHz for ^1^H, respectively. 

### 3.1. General Synthesis of 4-Butoxy-3-methoxybenzaldehyde

KI and K_2_CO_3_ were suspended in the selected solvent using a two-neck round-bottom flask (50 mL) and kept under magnetic stirring for 5 min. The mixture was then warmed up at the temperature specified in Table S1 and 1-bromobutane was added, followed by vanillin (van). The reaction was maintained at the same temperature under continuous stirring for the planned reaction time. The final reaction mixture was filtered off to get rid of the salts, and the solvent was removed under reduced pressure by a rotary evaporator. The crude was a light-yellow sticky oil. Yield was estimated by weighing the product and performing NMR analysis using CDCl_3_ as a solvent (20 mg of crude were used). A representative example of the ^1^H NMR spectrum of the product is shown in Figure S5 in the Supplementary Information.

All the specific reaction details for each run are reported in the Experimental plan in Table 1 and Table S1.

### 3.2. Experimental Design Procedure

The optimization of the studied nucleophilic substitution reaction was systematically approached using DoE techniques, aimed at assessing the impact of selected parameters on reaction yield while simultaneously minimizing the environmental footprint. Three solvents (i.e., *N*,*N*-dimethylformamide (DMF), acetone (Ace), and acetonitrile (ACN)) were specifically chosen for conducting the reaction, each associated with distinct reaction temperatures tailored to their respective boiling points.

In addition to solvent variation, the DoE also investigated the following factors: (1) reaction duration (hours), (2) molar ratio of potassium iodide to vanillin, (3) molar ratio of potassium carbonate to vanillin, and (4) molar ratio of bromobutane to vanillin. 

The experimental settings were planned following a D-Optimal Design approach [23], with a center point replicated twice for each solvent. Experiments were randomized and yield and LCA endpoint results were used as responses to be modelled. A linear model was fitted, and the G-efficiency criterion [29] was utilized to determine the optimal experimental layout. This criterion evaluates the performance of D-optimal designs across varying numbers of design runs, with each design being computed based on D-optimality principles, aiming to maximize the determinant of the information matrix related to the candidate set. In particular, G-efficiency is computed as detailed in Equation (1), where *p* is the number of terms in the model, *n* is the number of runs in the design, and *d*_max_ is the maximum relative prediction variance across the candidate set according to Equation (2), where x is a row in the candidate set and X is the selected design.

G-effinciency: 100 × *p*/*n* × *d*_max_ (1)

*d* = x(X′X)^−1^x′ (2)

The factors under investigation, along with their corresponding levels (range of variability), are reported in Table 6.

In summary, a total of 19 experiments, reported in Table 1, were carried out and a multilinear regression model (MLR) was built to develop a predictive model for the chosen response. The applied experimental design allowed the estimation of the coefficients of the mathematical model reported in the following Equation (3), where y represents the response variable (reaction yield or endpoint LCA results refer to human health, ecosystems, or resources areas of protection, AoP), b_i_ are the linear coefficients, and b_ij_ are the coefficients of the interactions. The significance of each term was computed at a significance level of 5%. 

Modde 13.0.2 software was utilized to plan the D-Optimal design and to compute the response surface models.

y = b_0_ + b_1_x_1_ + b_2_x_2_ + b_3_x_3_ + b_4_x_4_ + b_5_x_5_ + b_23_x_2_x_3_ + b_24_x_2_x_4_ + b_25_x_2_x_5_ + b_34_x_3_x_4_ + b_35_x_3_x_5_ + b_45_x_4_x_5_ (3)

### 3.3. Life Cycle Assessment (LCA)

LCA was applied according to the ISO14040-14044 [30,31], as detailed hereafter. 

*Goal and scope definition*

*Goal definition*

The goal of this study was to quantify the potential environmental impacts associated with the organic lab-scale synthesis of 4-butoxy-3-methoxybenzaldehyde, from “cradle to gate”, thus from raw materials extraction up to the isolation of the desired product. 

*System, functional unit, and function of the system*

**Figure 7:**
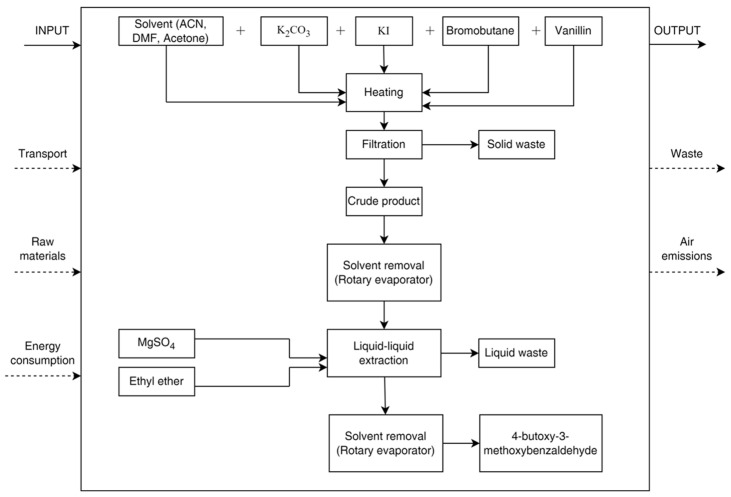
Flowchart summarizing the system boundaries considered in the LCA analysis.

The system object of this study is the chemical synthesis of the viscous oil 4-butoxy-3-methoxybenzaldehyde in order to use its environmental impact data as responses for the experimental design approach. The functional unit selected for this study is 1 g of the product obtained after the synthesis and the workup procedures. The system boundaries consider the chemical synthesis of 4-butoxy-3-methoxybenzaldehyde, followed by the workup and purification phase to obtain the final product. The flowchart summarizing the system boundaries is reported in Figure 7. 

### 3.4. Life Cycle Inventory and Life Cycle Impact Assessment (LCIA)

The data for the LCI were mostly primary data, collected from the experimental activity performed by some of the authors on a laboratory scale. The modelling of the processes was done employing datasets from the Ecoinvent database (EID, version 3.9.1). In particular, an attributional approach was followed, employing the “Allocation at the point of substitution” (i.e., APOS) system model.

The chemicals not present in the Ecoinvent database, such as Vanillin and Bromobutane, were modelled, respectively, according to the synthesis proposed by Taber et al. [32] and Williamson et al. [33]. Regarding transportation, 100 km was considered for the transport contributions. In particular, road freight transport by diesel EURO 6 lorries was assumed with two different lorry capacities of 3.5–7.5 and 16–32 t, respectively, for transportation of reagents and small laboratory equipment and large laboratory equipment. The potential emissions that could have happened during the chemical synthesis were considered, especially the working losses, Lw [34], which were considered and calculated as detailed in Equation (4).

(4) $$L_{W}=\frac{V}{V_{m}}\left(\frac{273.15}{T}\right)\left(\frac{P_{i}^{sat}}{760}\right)\left(MW\right)K_{N}K_{P}$$

The equation is described as follows: V is the volume of the chemical (l), Vm is the molar volume (l) of ideal gas at 0 °C and 1 atm, T is the average temperature (K), Pisat is the vapor pressure of the liquid (mmHg), MW is the molecular weight of the chemical (g/mol), K_N_ is the turnover factor (dimensionless and equal to 1 in the present study), and K_P_ is the working loss product factor (dimensionless), equal to 1 for organic liquids. 

A total of 99% of each emitted substance was considered to be retained by an aspiration system associated with an activated carbon filter. The inventory of the plants and laboratory equipment necessary for the synthesis process is detailed in Tables S3–S18. The inventory for the synthesis of vanillin is detailed in Tables S19–S20. The inventory for the synthesis of Bromobutane is detailed in Tables S21–S23. The complete inventories, for each of the 19 runs that were experimentally performed regarding the chemical synthesis of the product 4-butoxy-3-methoxybenzaldehyde, are detailed in Tables S24–S42. The inventories were modelled in SimaPro 9.5.0.1 [35]. The Life Cycle Impact Assessment (LCIA) phase was conducted by using the global scale-oriented method ReCiPe2016, both at midpoint and endpoint levels, with a hierarchist (H) perspective and average weighting set (A) [36]. This method is one of the most widely accepted and applied global methods, comprising a high number of impact categories [37]. 

## 4. Conclusions

A nucleophilic substitution reaction was performed in a basic environment (K_2_CO_3_) in three different solvents (i.e., *N*,*N*-dimethylformamide (DMF), acetone (Ace), and acetonitrile (ACN)), employing vanillin as a substrate and bromobutane as an alkylating agent. The reaction was conducted in the presence of KI to allow the substitution of bromide, providing a better leaving group (i.e., iodine). Nineteen reactions were experimentally carried out according to the experimental plan obtained by using DoE techniques, aimed at assessing the impact of selected parameters on maximizing reaction yield while simultaneously minimizing environmental footprint. The parameters considered were the type of solvent used, reaction duration (hours), molar ratio of potassium iodide to vanillin, molar ratio of potassium carbonate to vanillin, and molar ratio of bromobutane to vanillin. Based on primary data obtained from the laboratory experiments, an LCA study was conducted on all nineteen reactions. The environmental impacts were obtained, both at midpoint and endpoint levels, and they were used as a further response for a D-optimal design study, together with the experimentally determined yields. Looking at the experimental results, all the reactions carried out in DMF resulted in a higher average yield with respect to ACN and Ace. Moreover, none of the experiments performed without KI reached a yield greater than 60%, suggesting that the presence of this salt is an important factor in triggering the reaction. This was confirmed by analyzing the LCA results. Indeed, both midpoint and endpoint results highlighted that the reaction with the lower yield (i.e., R10) is the one conducted without the KI salt and using acetone as a solvent. This also resulted in the highest environmental impact for R10. The results from the LCA analysis were then used as a response, along with the reaction yield, for a D-optimal study. The results obtained confirmed that the highest yield is achieved when DMF is used as the solvent, employing a high KI content. The other three LCA responses (human health, ecosystems, and resources) exhibit similar trends. Considering these results, some optimal experimental conditions were predicted by the multilinear regression model developed: DMF as a solvent, a high molar ratio of potassium iodide to vanillin, a high molar ratio of Br to vanillin, a long reaction time, and a low molar ratio of K_2_CO_3_ to vanillin. The theoretical yield predicted considering these conditions is 95.8% ± 8.7%. To confirm the predicted optimal conditions, an additional run was carried out following the conditions reported in Table 5. The obtained yield was 93%. 

A validating LCA analysis confirmed that the optimal experimental conditions, together with the experimentally obtained yield of 93%, allow us to obtain the lowest potential environmental impacts, with significant reductions up to −98.2%, as calculated at the endpoint level as a single score. This outcome confirms the validity of the experimental design and its ability to predict optimal conditions by performing a reduced number of experiments.

This work proposes, for the first time, an integrated approach of DoE and LCA applied to a model organic reaction by concurrently considering conventional metrics, such as reaction yield, and metrics that are typically neglected during lab-scale optimization, i.e., associated environmental impacts.

## Figures and Tables

**Table 1:** DoE Experimental Plan (* replicates of the center points).

Experiment	Solvent	Time (h)	KI	K_2_CO_3_	BrBu
R1	DMF	8	2	1	1
R2	ACN	24	2	1	2
R3	Ace	24	2	1	1
R4 *	DMF	16	1	1.5	1.5
R5 *	DMF	16	1	1.5	1.5
R6	Ace	8	0	1	2
R7 *	Ace	16	1	1.5	1.5
R8 *	Ace	16	1	1.5	1.5
R9	DMF	24	0	2	2
R10	Ace	8	0	2	1
R11	ACN	8	2	2	1
R12	Ace	24	2	2	1
R13	ACN	24	0	2	2
R14 *	ACN	16	1	1.5	1.5
R15	DMF	24	0	1	1
R16 *	ACN	16	1	1.5	1.5
R17	Ace	8	2	1	2
R18	ACN	8	0	1	1
R19	DMF	8	2	2	2

**Table 2:** Green Chemistry metrics results for the nineteen performed reactions.

Experiment	Mass Intensity (MI) g/g	Atom Economy (AE) %	Atomy Efficiency (Aef) %	Carbon Efficiency (CE) %	Reaction Mass Efficiency (RME) %	EcoScale %	E-Factor (g/g)
R1	54.90	71.97	54.69	72.90	51.60	56.00	0.94
R2	76.72	71.97	56.13	58.31	37.93	57.00	1.64
R3	117.79	71.97	35.98	50.13	36.08	43.00	1.77
R4	63.24	71.97	52.53	58.04	39.49	54.50	1.53
R5	61.28	71.97	51.10	59.44	40.37	53.50	1.48
R6	284.15	71.97	14.39	15.16	9.85	28.00	9.15
R7	159.95	71.97	27.35	31.16	21.07	37.00	3.75
R8	174.93	71.97	24.47	28.47	19.30	35.00	4.18
R9	86.53	71.97	36.70	36.18	23.55	43.50	3.25
R10	2648.43	71.97	1.44	2.12	1.52	19.00	64.87
R11	123.89	71.97	35.98	48.37	34.81	43.00	1.87
R12	299.04	71.97	15.11	19.41	13.81	28.50	6.24
R13	108.03	71.97	38.86	39.84	25.98	45.00	2.85
R14	366.46	71.97	12.23	13.55	9.24	26.50	9.82
R15	86.79	71.97	35.26	47.60	34.24	42.50	1.92
R16	341.12	71.97	12.95	14.72	9.97	27.00	9.03
R17	130.50	71.97	33.10	33.30	21.60	41.00	3.63
R18	470.05	71.97	10.08	12.10	8.67	25.00	10.53
R19	70.68	71.97	43.90	45.49	29.72	48.50	2.36

**Table 3:** Endpoint Single Score results (ReCiPe, 2016; H) for the synthesis of 1 g or the production of 1 g of 4-butoxy-3-methoxybenzaldehyde for each of the nineteen reactions.

Reaction	Yield (%)	Human Health (Pt)	Ecosystems (Pt)	Resources (Pt)
R1	76	5.99 × 10^−1^	2.11 × 10^−2^	2.01 × 10^−2^
R2	78	7.51 × 10^−1^	2.55 × 10^−2^	2.18 × 10^−2^
R3	50	1.10 × 10^0^	3.69 × 10^−2^	3.24 × 10^−2^
R4	73	7.88 × 10^−1^	2.72 × 10^−2^	2.42 × 10^−2^
R5	71	7.64 × 10^−1^	2.64 × 10^−2^	2.35 × 10^−2^
R6	20	2.17 × 10^0^	7.63 × 10^−2^	7.36 × 10^−2^
R7	38	1.35 × 10^0^	4.62 × 10^−2^	4.24 × 10^−2^
R8	34	1.48 × 10^0^	5.09 × 10^−2^	4.67 × 10^−2^
R9	51	9.46 × 10^−1^	3.33 × 10^−2^	3.16 × 10^−2^
R10	2	2.14 × 10^0^	7.55 × 10^−1^	7.19 × 10^−1^
R11	50	9.38 × 10^−1^	3.30 × 10^−2^	3.16 × 10^−2^
R12	21	2.75 × 10^0^	9.26 × 10^−2^	8.12 × 10^−2^
R13	54	1.09 × 10^0^	3.72 × 10^−2^	3.20 × 10^−2^
R14	17	3.38 × 10^0^	1.17 × 10^−1^	1.05 × 10^−1^
R15	49	1.26 × 10^0^	4.30 × 10^−2^	3.63 × 10^−2^
R16	18	3.08 × 10^0^	1.07 × 10^−1^	9.51 × 10^−2^
R17	46	9.80 × 10^−1^	3.44 × 10^−2^	3.33 × 10^−2^
R18	14	3.71 × 10^0^	1.31 × 10^−1^	1.25 × 10^−1^
R19	61	7.58 × 10^−1^	2.67 × 10^−2^	2.54 × 10^−2^

**Table 4:** MLR regression coefficients.

		y1: Yield (R^2^: 87%)	y2: Human Health (R^2^: 94%)	y3: Ecosystem (R^2^: 94%)	y4: Resources (R^2^: 94%)
b_1_	solvent	DMF	n.s	n.s	n.s
b_2_	time	n.s.	(−)	(−)	(−)
b_3_	Br	n.s.	(−)	(−)	(−)
b_4_	KI	(+)	(−)	(−)	(−)
b_5_	K_2_CO_3_	n.s.	(+)	(+)	(+)
b_4_b_5_	KI*K_2_CO_3_	n.s.	(−)	(−)	(−)

n.s.: not-significant term; (−): lower level; (+): higher level.

**Table 5:** Environmental impact assessment results at Endpoint level (Single Score, ReCiPe 2016; H) obtained considering the optimal conditions predicted by the MLR model, and the yield experimentally determined on the synthesis performed in the optimal conditions predicted.

Reaction	Solvent	Time (h)	Br	KI	K_2_CO_3_	Yield (%)	Human Health (Pt)	Ecosystems (Pt)	Resources (Pt)
Optimal Reaction	DMF	24	2	2	1	93	3.84 × 10^−1^	1.38 × 10^−2^	1.48 × 10^−2^

**Table 6:** Factors investigated by D-Optimal Design.

Factors	Abbreviation	Type	Actual Value
Solvent (x_1_)	Ace	Qualitative	Acetone
	ACN		Acetonitrile
	DMF		*N*,*N*-dimethylformamide
Reaction time (x_2_)	t	Quantitative	8–16–24 h
KI/van molar ratio (x_3_)	KI	Quantitative	0–1–2
K_2_CO_3_/van molar ratio (x_4_)	K_2_CO_3_	Quantitative	1–1.5–2
BrBu/van molar ratio (x_5_)	BrBu	Quantitative	1–1.5–2

## Data Availability

The raw data supporting the conclusions of this article will be made available by the authors on request.

## Supplementary Materials

The following supporting information can be downloaded at: https://www.mdpi.com/article/10.3390/molecules29092132/s1, Figures S1–S4: green chemistry metrics; Figure S5: NMR spectra; Table S1: experimental details; Table S2: LCA midpoint results; Tables S3–S42: LCI inventory tables. References [32,33,38] are cited in the supplementary materials.
