# Supplementary material for: Integrated Approach of Life Cycle Assessment and Experimental Design in the Study of a Model Organic Reaction: New Perspectives in Renewable Vanillin-Derived Chemicals
Source: Molecules. 2024 May 3;29(9):2132. doi: 10.3390/molecules29092132 (PMC11085093; doi:10.3390/molecules29092132)
Supplement: Supplementary file 1 [file molecules-29-02132-s001.zip › molecules-2965215-supplementary.pdf]

SUPPORTING INFORMATION

Integrated approach of Life Cycle Assessment and Experimental Design in the study of an organic synthesis

Chiara Ruini[1*], *Erika Ferrari*[2], *Caterina Durante*[2], *Giulia Lanciotti*[2], *Paolo Neri*[1], *Anna Maria Ferrari*[1,3], *Roberto Rosa*[1,3,4]

[1]Department of Sciences and Methods for Engineering, University of Modena and Reggio Emilia, v. Amendola 2, 42122 Reggio Emilia, Italy

[2]Department of Chemical and Geological Sciences, University of Modena and Reggio Emilia, v. Campi 103, 41125 Modena, Italy

[3]Interdepartmental Center En&Tech, University of Modena and Reggio Emilia, Tecnopolo di Reggio Emilia, Piazzale Europa 1, 42123 Reggio Emilia, Italy

[4]Department of Economics, Science, Engineering and Design, University of San Marino Republic, v. Consiglio dei Sessanta 99, 47891 Dogana, Republic of San Marino

*corresponding author: chiara.ruini@unimore.it

Pages: 83

Figures: 5

Tables: 42

FIGURES

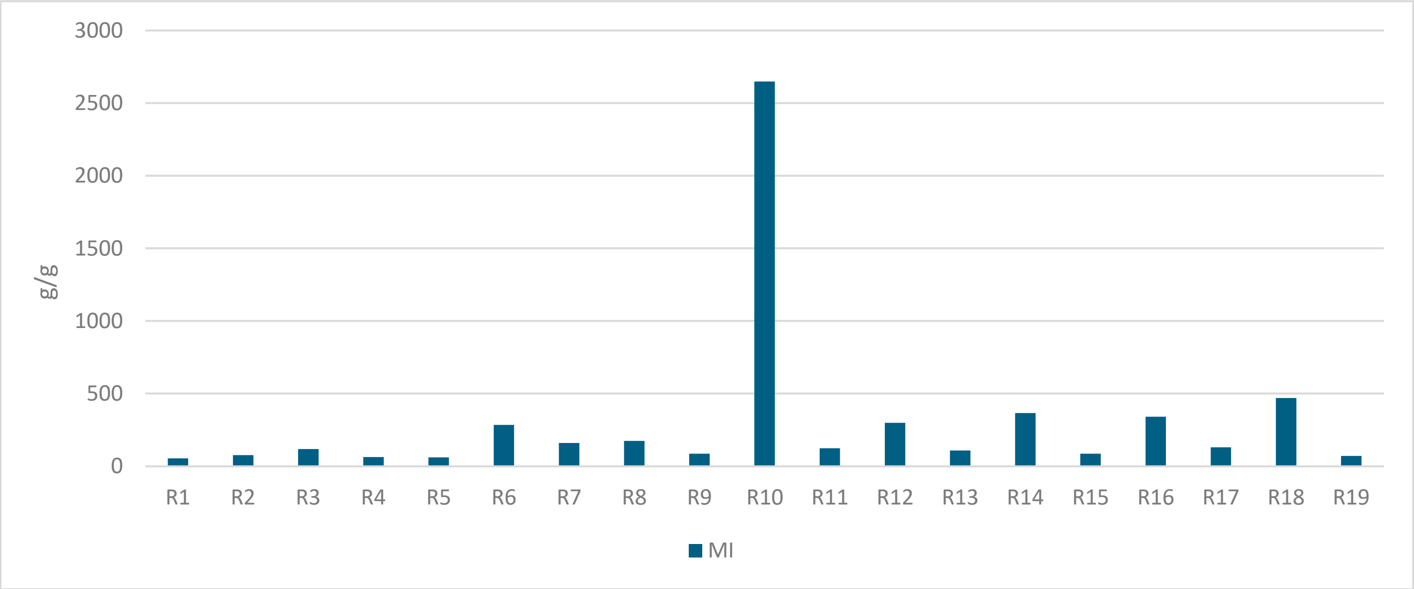

Figure S1. Mass intensity (g/g) results for all the nineteen performed reactions.

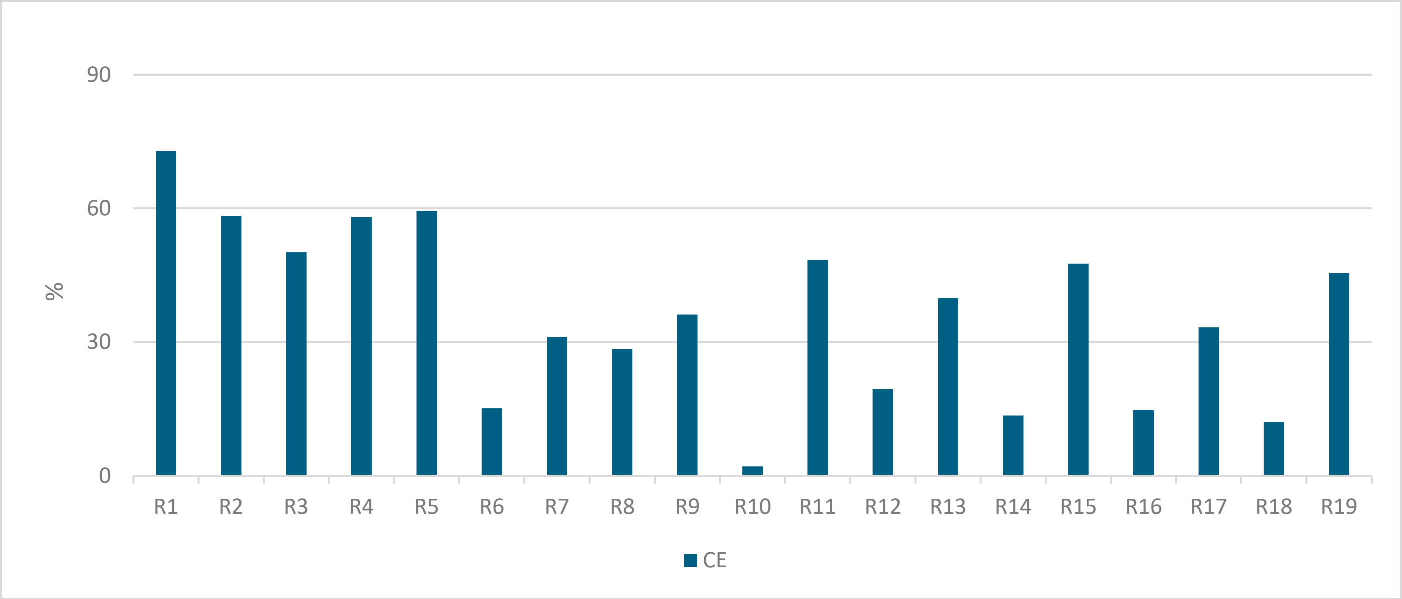

Figure S2. Carbon efficiency (%) results for all the nineteen performed reactions.

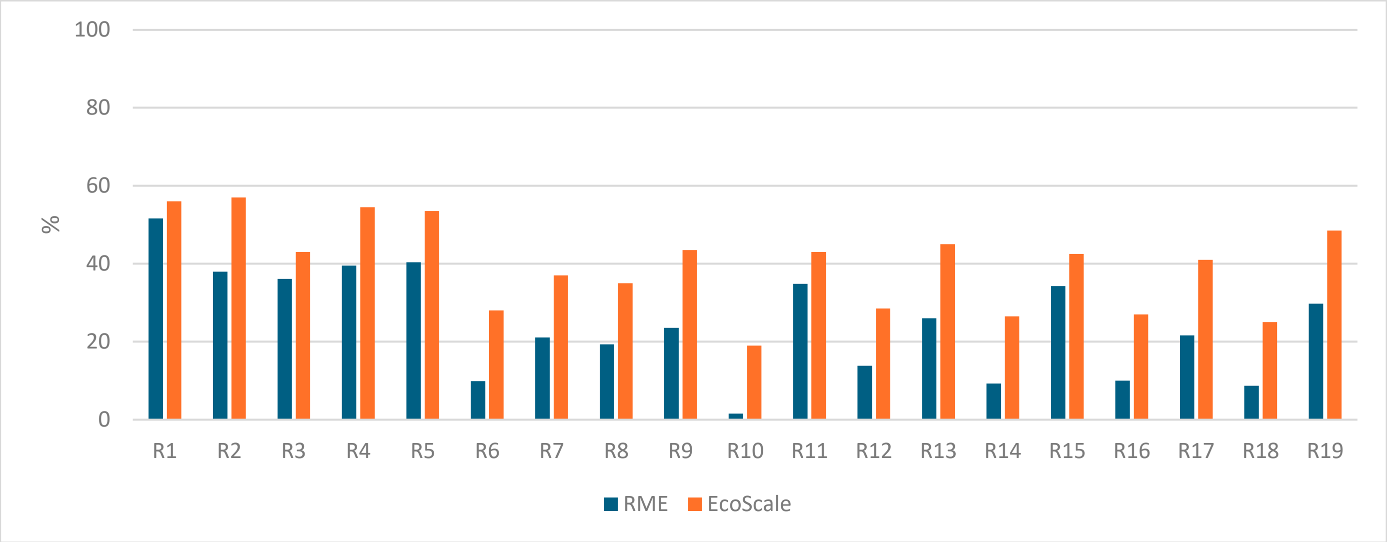

Figure S3. Reaction Mass Efficiency and EcoScale (%) results for all the nineteen performed reactions.

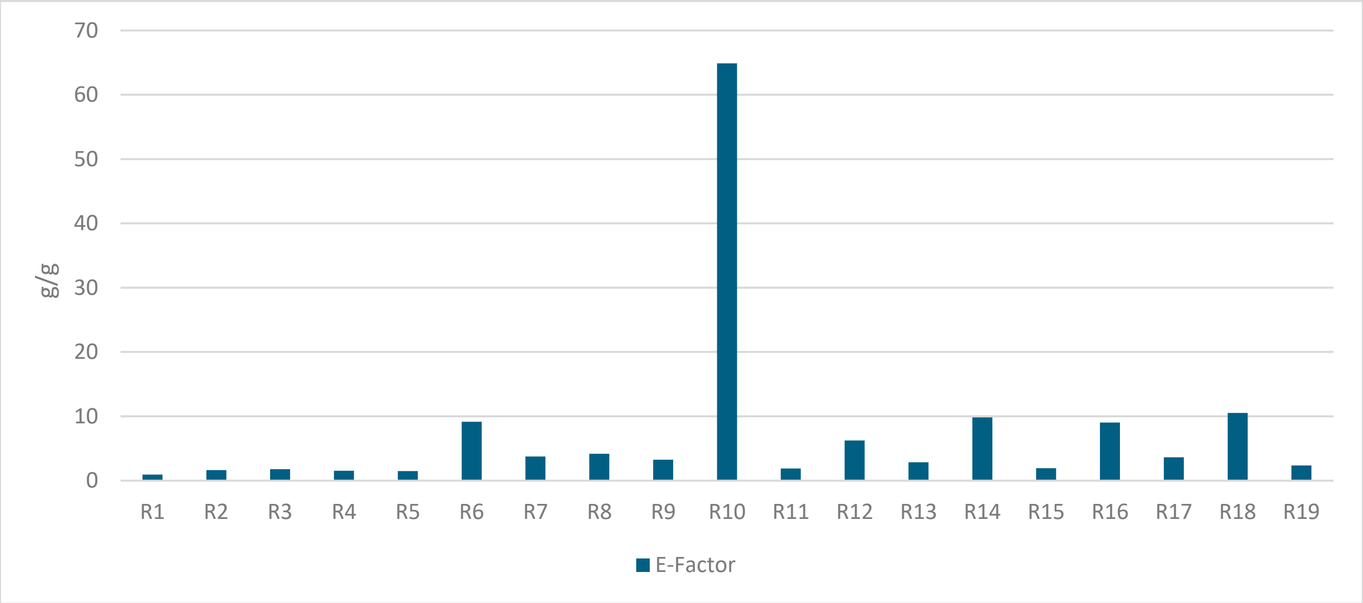

Figure S4. E-factor (g/g) results for all the nineteen performed reactions.

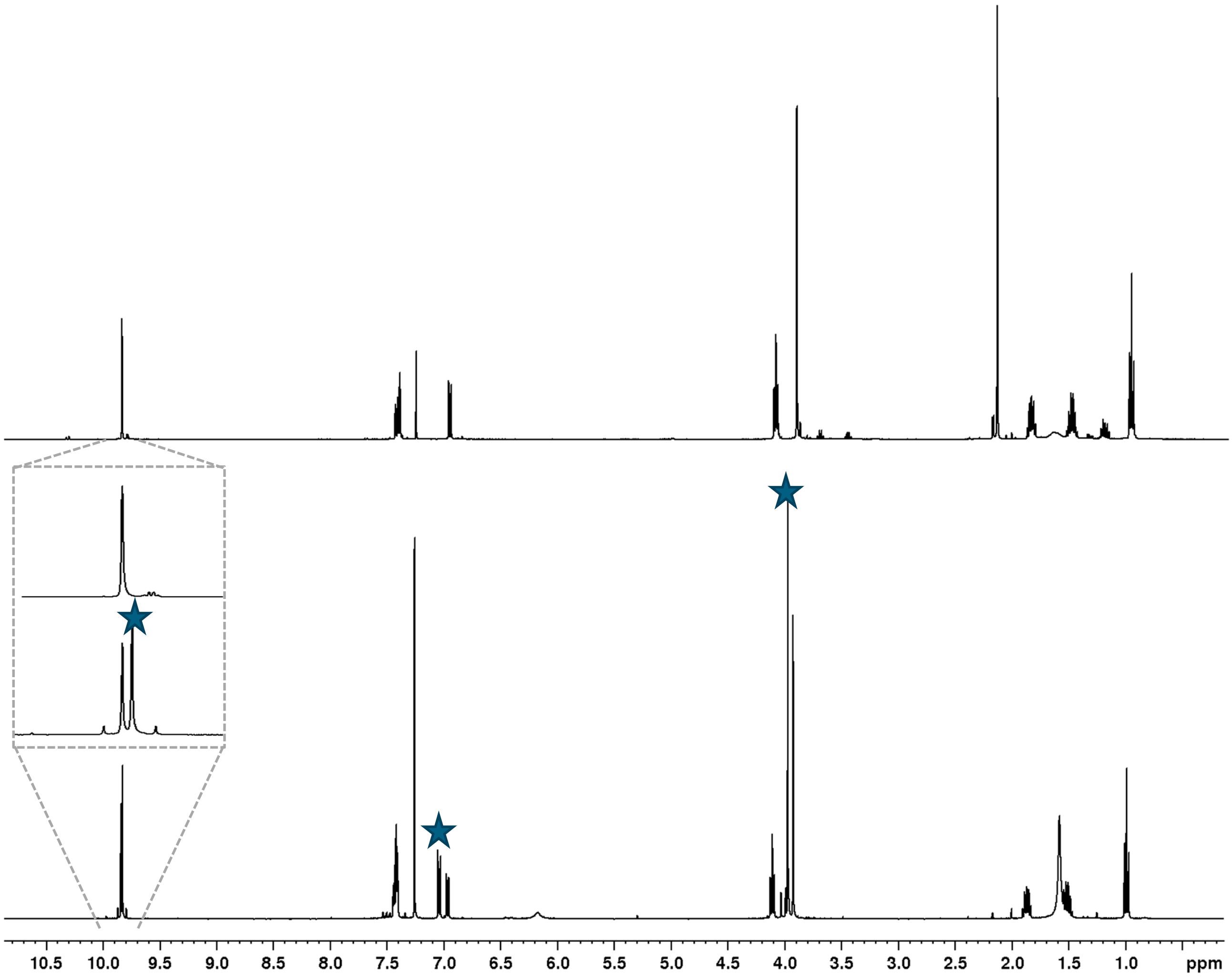

Figure S5. ^{1}H NMR spectra of pure product 4-butoxy-3-methoxybenzaldehyde (top) and reaction crude (bottom). Blue stars highlight typical signals of pure vanillin. (298 K, 400 MHz).

TABLES

Table S1. Experimental details for each synthesis run together with experimental yield of pure 4-butoxy-3-methoxybenzaldehyde (%).

Experiment	Solvent (V/mL)	t (h)	T (°C)	Vanillin (g)	BrBu (g)	KI (g)	K_2CO_3 (g)	Yield (%)
R1	DMF (5mL)	8	100	0.5199	0.5262	1.0221	0.435	76
R2	ACN (20mL)	24	80	0.5010	0.9083	1.0579	0.4710	78
R3	Ace (20 mL)	24	50	0.5027	0.4527	1.0191	0.5185	50
R4	DMF (5mL)	16	100	0.5085	0.6754	0.5594	0.6917	73
R5	DMF (5mL)	16	100	0.5085	0.6851	0.5332	0.6705	71
R6	Ace (20 mL)	8	50	0.5057	0.9220	/	0.4591	20
R7	Ace (20 mL)	16	50	0.5039	0.6991	0.5257	0.7294	38
R8	Ace (20 mL)	16	50	0.5091	0.6932	0.5560	0.7440	34
R9	DMF (5mL)	24	100	0.5162	0.9314	/	0.9201	51
R10	Ace (20 mL)	8	50	0.5186	0.4765	/	0.9117	2
R11	ACN (20 mL)	8	80	0.5004	0.4513	1.0900	0.9200	50
R12	Ace (20 mL)	24	50	0.5045	0.4928	1.1596	0.9074	21
R13	ACN (20 mL)	24	80	0.5181	0.9221	/	0.9136	54
R14	ACN (20 mL)	16	80	0.5197	0.6796	0.6494	0.6765	17
R15	DMF (5mL)	24	100	0.5044	0.4564	/	0.4525	49
R16	ACN (20 mL)	16	80	0.5027	0.6872	0.5431	0.6834	18
R17	Ace (20 mL)	8	50	0.5122	0.9474	1.1229	0.4579	46
R18	ACN (20 mL)	8	80	0.5033	0.4656	/	0.4506	14
R19	DMF (5 mL)	8	100	0.5278	0.9276	1.0654	0.9167	61

Table S2. Life Cycle assessment Midpoint results (ReCiPe 2016; H) for all the nineteen reactions.

Reaction	GWP	SOD	IR	OFHH	FPMF	OFT	TA	FE	ME	TE	FET	MET	HCT	HNCT	LU	MRS	FRS	WC
**R1**	1.84E+01	6.56E-06	8.47E-01	3.12E-02	1.79E-02	3.39E-02	4.89E-02	6.16E-03	3.73E-04	6.93E+01	6.85E-01	9.04E-01	1.23E+00	1.30E+01	4.24E-01	6.25E-02	7.62E+00	2.03E-01
**R2**	2.19E+01	8.71E-06	1.18E+00	3.79E-02	2.31E-02	4.07E-02	6.31E-02	7.38E-03	5.74E-04	9.22E+01	8.86E-01	1.17E+00	1.72E+00	1.71E+01	5.40E-01	8.46E-02	8.56E+00	2.65E-01
**R3**	3.17E+01	1.18E-05	1.55E+00	5.51E-02	3.35E-02	5.93E-02	9.06E-02	1.08E-02	6.36E-04	1.40E+02	1.31E+00	1.74E+00	2.59E+00	2.53E+01	7.53E-01	1.29E-01	1.26E+01	3.61E-01
**R4**	2.35E+01	9.15E-06	1.23E+00	4.04E-02	2.40E-02	4.35E-02	6.55E-02	7.88E-03	5.02E-04	9.30E+01	9.13E-01	1.21E+00	1.70E+00	1.75E+01	5.73E-01	8.47E-02	9.39E+00	2.79E-01
**R5**	2.28E+01	8.87E-06	1.19E+00	3.92E-02	2.33E-02	4.22E-02	6.36E-02	7.65E-03	4.87E-04	9.02E+01	8.86E-01	1.17E+00	1.65E+00	1.70E+01	5.56E-01	8.22E-02	9.11E+00	2.71E-01
**R6**	6.68E+01	2.25E-05	2.83E+00	1.13E-01	6.45E-02	1.23E-01	1.76E-01	2.23E-02	1.23E-03	2.58E+02	2.50E+00	3.30E+00	4.55E+00	4.72E+01	1.49E+00	2.32E-01	2.78E+01	7.01E-01
**R7**	4.01E+01	1.43E-05	1.84E+00	6.87E-02	4.06E-02	7.43E-02	1.10E-01	1.35E-02	7.72E-04	1.67E+02	1.59E+00	2.10E+00	3.02E+00	3.03E+01	9.26E-01	1.52E-01	1.63E+01	4.39E-01
**R8**	4.41E+01	1.57E-05	2.02E+00	7.56E-02	4.47E-02	8.18E-02	1.21E-01	1.49E-02	8.49E-04	1.83E+02	1.74E+00	2.30E+00	3.32E+00	3.33E+01	1.02E+00	1.67E-01	1.79E+01	4.83E-01
**R9**	2.91E+01	1.04E-05	1.34E+00	4.92E-02	2.82E-02	5.34E-02	7.73E-02	9.71E-03	5.90E-04	1.09E+02	1.08E+00	1.43E+00	1.94E+00	2.05E+01	6.71E-01	9.88E-02	1.20E+01	3.21E-01
**R10**	6.60E+02	2.34E-04	3.01E+01	1.12E+00	6.40E-01	1.21E+00	1.75E+00	2.20E-01	1.24E-02	2.47E+03	2.44E+01	3.22E+01	4.39E+01	4.62E+02	1.51E+01	2.23E+00	2.73E+02	7.25E+00
**R11**	2.89E+01	1.01E-05	1.29E+00	4.87E-02	2.79E-02	5.29E-02	7.66E-02	9.64E-03	7.37E-04	1.09E+02	1.08E+00	1.42E+00	1.94E+00	2.03E+01	6.59E-01	9.88E-02	1.20E+01	3.13E-01
**R12**	7.97E+01	2.96E-05	3.88E+00	1.38E-01	8.40E-02	1.49E-01	2.27E-01	2.71E-02	1.60E-03	3.52E+02	3.29E+00	4.36E+00	6.50E+00	6.34E+01	1.89E+00	3.23E-01	3.16E+01	9.05E-01
**R13**	3.19E+01	1.26E-05	1.71E+00	5.53E-02	3.36E-02	5.94E-02	9.18E-02	1.08E-02	8.32E-04	1.34E+02	1.29E+00	1.70E+00	2.49E+00	2.49E+01	7.83E-01	1.23E-01	1.25E+01	3.86E-01
**R14**	1.01E+02	3.88E-05	5.18E+00	1.73E-01	1.03E-01	1.86E-01	2.81E-01	3.38E-02	2.61E-03	3.98E+02	3.90E+00	5.15E+00	7.27E+00	7.48E+01	2.43E+00	3.63E-01	4.05E+01	1.19E+00
**R15**	3.69E+01	1.53E-05	2.10E+00	6.42E-02	3.90E-02	6.87E-02	1.07E-01	1.24E-02	8.18E-04	1.52E+02	1.48E+00	1.96E+00	2.83E+00	2.87E+01	9.36E-01	1.39E-01	1.43E+01	4.64E-01
**R16**	9.21E+01	3.56E-05	4.76E+00	1.58E-01	9.38E-02	1.70E-01	2.57E-01	3.08E-02	2.40E-03	3.64E+02	3.57E+00	4.71E+00	6.66E+00	6.84E+01	2.23E+00	3.32E-01	3.68E+01	1.09E+00
**R17**	3.01E+01	1.01E-05	1.27E+00	5.09E-02	2.91E-02	5.53E-02	7.93E-02	1.01E-02	5.53E-04	1.16E+02	1.13E+00	1.49E+00	2.05E+00	2.13E+01	6.71E-01	1.05E-01	1.25E+01	3.16E-01
**R18**	1.14E+02	3.98E-05	5.09E+00	1.93E-01	1.11E-01	2.09E-01	3.03E-01	3.82E-02	2.91E-03	4.33E+02	4.25E+00	5.62E+00	7.67E+00	8.05E+01	2.60E+00	3.91E-01	4.74E+01	1.24E+00
**R19**	2.33E+01	8.29E-06	1.07E+00	3.95E-02	2.26E-02	4.28E-02	6.19E-02	7.80E-03	4.72E-04	8.77E+01	8.66E-01	1.14E+00	1.56E+00	1.64E+01	5.35E-01	7.91E-02	9.64E+00	2.57E-01

The impact categories considered by the impact assessment method are: Global warming (GW, kg CO2 eq), stratospheric ozone depletion (SOD, kg CFC-11 eq), ionizing radiation (IR, kBq Co-60 eq), ozone formation-human health (OFHH, kg NOx eq), fine particulate matter formation (FPMF, kg PM2.5 eq), ozone formation-terrestrial ecosystems (OFTE, kg NOx eq), terrestrial acidification (TA, kg SO2 eq), freshwater eutrophication (FE, kg P eq), marine eutrophication (ME, kg N eq), terrestrial ecotoxicity (TE, kg 1,4-DCB), freshwater ecotoxicity (FET, kg 1,4-DCB), marine ecotoxicity (MET, kg 1,4-DCB), human carcinogenic toxicity (HCT, kg 1,4-DCB), human non-carcinogenic toxicity (HNCT, kg 1,4-DCB), land use (LU, m2a crop eq), mineral resource scarcity (MRS, kg Cu eq), fossil resource scarcity (FRS, kg oil eq), and water consumption (WC, m^3).

Table S3. Contributions to the Life Cycle Inventory (LCI) for the production of 1p of the aspiration system. The aspiration system, with an air flow rate of 250 m^3/h was considered composed of an electric ventilator, a hood, a chimney and a channel. The lifetime of the electric ventilator was assumed to be 18000 h, while the one of the aspiration system was assumed to be 79750 h.

Description			Amount	Process data source
Input	Materials	Steel	25.24 kg	Ecoinvent v. 3.9[1]
		Copper	8.41 kg	Ecoinvent v. 3.9[2]
		Aluminum	4.21 kg	Ecoinvent v. 3.9[3]
		PVC	4.21 kg	Ecoinvent v. 3.9[4]
		Steel	20.93 kg	Ecoinvent v. 3.9[5]
		Steel	206.09 kg	Ecoinvent v. 3.9[6]
		Steel	115.93 kg	Ecoinvent v. 3.9[7]
	Processing	Forming steel	25.24 kg	Ecoinvent v. 3.9[8]
		Forming copper	8.41 kg	Ecoinvent v. 3.9[9]
		Forming aluminum	4.21 kg	Ecoinvent v. 3.9[10]
		Forming PVC	4.21 kg	Ecoinvent v. 3.9[11]
		Forming steel	342.95 kg	Ecoinvent v. 3.9[12]
	Transport	Transport of raw materials	38.50 tkm	Ecoinvent v. 3.9[13]
Output	End of life	Recycling of steel	25.24 kg	Ecoinvent v. 3.9[14]
		Recycling of copper	8.41 kg	Ecoinvent v. 3.9[15]
		Recycling of Aluminum	4.21 kg	Ecoinvent v. 3.9[16]
		Recycling of plastic	4.21 kg	Ecoinvent v. 3.9[17]
		Recycling of steel	342.95 kg	Ecoinvent v. 3.9[18]

[1]60% of the weight of the electric ventilator was supposed to be steel. The Ecoinvent process used was: Reinforcing steel {GLO}| market for | APOS, U.

[2]20% of the weight of the electric ventilator was supposed to be copper. The Ecoinvent process used was: Copper-rich materials {GLO}| copper, anode to generic market for copper-rich materials | APOS, U.

[3]10% of the weight of the electric ventilator was supposed to be aluminum. The Ecoinvent process used was: Aluminium, primary, ingot {RoW}| market for | APOS, U.

[4]10% of the weight of the electric ventilator was supposed to be polyvinylchloride. The Ecoinvent process used was: Polyvinylchloride, bulk polymerised {GLO}| market for | APOS, U.

[5]Steel of the hood. The Ecoinvent process used was: Reinforcing steel {GLO}| market for | APOS, U.

[6]Steel of the channel connecting the electric ventilator to the hood. The Ecoinvent process used was: Reinforcing steel {GLO}| market for | APOS, U.

[7]Steel of the channel connecting the electric ventilator to the exit. The Ecoinvent process used was: Reinforcing steel {GLO}| market for | APOS, U.

[8]Forming of the steel composing the electric ventilator. The Ecoinvent process used was: Section bar rolling, steel {GLO}| market for | APOS, U.

[9]Forming of the copper composing the electric ventilator. The Ecoinvent process used was: Wire drawing, steel {GLO}| market for | APOS, U.

[10]Forming of the aluminum composing the electric ventilator. The Ecoinvent process used was: Section bar extrusion, aluminium {GLO}| market for | APOS, U.
[11]Forming of the polyvinylchloride composing the electric ventilator. The Ecoinvent process used was: Blow moulding {GLO}| market for | APOS, U.
[12]Forming of the steel channel. The Ecoinvent process used was: Section bar rolling, steel {GLO}| market for | APOS, U.
13The Ecoinvent process used was: Transport, freight, lorry 16-32 metric ton, EURO6 {RoW}| transport, freight, lorry 16-32 metric ton, EURO6 | APOS, U. An average distance of 100 km was considered.
[14]The recycling process for steel was modified by the authors. Particularly a new multi-output process was created with the input of 1 kg of steel (the Ecoinvent process used was Steel, low-alloyed {RER}| steel production, electric, low-alloyed | APOS, U). The main product of the process created was the recycling treatment of 1.105 kg of steel, while the co-product was 1 kg of secondary steel. The environmental loads were allocated to both the recycling process and the secondary recycled steel obtained with a contribution of 50 % each. The process comprised also the end of life of the remaining scraps (the Ecoinvent process used was Scrap steel {CH}| treatment of, inert material landfill | APOS, U).
[15]The recycling process for copper was modified by the authors. Particularly a new process was created with the inputs taken from the Ecoinvent database process named Copper, cathode {RoW}| treatment of metal part of electronic scrap, in copper, anode, by electrolytic refining | APOS, U. However, the environmental loads were allocated to both the recycling process and the secondary recycled copper with a contribution of 50 % each.
[16]The recycling process for aluminum was modified by the authors. Particularly a new process was created with the inputs taken from the Ecoinvent database process named Aluminium, wrought alloy {RER}| treatment of aluminium scrap, new, at remelter | APOS, U. However, the environmental loads were allocated to both the recycling process and the secondary recycled copper with a contribution of 50 % each.
[17]The recycling process for plastic was modified by the authors. Particularly a new process was created with the inputs taken from the Ecoinvent database process of the Polypropylene recycling. However, the environmental loads were allocated to both the recycling process and the secondary recycled plastic with a contribution of 50 % each.
[18]The recycling process for steel was modified by the authors. A new multi-output process was created with the input of 1 kg of steel (the Ecoinvent process used was Steel, low-alloyed {RER}| steel production, electric, low-alloyed | APOS, U). The main product of the process created was the recycling treatment of 1.105 kg of steel, while the co-product was 1 kg of secondary steel. The environmental loads were allocated to both the recycling process and the secondary recycled steel obtained with a contribution of 50 % each. The process comprised also the end of life of the remaining scraps (the Ecoinvent process used was Scrap steel {CH}| treatment of, inert material landfill | APOS, U).

Table S4. Contributions to the Life Cycle Inventory (LCI) for the production of 1p of the activated carbon air. The activated carbon air filter was considered for a chemical hood with an air flow rate of 250 m^3/h, and the velocity of filter crossing of the air was assumed to be 0.5 m/s. The lifetime of the filter was assumed to be 20000 h, while that of activated carbon was assumed to be 2400 h.

Description			Amount	Process data source
Input	Materials	Activated carbon	54.40 kg	Ecoinvent v. 3.9[1]
		Steel	12.68 kg	Ecoinvent v. 3.9[2]
	Processing	Forming steel	12.68 kg	Ecoinvent v. 3.9[3]
	Transport	Transport of raw materials	6.71 tkm	Ecoinvent v. 3.9[4]
Output	End of life	Recycling	12.68 kg	Ecoinvent v. 3.9[5]
		Treatment of spent activated carbon	54.40 kg	Ecoinvent v. 3.9[6]

[1]A single load of activated carbon weights 6.528 kg. The reported amount accounts also for the activated carbon needed during the whole filter lifetime. The Ecoinvent process used was: Activated carbon, granular {GLO}| market for activated carbon, granular | APOS, U.
[2]Weight of the filter container. The Ecoinvent process used was: Reinforcing steel {GLO}| market for | APOS, U.
[3]The Ecoinvent process used was: Sheet rolling, steel {GLO}| market for | APOS, U.
[4]The Ecoinvent process used was: Transport, freight, lorry 16-32 metric ton, EURO6 {RoW}| transport, freight, lorry 16-32 metric ton, EURO6 | APOS, U. An average distance of 100 km was considered.

[5]The recycling process for steel was modified by the authors. Particularly a new multi-output process was created with the input of 1 kg of steel (the Ecoinvent process used was Steel, low-alloyed {RER}| steel production, electric, low-alloyed | APOS, U). The main product of the process created was the recycling treatment of 1.105 kg of steel, while the co-product was 1 kg of secondary steel. The environmental loads were allocated to both the recycling process and the secondary recycled steel obtained with a contribution of 50 % each. The process comprised also the end of life of the remaining scraps (the Ecoinvent process used was Scrap steel {CH}| treatment of, inert material landfill | APOS, U).
[6]The Ecoinvent process used was: Spent activated carbon, granular {GLO}| market for spent activated carbon, granular | APOS, U.

Table S5. Contributions to the Life Cycle Inventory (LCI) for the production of 1p of glass flask. The lifetime of the glass flask was assumed to be 28800 h (considering 15 years of use for 48 week per year, 5 days per week and 8 hours per day).

Description			Amount	Process Data Source
Input	Material	Borosilicate glass	63.49 g	Ecoinvent v 3.8[1]
	Transport	Transport of raw materials	6.35 kgkm	Ecoinvent v 3.8[2]
Output	End of life	Landfill	63.49 g	Ecoinvent v 3.8[3]

[1]The Ecoinvent process used was: Glass tube, borosilicate {RoW}| production | APOS, U.
[2]The Ecoinvent process used was: Transport, freight, lorry 3.5-7.5 metric ton, EURO6 {RER}| transport, freight, lorry 3.5-7.5 metric ton, EURO6 | APOS, U. An average distance of 100km was considered.
[3]The Ecoinvent process used was: Inert waste, for final disposal {RoW}| treatment of inert waste, inert material landfill APOS, U.

Table S6. Contributions to the Life Cycle Inventory (LCI) for the production of 1p of glass silicon oil container. The lifetime of the container was assumed to be 28800 h (considering 15 years of use for 48 week per year, 5 days per week and 8 hours per day).

Description			Amount	Process Data Source
Input	Material	Borosilicate glass	126.76 g	Ecoinvent v 3.8[1]
	Transport	Transport of raw materials	12.676 kgkm	Ecoinvent v 3.8[2]
Output	End of life	Landfill	126.76g	Ecoinvent v 3.8[3]

[1]The Ecoinvent process used was: Glass tube, borosilicate {RoW}| production | APOS, U.
[2]The Ecoinvent process used was: Transport, freight, lorry 3.5-7.5 metric ton, EURO6 {RER}| transport, freight, lorry 3.5-7.5 metric ton, EURO6 | APOS, U. An average distance of 100km was considered.
[3]The Ecoinvent process used was: Inert waste, for final disposal {RoW}| treatment of inert waste, inert material landfill APOS, U.

Table S7. Contributions to the Life Cycle Inventory (LCI) for the production of 1p of paper filter. The lifetime of the filter was assumed to be three years, 26280 hours.

Description			Amount	Process Data Source
Input	Material	Cellulose	0.492 g	Ecoinvent v 3.8[1]
	Transport	Transport of raw materials	$4.92*10^{-2}$ kgkm	Ecoinvent v 3.8[2]
Output	End of life	Landfill	0.492 g	Ecoinvent v 3.8[3]

[1]The Ecoinvent process used was: Cellulose fibre {RoW}| cellulose fibre production | APOS, U.
[2]The Ecoinvent process used was: Transport, freight, lorry 3.5-7.5 metric ton, EURO6 {RER}| transport, freight, lorry 3.5-7.5 metric ton, EURO6 | APOS, U. An average distance of 100 km was considered.
[3]The Ecoinvent process used was: Inert waste, for final disposal {RoW}| treatment of inert waste, inert material landfill | APOS, U.

Table S8. Contributions to the Life Cycle Inventory (LCI) for the production of 1p of glass funnel. The lifetime of the container was assumed to be 28800 h (considering 15 years of use for 48 week per year, 5 days per week and 8 hours per day).

Description			Amount	Process Data Source
Input	Material	Borosilicate glass	12.87 g	Ecoinvent v 3.8[1]
	Transport	Transport of raw materials	1.287 kgkm	Ecoinvent v 3.8[2]
Output	End of life	Landfill	12.87 g	Ecoinvent v 3.8[3]

[1]The Ecoinvent process used was: Glass tube, borosilicate {RoW}| production | APOS, U.
[2]The Ecoinvent process used was: Transport, freight, lorry 3.5-7.5 metric ton, EURO6 {RER}| transport, freight, lorry 3.5-7.5 metric ton, EURO6 | APOS, U. An average distance of 100km was considered.
[3]The Ecoinvent process used was: Inert waste, for final disposal {RoW}| treatment of inert waste, inert material landfill APOS, U.

Table S9. Contributions to the Life Cycle Inventory (LCI) for the production of 1p of separatory funnel. The lifetime of the separatory funnel was assumed to be 28800 h (considering 15 years of use for 48 week per year, 5 days per week and 8 hours per day). The plastic portion of the separatory funnel, its stopcock, was also considered, being made of tetrafluoroethylene.

Description			Amount	Process Data Source
Input	Material	Borosilicate glass	65.79 g	Ecoinvent v 3.8[1]
		TFE	10.93 g	Ecoinvent v 3.8[2]
	Transport	Transport of raw materials	7.67 kgkm	Ecoinvent v 3.8[3]
Output	End of life	Landfill	65.79 g	Ecoinvent v 3.8[4]
		Recycling of plastic	10.93 g	Ecoinvent v 3.8[5]

[1]The Ecoinvent process used was: Glass tube, borosilicate {RoW}| production | APOS, U.
[2]The Ecoinvent process used was: Tetrafluoroethylene {RER}| production | APOS, U.

[3]The Ecoinvent process used was: Transport, freight, lorry 3.5-7.5 metric ton, EURO6 {RER}| transport, freight, lorry 3.5-7.5 metric ton, EURO6 | APOS, U. An average distance of 100km was considered.
[4]The Ecoinvent process used was: Inert waste, for final disposal {RoW}| treatment of inert waste, inert material landfill APOS, U.
[5]The recycling process for plastic was modified by the authors. Particularly a new process was created with the inputs taken from the Ecoinvent database process of the Polypropylene recycling. However, the environmental loads were allocated to both the recycling process and the secondary recycled plastic with a contribution of 50 % each.

Table S10. Contributions to the Life Cycle Inventory (LCI) for the production of 1p of glass round bottom flask. The lifetime of the round bottom flask was assumed to be 28800 h (considering 15 years of use for 48 week per year, 5 days per week and 8 hours per day).

Description			Amount	Process Data Source
Input	Material	Borosilicate glass	71.05 g	Ecoinvent v 3.8[1]
	Transport	Transport of raw materials	7.105 kgkm	Ecoinvent v 3.8[2]
Output	End of life	Landfill	71.05 g	Ecoinvent v 3.8[3]

[1]The Ecoinvent process used was: Glass tube, borosilicate {RoW}| production | APOS, U.
[2]The Ecoinvent process used was: Transport, freight, lorry 3.5-7.5 metric ton, EURO6 {RER}| transport, freight, lorry 3.5-7.5 metric ton, EURO6 | APOS, U. An average distance of 100km was considered.
[3]The Ecoinvent process used was: Inert waste, for final disposal {RoW}| treatment of inert waste, inert material landfill APOS, U.

Table S11. Contributions to the Life Cycle Inventory (LCI) for the production of 1p glass fitting for the rotavapor. The lifetime of the fitting for the rotavapor was assumed to be 28800 h (considering 15 years of use for 48 week per year, 5 days per week and 8 hours per day).

Description			Amount	Process Data Source
Input	Material	Borosilicate glass	29.74 g	Ecoinvent v 3.8[1]
	Transport	Transport of raw materials	2.974 kgkm	Ecoinvent v 3.8[2]
Output	End of life	Landfill	29.74 g	Ecoinvent v 3.8[3]

[1]The Ecoinvent process used was: Glass tube, borosilicate {RoW}| production | APOS, U.
[2]The Ecoinvent process used was: Transport, freight, lorry 3.5-7.5 metric ton, EURO6 {RER}| transport, freight, lorry 3.5-7.5 metric ton, EURO6 | APOS, U. An average distance of 100km was considered.
[3]The Ecoinvent process used was: Inert waste, for final disposal {RoW}| treatment of inert waste, inert material landfill APOS, U.

Table S12. Contributions to the Life Cycle Inventory (LCI) for the production of 1p glass fitting for the reflux condenser. The lifetime of the fitting for reflux condenser was assumed to be 28800 h (considering 15 years of use for 48 week per year, 5 days per week and 8 hours per day).

Description			Amount	Process Data Source
Input	Material	Borosilicate glass	31.7 g	Ecoinvent v 3.8[1]
	Transport	Transport of raw materials	3.17 kgkm	Ecoinvent v 3.8[2]
Output	End of life	Landfill	31.7 g	Ecoinvent v 3.8[3]

[1]The Ecoinvent process used was: Glass tube, borosilicate {RoW}| production | APOS, U.

[2]The Ecoinvent process used was: Transport, freight, lorry 3.5-7.5 metric ton, EURO6 {RER}| transport, freight, lorry 3.5-7.5 metric ton, EURO6 | APOS, U. An average distance of 100km was considered.

[3]The Ecoinvent process used was: Inert waste, for final disposal {RoW}| treatment of inert waste, inert material landfill APOS, U.

Table S13. Contributions to the Life Cycle Inventory (LCI) for the production of 1p glass reflux condenser. The lifetime of the reflux condenser was assumed to be 28800 h (considering 15 years of use for 48 week per year, 5 days per week and 8 hours per day).

Description			Amount	Process Data Source
Input	Material	Borosilicate glass	222.73 g	Ecoinvent v 3.8[1]
	Transport	Transport of raw materials	22.273 kgkm	Ecoinvent v 3.8[2]
Output	End of life	Landfill	222.73 g	Ecoinvent v 3.8[3]

[1]The Ecoinvent process used was: Glass tube, borosilicate {RoW}| production | APOS, U.

[2]The Ecoinvent process used was: Transport, freight, lorry 3.5-7.5 metric ton, EURO6 {RER}| transport, freight, lorry 3.5-7.5 metric ton, EURO6 | APOS, U. An average distance of 100km was considered.

[3]The Ecoinvent process used was: Inert waste, for final disposal {RoW}| treatment of inert waste, inert material landfill APOS, U.

Table S14. Contributions to the Life Cycle Inventory (LCI) for the production of 1p of rubber tube. The lifetime of the rubber tube was assumed to be 15 years, 131400 hours.

Description			Amount	Process Data Source
Input	Material	Synthetic rubber	83.19 g	Ecoinvent v 3.8[1]
	Transport	Transport of raw materials	8.319 kgkm	Ecoinvent v 3.8[2]
Output	End of life	Recycling od plastics	83.19 g	Ecoinvent v 3.8[3]

[1]The Ecoinvent process used was: Synthetic rubber {RER}| production | APOS, U.

[2]The Ecoinvent process used was: Transport, freight, lorry 3.5-7.5 metric ton, EURO6 {RER}| transport, freight, lorry 3.5-7.5 metric ton, EURO6 | APOS, U. An average distance of 100km was considered.

[3]The recycling process for plastic was modified by the authors. Particularly a new process was created with the inputs taken from the Ecoinvent database process of the Polypropylene recycling. However, the environmental loads were allocated to both the recycling process and the secondary recycled plastic with a contribution of 50 % each.

Table S15. Contributions to the Life Cycle Inventory (LCI) for the production of 1p of a round bottom flask used during the workup phase. The lifetime of the round bottom flaks was assumed to be 28800 h (considering 15 years of use for 48 week per year, 5 days per week and 8 hours per day).

Description			Amount	Process Data Source
Input	Material	Borosilicate glass	19.16 g	Ecoinvent v 3.8[1]
	Transport	Transport of raw materials	1.92 kgkm	Ecoinvent v 3.8[2]
Output	End of life	Landfill	19.16 g	Ecoinvent v 3.8[3]

[1]The Ecoinvent process used was: Glass tube, borosilicate {RoW}| production | APOS, U.
[2]The Ecoinvent process used was: Transport, freight, lorry 3.5-7.5 metric ton, EURO6 {RER}| transport, freight, lorry 3.5-7.5 metric ton, EURO6 | APOS, U. An average distance of 100km was considered.
[3]The Ecoinvent process used was: Inert waste, for final disposal {RoW}| treatment of inert waste, inert material landfill APOS, U.

Table S16. Contributions to the Life Cycle Inventory (LCI) for the production of 1p of a round bottom flask. The lifetime of the round bottom flaks was assumed to be 28800 h (considering 15 years of use for 48 week per year, 5 days per week and 8 hours per day).

Description			Amount	Process Data Source
Input	Material	Borosilicate glass	43.61 g	Ecoinvent v 3.8[1]
	Transport	Transport of raw materials	4.361 kgkm	Ecoinvent v 3.8[2]
Output	End of life	Landfill	43.61 g	Ecoinvent v 3.8[3]

[1]The Ecoinvent process used was: Glass tube, borosilicate {RoW}| production | APOS, U.
[2]The Ecoinvent process used was: Transport, freight, lorry 3.5-7.5 metric ton, EURO6 {RER}| transport, freight, lorry 3.5-7.5 metric ton, EURO6 | APOS, U. An average distance of 100km was considered.
[3]The Ecoinvent process used was: Inert waste, for final disposal {RoW}| treatment of inert waste, inert material landfill APOS, U.

Table S17. Contributions to the Life Cycle Inventory (LCI) for the production of 1p of a distillation column. The lifetime of the distillation column was assumed to be 28800 h (considering 15 years of use for 48 week per year, 5 days per week and 8 hours per day).

Description			Amount	Process Data Source
Input	Material	Borosilicate glass	350 g	Ecoinvent v 3.8[1]

	Transport	Transport of raw materials	35 kgkm	Ecoinvent v 3.8[2]
Output	End of life	Landfill	350 g	Ecoinvent v 3.8[3]

[1]The Ecoinvent process used was: Glass tube, borosilicate {RoW}| production | APOS, U.
[2]The Ecoinvent process used was: Transport, freight, lorry 3.5-7.5 metric ton, EURO6 {RER}| transport, freight, lorry 3.5-7.5 metric ton, EURO6 | APOS, U. An average distance of 100km was considered.
[3]The Ecoinvent process used was: Inert waste, for final disposal {RoW}| treatment of inert waste, inert material landfill APOS, U.

Table S18. Contributions to the Life Cycle Inventory (LCI) for the production of 1p of the magnetic stirrer, with a weight of 2.8 kg and a power of 500W. Its lifetime was assumed to be 50000 h.

Description			Amount	Process data source
Input	Material	Reinforcing Steel	1123.07 g	Ecoinvent v. 3.8[1]
		HDPE	980 g	Ecoinvent v. 3.8[2]
		Steel	420 g	Ecoinvent v. 3.8[3]
		Copper	280 g	Ecoinvent v. 3.8[4]
	Process	Hot Rolling	1123.07 g	Ecoinvent v. 3.8[5]
		Blow Moulding	980 g	Ecoinvent v. 3.8[6]
		Wire Drawing (steel)	420 g	Ecoinvent v. 3.8[7]
		Wire drawing (copper)	280 g	Ecoinvent v. 3.8[8]
	Transport	Transport of raw materials	280.31 kgkm	Ecoinvent v. 3.8[9]
Output	End of life	Waste treatment	2.8 kg	Ecoinvent v. 3.8[10]

[1]The Ecoinvent process used was: Reinforcing steel {Europe without Austria}| reinforcing steel production | APOS, U.
[2]The Ecoinvent process used was: Polyethylene, high density, granulate {RER}| production | APOS, U.
[3]The Ecoinvent process used was: Steel, chromium steel 18/8 {RER}| steel production, electric, chromium steel 18/8 | APOS, U.
[4]The Ecoinvent process used was: Copper {RER}| production, primary | APOS, U.
[5]The Ecoinvent process used was: Hot rolling, steel {RoW}| processing | APOS, U.
[6]The Ecoinvent process used was: Blow moulding {RER}| blow moulding | APOS, U.
[7]The Ecoinvent process used was: Wire drawing, steel {RER}| processing | APOS, U.
[8]The Ecoinvent process used was: Wire drawing, copper {RoW}| processing | APOS, U.
[9]The Ecoinvent process used was: Transport, freight, lorry 16-32 metric ton, EURO6 {RER}| transport, freight, lorry 16-32 metric ton, EURO6 | APOS, U. An average distance of 100 km was chosen.
[10]The Ecoinvent process used was: Waste electric and electronic equipment {GLO}| treatment of, shredding | APOS, U.

Table S19. Contributions to the Life Cycle Inventory (LCI) foe the synthesis of 12.21 g of 4-hydroxybenzaldehyde, according to the synthesis proposed by the patent CN102992982A, based on the Vilsmeier reaction.

Description			Amount	Process Data Source
Input	Materials	Toluene	87.14 g	Ecoinvent v 3.8[1]
		$POCl_3$	19.762 g	Ecoinvent v 3.8[2]
		DMF	9.4 g	Ecoinvent v 3.8[3]
		Phenol	10.15 g	Ecoinvent v 3.8[4]
		Water	200g	Ecoinvent v 3.8[5]
		Water	280 g	Ecoinvent v 3.8[6]
	Equipment/ plants	Magnetic stirrer/heater	$5.83*10^{-5}$ p	Modelled from Ecoinvent v 3.8 database sub process as detailed in Table S18
		Round bottom flask	$1.04*10^{-4}$ p	Modelled from Ecoinvent v 3.8 database sub process as detailed in Table S16
		Separatory funnel	$8.68*10^{-6}$ p	Modelled from Ecoinvent v 3.8 database sub process as detailed in Table S9
		Activated carbon air filter	$1.5*10^{-4}$ p	Modelled from Ecoinvent v 3.8 database sub process as detailed in Table S4
		Aspiration system	$3.76*10^{-5}$ p	Modelled from Ecoinvent v 3.8 database sub process as detailed in Table S3
	Trasnport	Transport for large equipment	$6.26*10^{-3}$ kgkm	Ecoinvent v 3.8[7]
		Transport for raw materials	60.6321 kgkm	Ecoinvent v 3.8[8]
		Transport for small equipment	$8.22*10^{-2}$kgkm	Ecoinvent v 3.8[9]
	Energy	Electric energy	62.20 J	Ecoinvent v 3.8[10]
			27 Wh	Ecoinvent v 3.8[11]
			250 Wh	Ecoinvent v 3.8[12]
			835 Wh	Ecoinvent v 3.8[13]
			41 kJ	Ecoinvent v 3.8[14]
			35.94 kJ	Ecoinvent v 3.8[15]
			$2.51*10^{-1}$ kWh	Ecoinvent v 3.8[16]

Output	Air emission	Toluene	$1.41*10^{-3}$ g	SimaPro airborne emission substance list[17]
		DMF	$1.46*10^{-6}$ g	SimaPro airborne emission substance list[18]
		Phenol	$1.68*10^{-7}$ g	SimaPro airborne emission substance list[19]
		Water	$4.61*10^{-5}$ g	SimaPro airborne emission substance list[20]
		Water	$6.46*10^{-5}$ g	SimaPro airborne emission substance list[21]
	End of life	Waste water	200 g	Ecoinvent v 3.8[22]
		Waste water	303.34 cm^3	Ecoinvent v 3.8[23]
		Spent solvent mixture	87 g	Ecoinvent v 3.8[24]

[1]The Ecoinvnet process used was: Toluene, liquid {RER}| production | APOS, U.

[2]The Ecoinvent process used was: Phosphorus oxychloride {RER}| phosphorus oxychloride production, from phosphorus trichloride | APOS, U.

[3]The Ecoinvent process used was: N,N-dimethylformamide {RER}| production | APOS, U.

[4]The Ecoinvent process use was: Phenol {RER}| phenol production, from cumene | APOS, U.

[5]The Ecoinvent process used was: Water, deionised {Europe without Switzerland} | water production, deionised | APOS, U.

[6]The Ecoinvent process used was: Water, deionised {Europe without Switzerland} | water production, deionised | APOS, U.

[7]Transport of large equipment (aspiration system and activated air carbon filter). The Ecoinvet process used was: Transport, freight, lorry 16-32 metric ton, EURO6 {RER}| transport, freight, lorry 16-32 metric ton, EURO6 | APOS, U. An average distance of 100km was considered.

[8]Transport for reagents. The Ecoinvent process used was: Transport, freight, lorry 3.5-7.5 metric ton, EURO6 {RER}| transport, freight, lorry 3.5-7.5 metric ton, EURO6 | APOS, U. An average distance of 100km was considered.

[9]Transport for small laboratory equipment. The Ecoinvent process used was: Transport, freight, lorry 3.5-7.5 metric ton, EURO6 {RER}| transport, freight, lorry 3.5-7.5 metric ton, EURO6 | APOS, U. An average distance of 100 km was considered.

[10]Electric energy necessary for the first reaction step to mix and cool down to 10°C the reaction mixture containing Toluene, DMF and $POCl_3$. It is calculated using the expression $m*Cp*DT$, considering the specific heat in J/kg°C for each of the three substances.

[11]Electric energy necessary to maintain the agitation and cooling down of the reaction mixture, in the first reaction step, for 45 minutes. It is calculated using the Fourier Equation. The Ecoinvent process used was: Electricity, low voltage {IT}| electricity voltage transformation from medium to low voltage | APOS, U.

[12]Electric energy necessary for the mixing and heating of the reaction mixture in the second step of the reaction. It is calculated as power*time, considering a power of 200W to heat the reaction to a temperature of 120°C.The Ecoinvent process used was: Electricity, low voltage {IT}| electricity voltage transformation from medium to low voltage | APOS, U.

[13]Energy necessary to mix the reaction for 100 minutes after adding 200 ml of water. It is calculated as power*time. The Ecoinvent process used was: Electricity, low voltage {IT}| electricity voltage transformation from medium to low voltage | APOS, U.

[14]Electric energy necessary to heat up to 60°C 280 ml of water. It is calculated as $m*Cp*DT$, considering 4.186 J/g°C as water specific heat. The Ecoinvent process used was: Electricity, low voltage {IT}| electricity voltage transformation from medium to low voltage | APOS, U.

[15]Electric energy necessary to eliminate the remaining solvent (Toluene) using the rotavapor. It is calculated as HvapToluene*molTtoluene, considering HvaptToluene as its latent heat of vaporization (38.06 kJ/mol) and molToluene as the moles of the solvent. The Ecoinvent process used was: Electricity, low voltage {IT}| electricity voltage transformation from medium to low voltage | APOS, U.
[16]Electric energy necessary to the use of the aspiration system for the whole synthesis time, i.e. 3 h. The power was calculated by considering the air flow rate of 250 m3/h, a total load loss of 110.8076 kg/m2, and an efficiency of 90%: Electricity, low voltage {IT}| electricity voltage transformation from medium to low voltage | APOS, U.
[17]Amount of Toluene released into the atmosphere, as calculated by the formula reported in equation 1 of the main manuscript.
[18] Amount of DMF released into the atmosphere, as calculated by the formula reported in equation 4 of the main manuscript.
[19] Amount of Phenol released into the atmosphere, as calculated by the formula reported in equation 4 of the main manuscript.
[20]Amount of water released into the atmosphere, as calculated by the formula reported in equation 4 of the main manuscript.
[21]Amount of water released into the atmosphere, as calculated by the formula reported in equation 4 of the main manuscript.
[22]End of life of water separated from the organic phase using a separatory funnel. The Ecoinvent process used was: Wastewater, average {Europe without Switzerland} | treatment of wastewater, average, capacity 1E9l/year | APOS, U.
[23]End of life of water separated with a separatory funnel from the organic phase in the last step of the reaction. The Ecoinvent process used was: Wastewater, average {Europe without Switzerland} | treatment of wastewater, average, capacity 1E9l/year | APOS, U.
[24]End of life of the Toluene solvent used to carry the reaction. The Ecoinvent process used was: Spent solvent mixture {Europe without Switzerland} | treatment of spent solvent mixture, hazardous waste incineration, with energy recovery | APOS, U

Table S20. Contribution to the Life Cycle Inventory (LCI) for the synthesis of $8.72*10^{-2}$ g of Vanillin, according to t the synthesis proposed by Taber et all [1].

Description			Amount	Process Data Source
Input	Materials	4-hydroxy benzaldehyde	0.1 g	Modelled from Ecoinvent v 3.8 database sub process as detailed in Table S19
		Br_2	2.50 g	Ecoinvent v 3.8[1]
		CH_3OH	0.70 g	Ecoinvent v 3.8[2]
		CH_3NaO	0.33 g	Ecoinvent v 3.8[3]
		CH_3OH	0.99 g	Ecoinvent v 3.8[4]
		Ethyl acetate	0.99 g	Ecoinvent v 3.8[5]
		CuBr	0.08 g	Ecoinvent v 3.8[6]
		HCl	$1.38*10^{-2}$ g	Ecoinvent v 3.8[7]
		Ethyl acetate	13.51 g	Ecoinvent v 3.8[8]
		Na_2SO_4	5 g	Ecoinvent v 3.8[9]
		Diethyl ether	149.1 g	Ecoinvent v 3.8[10]
		H_2O	25 g	Ecoinvent v 3.8[11]
		Silicon oil	0.26 g	Ecoinvent v 3.8[12]
		Activated silica	5.5 g	Ecoinvent v 3.8[13]

	Equipment/ plants	Magnetic stirrer/heater	$3*10^{-5}$ p	Modelled from Ecoinvent v 3.8 database sub process as detailed in Table S18
		Round bottom flask	$1.77*10^{-5}$ p	Modelled from Ecoinvent v 3.8 database sub process as detailed in Table S16
		Separatory funnel	$1.77*10^{-5}$ p	Modelled from Ecoinvent v 3.8 database sub process as detailed in Table S9
		Activated carbon air filter	$1.5*10^{-4}$ p	Modelled from Ecoinvent v 3.8 database sub process as detailed in Table S4
		Aspiration system	$3.76*10^{-5}$ p	Modelled from Ecoinvent v 3.8 database sub process as detailed in Table S3
	Trasnport	Transport for large equipment	$6.26*10^{-3}$ kgkm	Ecoinvent v 3.8[14]
		Transport for raw materials	20.32 kgkm	Ecoinvent v 3.8[15]
		Transport for small equipment	$9.30 *10^{-3}$ kgkm	Ecoinvent v 3.8[16]
	Energy	Electric energy	6.51 kJ	Ecoinvent v 3.8[17]
			25.2 Wh	Ecoinvent v 3.8[18]
			16.81 kJ	Ecoinvent v 3.8[19]
			252 Wh	Ecoinvent v 3.8[20]
			4.97 kJ	Ecoinvent v 3.8[21]
			0.25 kJ	Ecoinvent v 3.8[22]
			7.84 kJ	Ecoinvent v 3.8[23]
Output	Air emission	Br_2	$1.20*10^{-5}$ g	SimaPro airborne emission substance list[24]
		Ethyl acetate	$5.21*10^{-5}$ g	SimaPro airborne emission substance list[25]
		HCl	$4.83*10^{-5}$ g	SimaPro airborne emission substance list[26]
		CH_3OH	$1.16*10^{-3}$ g	SimaPro airborne emission substance list[27]

End of life	Waste water	0.025 l	Ecoinvent v 3.8[28]
	Spent solvent mixture	166.65 g	Ecoinvent v 3.8[29]
	Spent solvent mixture	5.18 g	Ecoinvent v 3.8[30]
	Incineration	5.76 g	Ecoinvent v 3.8[31]

[1]The Ecoinvnet process used was: Bromine {RER}∣ production ∣ APOS, U.
[2]The Ecoinvent process used was: Methanol {GLO}∣ production ∣ APOS, U.
[3]The Ecoinvent process used was: Sodium methoxide {GLO}∣ production ∣ APOS, U.
[4]The Ecoinvent process use was: Methanol {GLO}∣ production ∣ APOS, U.
[5]The Ecoinvent process used was: Ethyl acetate {RER}∣ production ∣ APOS, U.
[6]The Ecoinvent process used was: Copper oxide {RER}∣ production ∣ APOS, U. Since CuBr is not present in the database, the Ecoinvent process for CuO was used as proxy.
[7]The Ecoinvent process used was: Hydrochloric acid, without water, in 30% solution state {RER}∣ hydrochloric acid production, from the reaction of hydrogen with chlorine ∣ APOS, U.
[8]The Ecoinvent process used was: Ethyl acetate {RER}∣ production ∣ APOS, U.
[9]The Ecoinvent process used was: Sodium sulphate, anhydrite {RER}∣ sodium sulphate production, from natural sources ∣ APOS, U.
[10]The Ecoinvent process used was: Diethyl ether, without water, in 99.95% solution state {RER}∣ ethylene hydration ∣ APOS, U.
[11]The Ecoinvent process used was: Water, deionised {Europe without Switzerland}∣ water production, deionised ∣ APOS, U.
[12]The Ecoinvent process used was: Silicone product {RER}∣ production ∣ APOS, U.
[13]The Ecoinvent process used was: Activated silica {GLO}∣ production ∣ APOS, U.
[14]Transport of large equipment (aspiration system and activated air carbon filter). The Ecoinvet process used was: Transport, freight, lorry 16-32 metric ton, EURO6 {RER}∣ transport, freight, lorry 16-32 metric ton, EURO6 ∣ APOS, U. An average distance of 100km was considered.
[15]Transport for reagents. The Ecoinvent process used was: Transport, freight, lorry 3.5-7.5 metric ton, EURO6 {RER}∣ transport, freight, lorry 3.5-7.5 metric ton, EURO6 ∣ APOS, U. An average distance of 100km was considered.
[16]Transport for small laboratory equipment. The Ecoinvent process used was: Transport, freight, lorry 3.5-7.5 metric ton, EURO6 {RER}∣ transport, freight, lorry 3.5-7.5 metric ton, EURO6 ∣ APOS, U. An average distance of 100 km was considered.
[17]Electric energy necessary for the heat up to 130°Cof the reaction mixture in vial B containing methanol, ethyl acetate, sodium methoxide and copper. It is calculated using the expression *m*Cp*DT*, considering the specific heat in J/kg°C for each of the substances. The Ecoinvent process used was: Electricity, low voltage {IT}∣ electricity voltage transformation from medium to low voltage ∣ APOS, U.
[18]Electric energy necessary to maintain the heating at 130°C of the reaction mixture in vial B for six hours. It is calculated using the Fourier Equation. The Ecoinvent process used was: Electricity, low voltage {IT}∣ electricity voltage transformation from medium to low voltage ∣ APOS, U.
[19]Electric energy necessary for the heating of vial B during the adding of 4-hydroxy benzaldehyde and vial A to the reaction mixture up to 130°C. It is calculated using the expression *m*Cp*DT*, considering the specific heat in J/kg°C for each of the substances. The Ecoinvent process used was: Electricity, low voltage {IT}∣ electricity voltage transformation from medium to low voltage ∣ APOS, U.
[20]Electric energy necessary to maintain the heating at 130°C of the reaction mixture for one hour.. It is calculated using the Fourier Equation. The Ecoinvent process used was: Electricity, low voltage {IT}∣ electricity voltage transformation from medium to low voltage ∣ APOS, U.
[21]Electric energy necessary to eliminate the remaining solvent (ethyl acetate) using the rotavapor. It is calculated as HvapEthylAcetate*MEthylAcetate, considering HvapEthylAcetate as its latent heat of vaporization (365.7 J/g) and MEthylAcetate as the mass of the used solvent. The Ecoinvent process used was: Electricity, low voltage {IT}∣ electricity voltage transformation from medium to low voltage ∣ APOS, U.
[22]Electric energy necessary to the use of the aspiration system for the whole synthesis time, i.e. 3 h. The power was calculated by considering the air flow rate of 250 m3/h, a total load loss of 110.8076 kg/m2, and an efficiency of 90%: Electricity, low voltage {IT}∣ electricity voltage transformation from medium to low voltage ∣ APOS, U.

[23]Electric energy necessary to heat up to its boiling point the water used for the crystallization step. It is calculated as *m*Cp*DT*, considering 4.186 J/g°C as water specific heat. The Ecoinvent process used was: Electricity, low voltage {IT}| electricity voltage transformation from medium to low voltage | APOS, U.
[24]Amount of Bromine released into the atmosphere, as calculated by the formula reported in equation 4 of the main manuscript.
[25]Amount of Ethyl Acetate released into the atmosphere, as calculated by the formula reported in equation 4 of the main manuscript.
[26]Amount of HCl released into the atmosphere, as calculated by the formula reported in equation 4 of the main manuscript.
[27]Amount of Methanol released into the atmosphere, as calculated by the formula reported in equation 4 of the main manuscript.
[28]End of life of water used during the crystallization phase. The Ecoinvent process used was: Wastewater, average {Europe without Switzerland} | treatment of wastewater, average, capacity 1E9l/year | APOS, U.
[29]End of life of the solvents used to carry the reaction. The Ecoinvent process used was: Spent solvent mixture {Europe without Switzerland} | treatment of spent solvent mixture, hazardous waste incineration, with energy recovery | APOS, U.
[30]End of life of HCl with the inorganic solid waste. The Ecoinvent process used was: Spent solvent mixture {Europe without Switzerland} | treatment of spent solvent mixture, hazardous waste incineration, with energy recovery | APOS, U.
[31]End of life of the waste of the silica gel from the chromatography phase and the silicon oil. The Ecoinvent process used was: Hazardous waste, for incineration {RoW}| treatment of hazardous waste, hazardous waste incineration, with energy recovery | APOS, U.

Table S21. Contribution to the Life Cycle Inventory (LCI) for the synthesis of 161.82 g of Hydrobromic Acid (HBr) according to Yoffe et al. [2].

Description			Amount	Process Data Source
Input	Materials	SO_2	64.07 g	Ecoinvent v 3.8[1]
		Br_2	159.81 g	Ecoinvent v 3.8[2]
		H_2O	36 g	Ecoinvent v 3.8[3]
	Equipment/ plants	Magnetic stirrer/heater	$4*10^{-5}$ p	Modelled from Ecoinvent v 3.8 database sub process as detailed in Table S18
		Activated carbon air filter	$1*10^{-4}$ p	Modelled from Ecoinvent v 3.8 database sub process as detailed in Table S4
		Aspiration system	$2.51*10^{-5}$ p	Modelled from Ecoinvent v 3.8 database sub process as detailed in Table S3
	Trasnport	Transport for large equipment	$4.17*10^{-3}$ tkm	Ecoinvent v 3.8[4]
		Transport for raw materials	25.99 kgkm	Ecoinvent v 3.8[5]
	Energy	Electric energy	5.67 kJ	Ecoinvent v 3.8[6]
			60 Wh	Ecoinvent v 3.8[7]
			14.77 kJ	Ecoinvent v 3.8[8]

			238.32 Wh	Ecoinvent v 3.8[9]
Output	Air emission	Br_2	$2.53*10^{-3}$ g	SimaPro airborne emission substance list[10]
		SO_2	$5.37*10^{-3}$ g	SimaPro airborne emission substance list[11]
		H_2O	$4.15*10^{-6}$ g	SimaPro airborne emission substance list[12]
	End of life	Waste water	53.60 cm^3	Ecoinvent v 3.8[13]

[1]The Ecoinvent process used was: Sulfur dioxide, liquid {RER}| production | APOS, U.
[2]The Ecoinvent process used was: Bromine {RER}| production | APOS, U.
[3]The Ecoinvnet process used was: Water, deionised {Europe without Switzerland}| water production, deionised | APOS, U.
[4]Transport of large equipment (aspiration system and activated air carbon filter). The Ecoinvet process used was: Transport, freight, lorry 16-32 metric ton, EURO6 {RER}| transport, freight, lorry 16-32 metric ton, EURO6 | APOS, U. An average distance of 100km was considered.
[5]Transport for reagents. The Ecoinvent process used was: Transport, freight, lorry 3.5-7.5 metric ton, EURO6 {RER}| transport, freight, lorry 3.5-7.5 metric ton, EURO6 | APOS, U. An average distance of 100km was considered.
[6]Electric energy necessary for the heating of the reaction mixture up to 50°C. It is calculated using the expression *m*Cp*DT*, considering the specific heat in J/kg°C for each of the substances (Br_2, SO_2 and H_2O). The Ecoinvent process used was: Electricity, low voltage {IT}| electricity voltage transformation from medium to low voltage | APOS, U.
[7]Electric energy necessary to maintain the heating at 50°C of the reaction mixture for one hour. It is calculated using the Fourier Equation. The Ecoinvent process used was: Electricity, low voltage {IT}| electricity voltage transformation from medium to low voltage | APOS, U.
[8]Electric energy necessary to separate HBr and H_2SO_4 at the end of the synthesis with a distillation process. It is calculated as *m*Cp*DT*, considering the specific heat in J/mol°C for each of the acid. The temperature considered is the distillation temperature of HBr, 124.3°C. The Ecoinvent process used was: Electricity, low voltage {IT}| electricity voltage transformation from medium to low voltage | APOS, U.
[9]Amount of Bromine released into the atmosphere, as calculated by the formula reported in equation 4 of the main manuscript.
[10]Amount of Sulfur dioxide released into the atmosphere, as calculated by the formula reported in equation 4 of the main manuscript.
[11]Amount of Water released into the atmosphere, as calculated by the formula reported in equation 4 of the main manuscript.
[12]End of life of Sulphuric acid obtained after the distillation process. The Ecoinvent process used was: Wastewater, average {Europe without Switzerland} | treatment of wastewater, average, capacity 1E9l/year | APOS, U.

Table S22. Contributions to the Life Cycle Inventory (LCI) for the synthesis of 712.16 g of Sodium Bromide (NaBr) according to the Patent CN103395806A.

Description			Amount	Process Data Source
Input	Materials	HBr	560 g	Modelled from Ecoinvent v 3.8 database sub process as detailed in Table S21
		NaOH	450 g	Ecoinvent v 3.8[1]
		H_2O	1 l	Ecoinvent v 3.8[2]

		H_2O	1.4 l	Ecoinvent v 3.8[3]
	Equipment/ plants	Magnetic stirrer/heater	5*10^{-5} p	Modelled from Ecoinvent v 3.8 database sub process as detailed in Table S18
		Round bottom flask	8.68*10^{-5} p	Modelled from Ecoinvent v 3.8 database sub process as detailed in Table S16
		Activated carbon air filter	1.25*10^{-4} p	Modelled from Ecoinvent v 3.8 database sub process as detailed in Table S4
		Aspiration system	3.13*10^{-5} p	Modelled from Ecoinvent v 3.8 database sub process as detailed in Table S3
	Transport	Transport for large equipment	5.21*10^{-3} tkm	Ecoinvent v 3.8[4]
		Transport of small equipment	1.44*10^{-2} kgkm	Ecoinvent v 3.8[5]
		Transport for raw materials	341 kgkm	Ecoinvent v 3.8[6]
	Energy	Electric energy	6 Wh	Ecoinvent v 3.8[7]
			470 kJ	Ecoinvent v 3.8[8]
			558 Wh	Ecoinvent v 3.8[9]
Output	Air emission	H_2O	2.05 g	SimaPro airborne emission substance list[10]
	End of life	Waste water	3.11*10^{3} cm^{3}	Ecoinvent v 3.8[11]

[1]The Ecoinvent process used was: Sodium hydroxide, without water, in 50% solution state {RER}| chlor-alkali electrolysis, diaphragm cell | APOS, U.

[2]The Ecoinvent process used was: Water, deionised {Europe without Switzerland}| water production, deionised | APOS, U.

[3]The Ecoinvent process used was: Water, deionised {Europe without Switzerland}| water production, deionised | APOS, U.

[4]Transport of large equipment (aspiration system and activated air carbon filter). The Ecoinvet process used was: Transport, freight, lorry 16-32 metric ton, EURO6 {RER}| transport, freight, lorry 16-32 metric ton, EURO6 | APOS, U. An average distance of 100km was considered.

[5]Transport for small equipment. The Ecoinvent process used was: Transport, freight, lorry 3.5-7.5 metric ton, EURO6 {RER}| transport, freight, lorry 3.5-7.5 metric ton, EURO6 | APOS, U. An average distance of 100km was considered.

[6]Transport for reagents. The Ecoinvent process used was: Transport, freight, lorry 3.5-7.5 metric ton, EURO6 {RER}| transport, freight, lorry 3.5-7.5 metric ton, EURO6 | APOS, U. An average distance of 100km was considered.

[7]Electric energy necessary for the sole agitation of the reaction mixture for one hour. It is calculated as power*time, considering the power of the sole agitation as 6W. The Ecoinvent process used was: Electricity, low voltage {IT}| electricity voltage transformation from medium to low voltage | APOS, U.

[8]Electric energy necessary for the heating to 180°C of NaBr and water during the crystallization phase. It is calculated using the expression *m*Cp*DT*, considering the specific heat in J/kg°C for each of the substances (NaBr and H_2O). The Ecoinvent process used was: Electricity, low voltage {IT}| electricity voltage transformation from medium to low voltage | APOS, U.
[9]Electric energy necessary to maintain the heating at 180°C for one hour and half. It is calculated using the Fourier Equation. The Ecoinvent process used was: Electricity, low voltage {IT}| electricity voltage transformation from medium to low voltage | APOS, U.
[10]Amount of Water released into the atmosphere, as calculated by the formula reported in equation 4 of the main manuscript.
[11]End of life of the water produced during the reaction. The Ecoinvent process used was: Wastewater, average {Europe without Switzerland} | treatment of wastewater, average, capacity 1E9l/year | APOS, U.

Table S23. Contributions to the Life Cycle Inventory (LCI) for the synthesis of 14.94 g of Bromobutane according to Williams et al. [3].

Description			Amount	Process Data Source
Input	Materials	NaBr	13.3 g	Modelled from Ecoinvent v 3.8 database sub process as detailed in Table S22
		H_2O	15 g	Ecoinvent v 3.8[1]
		1-butanol	8.10 g	Ecoinvent v 3.8[2]
		H_2SO_4	21.045 g	Ecoinvent v 3.8[3]
		H_2O	10 g	Ecoinvent v 3.8[4]
		H_2SO_4	18.3 g	Ecoinvent v 3.8[5]
		NaOH	21.3 g	Ecoinvent v 3.8[6]
		$CaCl_2$	1 g	Ecoinvent v 3.8[7]
		Xylene	17.80 g	Ecoinvent v 3.8[8]
	Equipment/ plants	Magnetic stirrer/heater	$6*10^{-5}$ p	Modelled from Ecoinvent v 3.8 database sub process as detailed in Table S18
		Round bottom flask	$6.94*10^{-5}$ p	Modelled from Ecoinvent v 3.8 database sub process as detailed in Table S15
		Separatory funnel	$8.68*10^{-6}$ p	Modelled from Ecoinvent v 3.8 database sub process as detailed in Table S9
		Reflux condenser	$2.61*10^{-5}$ p	Modelled from Ecoinvent v 3.8 database sub process as detailed in Table S13
		Distillation column	$6.94*10^{-5}$ p	Modelled from Ecoinvent v 3.8 database

				sub process as detailed in Table S17
		Flask	$1.74*10^{-5}$ p	Modelled from Ecoinvent v 3.8 database sub process as detailed in Table S5
		Rubber tube	$1.70*10^{-4}$ p	Modelled from Ecoinvent v 3.8 database sub process as detailed in Table S14
		Activated carbon air filter	$2*10^{-4}$ p	Modelled from Ecoinvent v 3.8 database sub process as detailed in Table S4
		Aspiration system	$5.02*10^{-5}$ p	Modelled from Ecoinvent v 3.8 database sub process as detailed in Table S3
	Transport	Transport for large equipment	$8.34*10^{-3}$ kgkm	Ecoinvent v 3.8[9]
		Transport for raw materials	12.58 kgkm	Ecoinvent v 3.8[10]
		Transport for small equipment	$2.08 *10^{-2}$ kgkm	Ecoinvent v 3.8[11]
	Energy	Electric energy	2.5 kJ	Ecoinvent v 3.8[12]
			17.465 kJ	Ecoinvent v 3.8[13]
			315 Wh	Ecoinvent v 3.8[14]
			26.51 kJ	Ecoinvent v 3.8[15]
			12.49 kJ	Ecoinvent v 3.8[16]
			0.34 kWh	Ecoinvent v 3.8[17]
Output	Air emission	1-Butanol	$4.99*10^{-6}$ g	SimaPro airborne emission substance list[18]
		H_2SO_4	$4.55*10^{-11}$ g	SimaPro airborne emission substance list[19]
		H_2O	$6.56*10^{-7}$ g	SimaPro airborne emission substance list[20]
		Xylene	$7.71*10^{-6}$ g	SimaPro airborne emission substance list[21]
	End of life	Spent solvent mixture	113.27 g	Ecoinvent v 3.8[22]

[1]The Ecoinvnet process used was: Water, deionised {Europe without Switzerland}| water production, deionised | APOS, U.

[2]The Ecoinvent process used was: 1-butanol {RER}| hydroformylation of propylene | APOS, U.
[3]The Ecoinvent process used was: Sulfuric acid {RER}| production | APOS, U.
[4]The Ecoinvent process use was: Water, deionised {Europe without Switzerland}| water production, deionised | APOS, U.
[5]The Ecoinvent process used was: Sulfuric acid {RER}| production | APOS, U.
[6]The Ecoinvent process used was: Sodium hydroxide, without water, in 50% solution state {RER}| chlor-alkali electrolysis, mercury cell | APOS, U.
[7]The Ecoinvent process used was: Calcium chloride {RER}| soda production, solvay process | APOS, U.
[8]The Ecoinvent process used was: Xylene {RER}| production | APOS, U.
[9]Transport of large equipment (aspiration system and activated air carbon filter). The Ecoinvet process used was: Transport, freight, lorry 16-32 metric ton, EURO6 {RER}| transport, freight, lorry 16-32 metric ton, EURO6 | APOS, U. An average distance of 100km was considered.
[10]Transport for reagents. The Ecoinvent process used was: Transport, freight, lorry 3.5-7.5 metric ton, EURO6 {RER}| transport, freight, lorry 3.5-7.5 metric ton, EURO6 | APOS, U. An average distance of 100km was considered.
[11]Transport for small laboratory equipment. The Ecoinvent process used was: Transport, freight, lorry 3.5-7.5 metric ton, EURO6 {RER}| transport, freight, lorry 3.5-7.5 metric ton, EURO6 | APOS, U. An average distance of 100 km was considered.
[12]Electric energy necessary for the cooling down to 0°C of the reaction mixture containing NaBr, 1-buthanol and sulphuric acid. It is calculated using the expression *m*Cp*DT*, considering the specific heat in J/kg°C for each of the substances. The Ecoinvent process used was: Electricity, low voltage {IT}| electricity voltage transformation from medium to low voltage | APOS, U.
[13]Electric energy necessary for the heating up to for the reaction mixture to its boiling point, 200°C. It is calculated using the expression *m*Cp*DT*, considering the specific heat in J/kg°C for each of the substances. The Ecoinvent process used was: Electricity, low voltage {IT}| electricity voltage transformation from medium to low voltage | APOS, U.
[14]Electric energy necessary to maintain the heating at 200°C of the reaction mixture for 45 minutes. It is calculated using the Fourier Equation. The Ecoinvent process used was: Electricity, low voltage {IT}| electricity voltage transformation from medium to low voltage | APOS, U.
[15]Electric energy necessary for the first distillation process at 115°C in order to recover the final product, bromobutane, from the other co-products. It is calculated using the expression *m*Cp*DT*, considering the specific heat in J/kg°C for each of the substances. The Ecoinvent process used was: Electricity, low voltage {IT}| electricity voltage transformation from medium to low voltage | APOS, U.
[16]Electric energy necessary for the second distillation process at 102°C to purify the finale product, bromobutane, from impurities.It is calculated using the expression *m*Cp*DT*, considering the specific heat in J/kg°C for each of the substances. The Ecoinvent process used was: Electricity, low voltage {IT}| electricity voltage transformation from medium to low voltage | APOS, U.
[17]Electric energy necessary to the use of the aspiration system for the whole synthesis time, i.e. 4 h. The power was calculated by considering the air flow rate of 250 m3/h, a total load loss of 110.8076 kg/m2, and an efficiency of 90%: Electricity, low voltage {IT}| electricity voltage transformation from medium to low voltage | APOS, U.
[18]Amount of 1-Buthanol released into the atmosphere, as calculated by the formula reported in equation 1 of the main manuscript.
[19]Amount of Sulphuric acid released into the atmosphere, as calculated by the formula reported in equation 4 of the main manuscript.
[20]Amount of Water released into the atmosphere, as calculated by the formula reported in equation 4 of the main manuscript.
[21]Amount of Xylene released into the atmosphere, as calculated by the formula reported in equation 4 of the main manuscript.
[22]End of life of everything that is not the final product. The Ecoinvent process used was: Spent solvent mixture {Europe without Switzerland}| treatment of spent solvent mixture, hazardous waste incineration, with energy recovery | APOS, U.

Table S24. Contributions to the Life Cycle Inventory (LCI) for the synthesis of 0.53968 g of 4-butoxy-3-methoxybenzaldehyde. This is Reaction 1 (Run 11), carried in DMF as solvent with a reaction time of 8 hours, as reported in Table 1 in the main manuscript.

Description			Amount	Process Data Source
Input	Materials	DMF	4.72 g	Ecoinvent v 3.8[1]
		K_2CO_3	0.435 g	Ecoinvent v 3.8[2]
		KCl	1.022 g	Ecoinvent v 3.8[3]
		Bromobutane	0.562 g	Modelled from Ecoinvent v 3.8 database sub process as detailed in Table S23
		Vanillin	0.520 g	Modelled from Ecoinvent v 3.8 database sub process as detailed in Table S20
		H_2O	5 g	Ecoinvent v 3.8[4]
		Diethyl ether	21.405 g	Ecoinvent v 3.8[5]
		Mg_2SO_4	1 g	Ecoinvent v 3.8[6]
		Silicon oil	$1.72*10^{-2}$ p	Ecoinvent v 3.8[7]
	Equipment/ plants	Magnetic stirrer/heater	$1.8*10^{-4}$ p	Modelled from Ecoinvent v 3.8 database sub process as detailed in Table S18
		Round bottom flask	$3.47*10^{-4}$ p	Modelled from Ecoinvent v 3.8 database sub process as detailed in Table S19
		Separatory funnel	$2.33*10^{-5}$ p	Modelled from Ecoinvent v 3.8 database sub process as detailed in Table S9
		Reflux condenser	$3.12*10^{-4}$ p	Modelled from Ecoinvent v 3.8 database sub process as detailed in Table S13
		Silicon oil container	$3.47*10^{-4}$ p	Modelled from Ecoinvent v 3.8 database sub process as detailed in Table S6
		Funnel	$2.90*10^{-6}$ p	Modelled from Ecoinvent v 3.8 database sub process as detailed in Table S8
		Filter	$3.18*10^{-6}$ p	Modelled from Ecoinvent v 3.8 database

				sub process as detailed in Table S7
		Fitting reflux condenser	$3.12*10^{-4}$ p	Modelled from Ecoinvent v 3.8 database sub process as detailed in Table S12
		Rubber tube	$5.56*10^{-4}$ p	Modelled from Ecoinvent v 3.8 database sub process as detailed in Table S14
		Activated carbon air filter	$2*10^{-4}$ p	Modelled from Ecoinvent v 3.8 database sub process as detailed in Table S4
		Aspiration system	$5.02*10^{-5}$ p	Modelled from Ecoinvent v 3.8 database sub process as detailed in Table S3
	Transport	Transport for large equipment	$2.29*10^{-2}$ tkm	Ecoinvent v 3.8[8]
		Transport for raw materials	6.54 kgkm	Ecoinvent v 3.8[9]
		Transport for small equipment	$6.89*10^{-2}$ kgkm	Ecoinvent v 3.8[10]
	Energy	Electric energy	42.90 kJ	Ecoinvent v 3.8[11]
			1440 Wh	Ecoinvent v 3.8[12]
			3.015 kJ	Ecoinvent v 3.8[13]
			630 Wh	Ecoinvent v 3.8[14]
			500 Wh	Ecoinvent v 3.8[15]
			0.92 kWh	Ecoinvent v 3.8[16]
Output	Air emission	DMF	$7.28*10^{-7}$ g	SimaPro airborne emission substance list[17]
		HBr	$5.41*10^{-3}$ g	SimaPro airborne emission substance list[18]
		H_2O	$1.15*10^{-6}$ g	SimaPro airborne emission substance list[19]
		Diethyl ether	$5.291*10^{-4}$ g	SimaPro airborne emission substance list[20]
	End of life	Spent solvent mixture	31.125 g	Ecoinvent v 3.8[21]
		Incineration	2.457 g	Ecoinvent v 3.8[22]

[1]The Ecoinvnet process used was: N,N-dimethylformamide {RER}| production | APOS, U.

[2]The Ecoinvent process used was: Potassium carbonate {RER}| oxidation of manganese dioxide | APOS, U.
[3]The Ecoinvent process used was: Potassium chloride {RER}| potassium chloride production | APOS, U. During the experimental work potassium iodide was used. Since it is not present in the database, potassium chloride was used as proxy.
[4]The Ecoinvent process use was: Water, deionised {Europe without Switzerland}| water production, deionised | APOS, U.
[5]The Ecoinvent process used was: Diethyl ether, without water, in 99.95% solution state {RER}| ethylene hydration | APOS, U.
[6]The Ecoinvent process used was: Magnesium sulfate {RER}| production | APOS, U.
[7]The Ecoinvent process used was: Silicone product {RER}| production | APOS, U.
[8]Transport of large equipment (aspiration system and activated air carbon filter). The Ecoinvet process used was: Transport, freight, lorry 16-32 metric ton, EURO6 {RER}| transport, freight, lorry 16-32 metric ton, EURO6 | APOS, U. An average distance of 100km was considered.
[9]Transport for reagents. The Ecoinvent process used was: Transport, freight, lorry 3.5-7.5 metric ton, EURO6 {RER}| transport, freight, lorry 3.5-7.5 metric ton, EURO6 | APOS, U. An average distance of 100km was considered.
[10]Transport for small laboratory equipment. The Ecoinvent process used was: Transport, freight, lorry 3.5-7.5 metric ton, EURO6 {RER}| transport, freight, lorry 3.5-7.5 metric ton, EURO6 | APOS, U. An average distance of 100 km was considered.
[11]Electric energy necessary for the heating up to 100°C of the reaction mixture containing DMF, Vanillin and Bromobutane. It is calculated using the expression $m*Cp*DT$, considering the specific heat in J/kg°C for each of the substances. The Ecoinvent process used was: Electricity, low voltage {IT}| electricity voltage transformation from medium to low voltage | APOS, U.
[12]Electric energy necessary to maintain the heating at 100°C of the reaction mixture for 8 hours. It is calculated using the Fourier Equation. The Ecoinvent process used was: Electricity, low voltage {IT}| electricity voltage transformation from medium to low voltage | APOS, U.
[13]Electric energy necessary to eliminate the remaining solvent (DMF) using the rotavapor. It is calculated as HvapDMF*molDMF, considering HvapDMF as its latent heat of vaporization (46.7 kJ/mol) and molDMF as the moles of the solvent. The Ecoinvent process used was: Electricity, low voltage {IT}| electricity voltage transformation from medium to low voltage | APOS, U.
[14]Electric energy necessary to use the heating stirrer during the workup phase for 1 hour. It is calculated as power*time, considering 630W as the heating stirrer power. The Ecoinvent process used was: Electricity, low voltage {IT}| electricity voltage transformation from medium to low voltage | APOS, U.
[15]Electric energy necessary to use the rotavapor after the workup phase for 1 hour. It is calculated as power*time, considering 500W as the rotavapor power. The Ecoinvent process used was: Electricity, low voltage {IT}| electricity voltage transformation from medium to low voltage | APOS, U.
[16]Electric energy necessary to the use of the aspiration system for the whole synthesis time, i.e. 11 h. The power was calculated by considering the air flow rate of 250 m3/h, a total load loss of 110.8076 kg/m2, and an efficiency of 90%: Electricity, low voltage {IT}| electricity voltage transformation from medium to low voltage | APOS, U.
[17]Amount of DMF released into the atmosphere, as calculated by the formula reported in equation 1 of the main manuscript.
[18]Amount of HBr released into the atmosphere, as calculated by the formula reported in equation 4 of the main manuscript.
[19]Amount of Water released into the atmosphere, as calculated by the formula reported in equation 4 of the main manuscript.
[20]Amount of Diethyl ether released into the atmosphere, as calculated by the formula reported in equation 4 of the main manuscript.
[21]End of life of liquid waste, including DMF, Diethyl ether and water. The Ecoinvent process used was: Spent solvent mixture {Europe without Switzerland}| treatment of spent solvent mixture, hazardous waste incineration, with energy recovery | APOS, U.
[22]End of life of the solid waste recovered after the filtration following the first part of the synthesis. The Ecoinvent process used was: Hazardous waste, for incineration {RoW}| treatment of hazardous waste, hazardous waste incineration, with energy recovery | APOS, U.

Table S25. Contributions to the Life Cycle Inventory (LCI) for the synthesis of 0.53443 g of 4-butoxy-3-methoxybenzaldehyde. This is Reaction 2 (Run 7), carried in Acetonitrile as solvent with a reaction time of 24 hours, as reported in Table 1 in the main manuscript.

Description			Amount	Process Data Source
Input	Materials	ACN	15.66 g	Ecoinvent v 3.8[1]
		K_2CO_3	0.471 g	Ecoinvent v 3.8[2]
		KCl	1.058 g	Ecoinvent v 3.8[3]
		Bromobutane	0.908 g	Modelled from Ecoinvent v 3.8 database sub process as detailed in Table S23
		Vanillin	0.501 g	Modelled from Ecoinvent v 3.8 database sub process as detailed in Table S20
		H_2O	5 g	Ecoinvent v 3.8[4]
		Diethyl ether	21.405 g	Ecoinvent v 3.8[5]
		Mg_2SO_4	1 g	Ecoinvent v 3.8[6]
		Silicon oil	$1.72*10^{-2}$ p	Ecoinvent v 3.8[7]
	Equipment/ plants	Magnetic stirrer/heater	$5*10^{-4}$ p	Modelled from Ecoinvent v 3.8 database sub process as detailed in Table S18
		Round bottom flask	$9.03*10^{-4}$ p	Modelled from Ecoinvent v 3.8 database sub process as detailed in Table S15
		Separatory funnel	$2.33*10^{-5}$ p	Modelled from Ecoinvent v 3.8 database sub process as detailed in Table S9
		Reflux condenser	$8.69*10^{-4}$ p	Modelled from Ecoinvent v 3.8 database sub process as detailed in Table S13
		Silicon oil container	$9.03*10^{-4}$ p	Modelled from Ecoinvent v 3.8 database sub process as detailed in Table S6
		Funnel	$2.90*10^{-6}$ p	Modelled from Ecoinvent v 3.8 database sub process as detailed in Table S8
		Filter	$3.18*10^{-6}$ p	Modelled from Ecoinvent v 3.8 database

				sub process as detailed in Table S7
		Fitting reflux condenser	$8.69*10^{-4}$ p	Modelled from Ecoinvent v 3.8 database sub process as detailed in Table S12
		Rubber tube	$1.67*10^{-3}$ p	Modelled from Ecoinvent v 3.8 database sub process as detailed in Table S14
		Activated carbon air filter	$1.35*10^{-3}$ p	Modelled from Ecoinvent v 3.8 database sub process as detailed in Table S4
		Aspiration system	$3.38*10^{-4}$ p	Modelled from Ecoinvent v 3.8 database sub process as detailed in Table S3
	Transport	Transport for large equipment	$5.63*10^{-2}$ tkm	Ecoinvent v 3.8[8]
		Transport for raw materials	8.69 kgkm	Ecoinvent v 3.8[9]
		Transport for small equipment	$1.91*10^{-1}$ kgkm	Ecoinvent v 3.8[10]
	Energy	Electric energy	28.25 kJ	Ecoinvent v 3.8[11]
			3168 Wh	Ecoinvent v 3.8[12]
			11.37 kJ	Ecoinvent v 3.8[13]
			630 Wh	Ecoinvent v 3.8[14]
			500 Wh	Ecoinvent v 3.8[15]
			2.23 kWh	Ecoinvent v 3.8[16]
Output	Air emission	ACN	$4.28*10^{-5}$ g	SimaPro airborne emission substance list[17]
		HBr	$5.35*10^{-3}$ g	SimaPro airborne emission substance list[18]
		H_2O	$1.15*10^{-6}$ g	SimaPro airborne emission substance list[19]
		Diethyl ether	$5.291*10^{-4}$ g	SimaPro airborne emission substance list[20]
	End of life	Spent solvent mixture	42.065 g	Ecoinvent v 3.8[21]
		Incineration	2.529 g	Ecoinvent v 3.8[22]

[1]The Ecoinvnet process used was: Acetonitrile {RER}| Sohio process | APOS, U.

[2]The Ecoinvent process used was: Potassium carbonate {RER}| oxidation of manganese dioxide | APOS, U.
[3]The Ecoinvent process used was: Potassium chloride {RER}| potassium chloride production | APOS, U. During the experimental work potassium iodide was used. Since it is not present in the database, potassium chloride was used as proxy.
[4]The Ecoinvent process use was: Water, deionised {Europe without Switzerland}| water production, deionised | APOS, U.
[5]The Ecoinvent process used was: Diethyl ether, without water, in 99.95% solution state {RER}| ethylene hydration | APOS, U.
[6]The Ecoinvent process used was: Magnesium sulfate {RER}| production | APOS, U.
[7]The Ecoinvent process used was: Silicone product {RER}| production | APOS, U.
[8]Transport of large equipment (aspiration system and activated air carbon filter). The Ecoinvet process used was: Transport, freight, lorry 16-32 metric ton, EURO6 {RER}| transport, freight, lorry 16-32 metric ton, EURO6 | APOS, U. An average distance of 100km was considered.
[9]Transport for reagents. The Ecoinvent process used was: Transport, freight, lorry 3.5-7.5 metric ton, EURO6 {RER}| transport, freight, lorry 3.5-7.5 metric ton, EURO6 | APOS, U. An average distance of 100km was considered.
[10]Transport for small laboratory equipment. The Ecoinvent process used was: Transport, freight, lorry 3.5-7.5 metric ton, EURO6 {RER}| transport, freight, lorry 3.5-7.5 metric ton, EURO6 | APOS, U. An average distance of 100 km was considered.
[11]Electric energy necessary for the heating up to 100°C of the reaction mixture containing ACN, Vanillin and Bromobutane. It is calculated using the expression *m*Cp*DT*, considering the specific heat in J/kg°C for each of the substances. The Ecoinvent process used was: Electricity, low voltage {IT}| electricity voltage transformation from medium to low voltage | APOS, U.
[12]Electric energy necessary to maintain the heating at 100°C of the reaction mixture for 24 hours. It is calculated using the Fourier Equation. The Ecoinvent process used was: Electricity, low voltage {IT}| electricity voltage transformation from medium to low voltage | APOS, U.
[13]Electric energy necessary to eliminate the remaining solvent (ACN) using the rotavapor. It is calculated as HvapACN*molACN, considering HvapACN as its latent heat of vaporization (29.8 kJ/mol) and molACN as the moles of the solvent. The Ecoinvent process used was: Electricity, low voltage {IT}| electricity voltage transformation from medium to low voltage | APOS, U.
[14]Electric energy necessary to use the heating stirrer during the workup phase for 1 hour. It is calculated as power*time, considering 630W as the heating stirrer power. The Ecoinvent process used was: Electricity, low voltage {IT}| electricity voltage transformation from medium to low voltage | APOS, U.
[15]Electric energy necessary to use the rotavapor after the workup phase for 1 hour. It is calculated as power*time, considering 500W as the rotavapor power. The Ecoinvent process used was: Electricity, low voltage {IT}| electricity voltage transformation from medium to low voltage | APOS, U.
[16]Electric energy necessary to the use of the aspiration system for the whole synthesis time, i.e. 27 h. The power was calculated by considering the air flow rate of 250 m3/h, a total load loss of 110.8076 kg/m2, and an efficiency of 90%: Electricity, low voltage {IT}| electricity voltage transformation from medium to low voltage | APOS, U.
[17]Amount of ACN released into the atmosphere, as calculated by the formula reported in equation 1 of the main manuscript.
[18]Amount of HBr released into the atmosphere, as calculated by the formula reported in equation 4 of the main manuscript.
[19]Amount of Water released into the atmosphere, as calculated by the formula reported in equation 4 of the main manuscript.
[20]Amount of Diethyl ether released into the atmosphere, as calculated by the formula reported in equation 4 of the main manuscript.
[21]End of life of liquid waste, including ACN, Diethyl ether and water. The Ecoinvent process used was: Spent solvent mixture {Europe without Switzerland}| treatment of spent solvent mixture, hazardous waste incineration, with energy recovery | APOS, U.
[22]End of life of the solid waste recovered after the filtration following the first part of the synthesis. The Ecoinvent process used was: Hazardous waste, for incineration {RoW}| treatment of hazardous waste, hazardous waste incineration, with energy recovery | APOS, U.

Table S26. Contributions to the Life Cycle Inventory (LCI) for the synthesis of 0.534449 g of 4-butoxy-3-methoxybenzaldehyde. This is Reaction 3 (Run 3), carried in Acetone as solvent with a reaction time of 24 hours, as reported in Table 1 in the main manuscript.

Description			Amount	Process Data Source
Input	Materials	Acetone	15.68 g	Ecoinvent v 3.8[1]
		K_2CO_3	0.5185 g	Ecoinvent v 3.8[2]
		KCl	1.019 g	Ecoinvent v 3.8[3]
		Bromobutane	0.452 g	Modelled from Ecoinvent v 3.8 database sub process as detailed in Table S23
		Vanillin	0.503 g	Modelled from Ecoinvent v 3.8 database sub process as detailed in Table S20
		H_2O	5 g	Ecoinvent v 3.8[4]
		Diethyl ether	21.405 g	Ecoinvent v 3.8[5]
		Mg_2SO_4	1 g	Ecoinvent v 3.8[6]
		Silicon oil	$1.72*10^{-2}$ p	Ecoinvent v 3.8[7]
	Equipment/ plants	Magnetic stirrer/heater	$5*10^{-4}$ p	Modelled from Ecoinvent v 3.8 database sub process as detailed in Table S18
		Round bottom flask	$9.03*10^{-4}$ p	Modelled from Ecoinvent v 3.8 database sub process as detailed in Table S15
		Separatory funnel	$2.33*10^{-5}$ p	Modelled from Ecoinvent v 3.8 database sub process as detailed in Table S9
		Reflux condenser	$8.69*10^{-4}$ p	Modelled from Ecoinvent v 3.8 database sub process as detailed in Table S13
		Silicon oil container	$9.03*10^{-4}$ p	Modelled from Ecoinvent v 3.8 database sub process as detailed in Table S6
		Funnel	$2.90*10^{-6}$ p	Modelled from Ecoinvent v 3.8 database sub process as detailed in Table S8
		Filter	$3.18*10^{-6}$ p	Modelled from Ecoinvent v 3.8 database

				sub process as detailed in Table S7
		Fitting reflux condenser	8.69*10-4 p	Modelled from Ecoinvent v 3.8 database sub process as detailed in Table S12
		Rubber tube	1.67*10-3 p	Modelled from Ecoinvent v 3.8 database sub process as detailed in Table S14
		Activated carbon air filter	1.35*10-3 p	Modelled from Ecoinvent v 3.8 database sub process as detailed in Table S4
		Aspiration system	3.38*10-4 p	Modelled from Ecoinvent v 3.8 database sub process as detailed in Table S3
	Transport	Transport for large equipment	5.63*10-2 tkm	Ecoinvent v 3.8[8]
		Transport for raw materials	8.61 kgkm	Ecoinvent v 3.8[9]
		Transport for small equipment	1.91*10-1 kgkm	Ecoinvent v 3.8[10]
	Energy	Electric energy	11.40 kJ	Ecoinvent v 3.8[11]
			1440 Wh	Ecoinvent v 3.8[12]
			8.64 kJ	Ecoinvent v 3.8[13]
			630 Wh	Ecoinvent v 3.8[14]
			500 Wh	Ecoinvent v 3.8[15]
			2.26 kWh	Ecoinvent v 3.8[16]
Output	Air emission	Acetone	1.44*10-4 g	SimaPro airborne emission substance list[17]
		HBr	3.43*10-3 g	SimaPro airborne emission substance list[18]
		H_2O	1.15*10-6 g	SimaPro airborne emission substance list[19]
		Diethyl ether	5.29*10-4 g	SimaPro airborne emission substance list[20]
	End of life	Spent solvent mixture	42.085 g	Ecoinvent v 3.8[21]
		Incineration	2.538 g	Ecoinvent v 3.8[22]

[1]The Ecoinvnet process used was: Acetone, liquid {RER}| production | APOS, U

[2]The Ecoinvent process used was: Potassium carbonate {RER}| oxidation of manganese dioxide | APOS, U.
[3]The Ecoinvent process used was: Potassium chloride {RER}| potassium chloride production | APOS, U. During the experimental work potassium iodide was used. Since it is not present in the database, potassium chloride was used as proxy.
[4]The Ecoinvent process use was: Water, deionised {Europe without Switzerland}| water production, deionised | APOS, U.
[5]The Ecoinvent process used was: Diethyl ether, without water, in 99.95% solution state {RER}| ethylene hydration | APOS, U.
[6]The Ecoinvent process used was: Magnesium sulfate {RER}| production | APOS, U.
[7]The Ecoinvent process used was: Silicone product {RER}| production | APOS, U.
[8]Transport of large equipment (aspiration system and activated air carbon filter). The Ecoinvet process used was: Transport, freight, lorry 16-32 metric ton, EURO6 {RER}| transport, freight, lorry 16-32 metric ton, EURO6 | APOS, U. An average distance of 100km was considered.
[9]Transport for reagents. The Ecoinvent process used was: Transport, freight, lorry 3.5-7.5 metric ton, EURO6 {RER}| transport, freight, lorry 3.5-7.5 metric ton, EURO6 | APOS, U. An average distance of 100km was considered.
[10]Transport for small laboratory equipment. The Ecoinvent process used was: Transport, freight, lorry 3.5-7.5 metric ton, EURO6 {RER}| transport, freight, lorry 3.5-7.5 metric ton, EURO6 | APOS, U. An average distance of 100 km was considered.
[11]Electric energy necessary for the heating up to 100°C of the reaction mixture containing Acetone, Vanillin and Bromobutane. It is calculated using the expression *m*Cp*DT*, considering the specific heat in J/kg°C for each of the substances. The Ecoinvent process used was: Electricity, low voltage {IT}| electricity voltage transformation from medium to low voltage | APOS, U.
[12]Electric energy necessary to maintain the heating at 100°C of the reaction mixture for 24 hours. It is calculated using the Fourier Equation. The Ecoinvent process used was: Electricity, low voltage {IT}| electricity voltage transformation from medium to low voltage | APOS, U.
[13]Electric energy necessary to eliminate the remaining solvent (ACN) using the rotavapor. It is calculated as HvapAcetone*molAcetone, considering HvapAcentone as its latent heat of vaporization (32 kJ/mol) and molAcetone as the moles of the solvent. The Ecoinvent process used was: Electricity, low voltage {IT}| electricity voltage transformation from medium to low voltage | APOS, U.
[14]Electric energy necessary to use the heating stirrer during the workup phase for 1 hour. It is calculated as power*time, considering 630W as the heating stirrer power. The Ecoinvent process used was: Electricity, low voltage {IT}| electricity voltage transformation from medium to low voltage | APOS, U.
[15]Electric energy necessary to use the rotavapor after the workup phase for 1 hour. It is calculated as power*time, considering 500W as the rotavapor power. The Ecoinvent process used was: Electricity, low voltage {IT}| electricity voltage transformation from medium to low voltage | APOS, U.
[16]Electric energy necessary to the use of the aspiration system for the whole synthesis time, i.e. 27 h. The power was calculated by considering the air flow rate of 250 m3/h, a total load loss of 110.8076 kg/m2, and an efficiency of 90%: Electricity, low voltage {IT}| electricity voltage transformation from medium to low voltage | APOS, U.
[17]Amount of Acetone released into the atmosphere, as calculated by the formula reported in equation 4 of the main manuscript.
[18]Amount of HBr released into the atmosphere, as calculated by the formula reported in equation 4 of the main manuscript.
[19]Amount of Water released into the atmosphere, as calculated by the formula reported in equation 4 of the main manuscript.
[20]Amount of Diethyl ether released into the atmosphere, as calculated by the formula reported in equation 4 of the main manuscript.
[21]End of life of liquid waste, including Acetone, Diethyl ether and water. The Ecoinvent process used was: Spent solvent mixture {Europe without Switzerland}| treatment of spent solvent mixture, hazardous waste incineration, with energy recovery | APOS, U.
[22]End of life of the solid waste recovered after the filtration following the first part of the synthesis. The Ecoinvent process used was: Hazardous waste, for incineration {RoW}| treatment of hazardous waste, hazardous waste incineration, with energy recovery | APOS, U.

Table S27. Contributions to the Life Cycle Inventory (LCI) for the synthesis of 0.46741 g of 4-butoxy-3-methoxybenzaldehyde. This is Reaction 4 (Run 14), carried in DMF as solvent with a reaction time of 16 hours, as reported in Table 1 in the main manuscript.

Description			Amount	Process Data Source
Input	Materials	DMF	4.72 g	Ecoinvent v 3.8[1]
		K_2CO_3	0.692 g	Ecoinvent v 3.8[2]
		KCl	0.559 g	Ecoinvent v 3.8[3]
		Bromobutane	0.675 g	Modelled from Ecoinvent v 3.8 database sub process as detailed in Table S23
		Vanillin	0.508 g	Modelled from Ecoinvent v 3.8 database sub process as detailed in Table S20
		H_2O	5 g	Ecoinvent v 3.8[4]
		Diethyl ether	21.405 g	Ecoinvent v 3.8[5]
		Mg_2SO_4	1 g	Ecoinvent v 3.8[6]
		Silicon oil	$1.72*10^{-2}$ p	Ecoinvent v 3.8[7]
	Equipment/ plants	Magnetic stirrer/heater	$3.4*10^{-4}$ p	Modelled from Ecoinvent v 3.8 database sub process as detailed in Table S18
		Round bottom flask	$6.25*10^{-4}$ p	Modelled from Ecoinvent v 3.8 database sub process as detailed in Table S15
		Separatory funnel	$2.33*10^{-5}$ p	Modelled from Ecoinvent v 3.8 database sub process as detailed in Table S9
		Reflux condenser	$3.12*10^{-4}$ p	Modelled from Ecoinvent v 3.8 database sub process as detailed in Table S13
		Silicon oil container	$6.25*10^{-4}$ p	Modelled from Ecoinvent v 3.8 database sub process as detailed in Table S6
		Funnel	$2.90*10^{-6}$ p	Modelled from Ecoinvent v 3.8 database sub process as detailed in Table S8
		Filter	$3.18*10^{-6}$ p	Modelled from Ecoinvent v 3.8 database

				sub process as detailed in Table S7
		Fitting reflux condenser	$3.12*10^{-4}$ p	Modelled from Ecoinvent v 3.8 database sub process as detailed in Table S12
		Rubber tube	$5.56*10^{-4}$ p	Modelled from Ecoinvent v 3.8 database sub process as detailed in Table S14
		Activated carbon air filter	$9.5*10^{-4}$ p	Modelled from Ecoinvent v 3.8 database sub process as detailed in Table S4
		Aspiration system	$2.40*10^{-4}$ p	Modelled from Ecoinvent v 3.8 database sub process as detailed in Table S3
	Transport	Transport for large equipment	$2.29*10^{-2}$ tkm	Ecoinvent v 3.8[8]
		Transport for raw materials	6.54 kgkm	Ecoinvent v 3.8[9]
		Transport for small equipment	$6.89*10^{-2}$ kgkm	Ecoinvent v 3.8[10]
	Energy	Electric energy	42.90 kJ	Ecoinvent v 3.8[11]
			2880 Wh	Ecoinvent v 3.8[12]
			3.015 kJ	Ecoinvent v 3.8[13]
			630 Wh	Ecoinvent v 3.8[14]
			500 Wh	Ecoinvent v 3.8[15]
			1.59 kWh	Ecoinvent v 3.8[16]
Output	Air emission	DMF	$7.28*10^{-7}$ g	SimaPro airborne emission substance list[17]
		HBr	$5.08*10^{-3}$ g	SimaPro airborne emission substance list[18]
		H_2O	$1.15*10^{-6}$ g	SimaPro airborne emission substance list[19]
		Diethyl ether	$5.291*10^{-4}$ g	SimaPro airborne emission substance list[20]
	End of life	Spent solvent mixture	31.125 g	Ecoinvent v 3.8[21]
		Incineration	2.251 g	Ecoinvent v 3.8[22]

[1]The Ecoinvnet process used was: N,N-dimethylformamide {RER}| production | APOS, U.

[2]The Ecoinvent process used was: Potassium carbonate {RER}| oxidation of manganese dioxide | APOS, U.
[3]The Ecoinvent process used was: Potassium chloride {RER}| potassium chloride production | APOS, U. During the experimental work potassium iodide was used. Since it is not present in the database, potassium chloride was used as proxy.
[4]The Ecoinvent process use was: Water, deionised {Europe without Switzerland}| water production, deionised | APOS, U.
[5]The Ecoinvent process used was: Diethyl ether, without water, in 99.95% solution state {RER}| ethylene hydration | APOS, U.
[6]The Ecoinvent process used was: Magnesium sulfate {RER}| production | APOS, U.
[7]The Ecoinvent process used was: Silicone product {RER}| production | APOS, U.
[8]Transport of large equipment (aspiration system and activated air carbon filter). The Ecoinvet process used was: Transport, freight, lorry 16-32 metric ton, EURO6 {RER}| transport, freight, lorry 16-32 metric ton, EURO6 | APOS, U. An average distance of 100km was considered.
[9]Transport for reagents. The Ecoinvent process used was: Transport, freight, lorry 3.5-7.5 metric ton, EURO6 {RER}| transport, freight, lorry 3.5-7.5 metric ton, EURO6 | APOS, U. An average distance of 100km was considered.
[10]Transport for small laboratory equipment. The Ecoinvent process used was: Transport, freight, lorry 3.5-7.5 metric ton, EURO6 {RER}| transport, freight, lorry 3.5-7.5 metric ton, EURO6 | APOS, U. An average distance of 100 km was considered.
[11]Electric energy necessary for the heating up to 100°C of the reaction mixture containing DMF, Vanillin and Bromobutane. It is calculated using the expression *m*Cp*DT*, considering the specific heat in J/kg°C for each of the substances. The Ecoinvent process used was: Electricity, low voltage {IT}| electricity voltage transformation from medium to low voltage | APOS, U.
[12]Electric energy necessary to maintain the heating at 100°C of the reaction mixture for 16 hours. It is calculated using the Fourier Equation. The Ecoinvent process used was: Electricity, low voltage {IT}| electricity voltage transformation from medium to low voltage | APOS, U.
[13]Electric energy necessary to eliminate the remaining solvent (DMF) using the rotavapor. It is calculated as HvapDMF*molDMF, considering HvapDMF as its latent heat of vaporization (46.7 kJ/mol) and molDMF as the moles of the solvent. The Ecoinvent process used was: Electricity, low voltage {IT}| electricity voltage transformation from medium to low voltage | APOS, U.
[14]Electric energy necessary to use the heating stirrer during the workup phase for 1 hour. It is calculated as power*time, considering 630W as the heating stirrer power. The Ecoinvent process used was: Electricity, low voltage {IT}| electricity voltage transformation from medium to low voltage | APOS, U.
[15]Electric energy necessary to use the rotavapor after the workup phase for 1 hour. It is calculated as power*time, considering 500W as the rotavapor power. The Ecoinvent process used was: Electricity, low voltage {IT}| electricity voltage transformation from medium to low voltage | APOS, U.
[16]Electric energy necessary to the use of the aspiration system for the whole synthesis time, i.e. 19 h. The power was calculated by considering the air flow rate of 250 m3/h, a total load loss of 110.8076 kg/m2, and an efficiency of 90%: Electricity, low voltage {IT}| electricity voltage transformation from medium to low voltage | APOS, U.
[17]Amount of DMF released into the atmosphere, as calculated by the formula reported in equation 4 of the main manuscript.
[18]Amount of HBr released into the atmosphere, as calculated by the formula reported in equation 4 of the main manuscript.
[19]Amount of Water released into the atmosphere, as calculated by the formula reported in equation 4 of the main manuscript.
[20]Amount of Diethyl ether released into the atmosphere, as calculated by the formula reported in equation 4 of the main manuscript.
[21]End of life of liquid waste, including DMF, Diethyl ether and water. The Ecoinvent process used was: Spent solvent mixture {Europe without Switzerland}| treatment of spent solvent mixture, hazardous waste incineration, with energy recovery | APOS, U.
[22]End of life of the solid waste recovered after the filtration following the first part of the synthesis. The Ecoinvent process used was: Hazardous waste, for incineration {RoW}| treatment of hazardous waste, hazardous waste incineration, with energy recovery | APOS, U.

Table S28. Contributions to the Life Cycle Inventory (LCI) for the synthesis of 0.48177 g of 4-butoxy-3-methoxybenzaldehyde. This is Reaction 5 (Run 15), carried in DMF as solvent with a reaction time of 16 hours, as reported in Table 1 in the main manuscript.

Description			Amount	Process Data Source
Input	Materials	DMF	4.72 g	Ecoinvent v 3.8[1]
		K_2CO_3	0.692 g	Ecoinvent v 3.8[2]
		KCl	0.559 g	Ecoinvent v 3.8[3]
		Bromobutane	0.675 g	Modelled from Ecoinvent v 3.8 database sub process as detailed in Table S23
		Vanillin	0.508 g	Modelled from Ecoinvent v 3.8 database sub process as detailed in Table S20
		H_2O	5 g	Ecoinvent v 3.8[4]
		Diethyl ether	21.405 g	Ecoinvent v 3.8[5]
		Mg_2SO_4	1 g	Ecoinvent v 3.8[6]
		Silicon oil	$1.72*10^{-2}$ p	Ecoinvent v 3.8[7]
	Equipment/ plants	Magnetic stirrer/heater	$3.4*10^{-4}$ p	Modelled from Ecoinvent v 3.8 database sub process as detailed in Table S18
		Round bottom flask	$6.25*10^{-4}$ p	Modelled from Ecoinvent v 3.8 database sub process as detailed in Table S15
		Separatory funnel	$2.33*10^{-5}$ p	Modelled from Ecoinvent v 3.8 database sub process as detailed in Table S9
		Reflux condenser	$3.12*10^{-4}$ p	Modelled from Ecoinvent v 3.8 database sub process as detailed in Table S13
		Silicon oil container	$6.25*10^{-4}$ p	Modelled from Ecoinvent v 3.8 database sub process as detailed in Table S6
		Funnel	$2.90*10^{-6}$ p	Modelled from Ecoinvent v 3.8 database sub process as detailed in Table S8
		Filter	$3.18*10^{-6}$ p	Modelled from Ecoinvent v 3.8 database

				sub process as detailed in Table S7
		Fitting reflux condenser	$3.12*10^{-4}$ p	Modelled from Ecoinvent v 3.8 database sub process as detailed in Table S12
		Rubber tube	$5.56*10^{-4}$ p	Modelled from Ecoinvent v 3.8 database sub process as detailed in Table S14
		Activated carbon air filter	$9.5*10^{-4}$ p	Modelled from Ecoinvent v 3.8 database sub process as detailed in Table S4
		Aspiration system	$2.40*10^{-4}$ p	Modelled from Ecoinvent v 3.8 database sub process as detailed in Table S3
	Transport	Transport for large equipment	$2.29*10^{-2}$ tkm	Ecoinvent v 3.8[8]
		Transport for raw materials	6.54 kgkm	Ecoinvent v 3.8[9]
		Transport for small equipment	$1.18*10^{-1}$ kgkm	Ecoinvent v 3.8[10]
	Energy	Electric energy	42.90 kJ	Ecoinvent v 3.8[11]
			2880 Wh	Ecoinvent v 3.8[12]
			3.015 kJ	Ecoinvent v 3.8[13]
			630 Wh	Ecoinvent v 3.8[14]
			500 Wh	Ecoinvent v 3.8[15]
			1.59 kWh	Ecoinvent v 3.8[16]
Output	Air emission	DMF	$7.28*10^{-7}$ g	SimaPro airborne emission substance list[17]
		HBr	$5.08*10^{-3}$ g	SimaPro airborne emission substance list[18]
		H_2O	$1.15*10^{-6}$ g	SimaPro airborne emission substance list[19]
		Diethyl ether	$5.291*10^{-4}$ g	SimaPro airborne emission substance list[20]
	End of life	Spent solvent mixture	31.125 g	Ecoinvent v 3.8[21]
		Incineration	2.251 g	Ecoinvent v 3.8[22]

[1]The Ecoinvnet process used was: N,N-dimethylformamide {RER}| production | APOS, U.

[2]The Ecoinvent process used was: Potassium carbonate {RER}| oxidation of manganese dioxide | APOS, U.
[3]The Ecoinvent process used was: Potassium chloride {RER}| potassium chloride production | APOS, U. During the experimental work potassium iodide was used. Since it is not present in the database, potassium chloride was used as proxy.
[4]The Ecoinvent process use was: Water, deionised {Europe without Switzerland}| water production, deionised | APOS, U.
[5]The Ecoinvent process used was: Diethyl ether, without water, in 99.95% solution state {RER}| ethylene hydration | APOS, U.
[6]The Ecoinvent process used was: Magnesium sulfate {RER}| production | APOS, U.
[7]The Ecoinvent process used was: Silicone product {RER}| production | APOS, U.
[8]Transport of large equipment (aspiration system and activated air carbon filter). The Ecoinvet process used was: Transport, freight, lorry 16-32 metric ton, EURO6 {RER}| transport, freight, lorry 16-32 metric ton, EURO6 | APOS, U. An average distance of 100km was considered.
[9]Transport for reagents. The Ecoinvent process used was: Transport, freight, lorry 3.5-7.5 metric ton, EURO6 {RER}| transport, freight, lorry 3.5-7.5 metric ton, EURO6 | APOS, U. An average distance of 100km was considered.
[10]Transport for small laboratory equipment. The Ecoinvent process used was: Transport, freight, lorry 3.5-7.5 metric ton, EURO6 {RER}| transport, freight, lorry 3.5-7.5 metric ton, EURO6 | APOS, U. An average distance of 100 km was considered.
[11]Electric energy necessary for the heating up to 100°C of the reaction mixture containing DMF, Vanillin and Bromobutane. It is calculated using the expression *m*Cp*DT*, considering the specific heat in J/kg°C for each of the substances. The Ecoinvent process used was: Electricity, low voltage {IT}| electricity voltage transformation from medium to low voltage | APOS, U.
[12]Electric energy necessary to maintain the heating at 100°C of the reaction mixture for 16 hours. It is calculated using the Fourier Equation. The Ecoinvent process used was: Electricity, low voltage {IT}| electricity voltage transformation from medium to low voltage | APOS, U.
[13]Electric energy necessary to eliminate the remaining solvent (DMF) using the rotavapor. It is calculated as HvapDMF*molDMF, considering HvapDMF as its latent heat of vaporization (46.7 kJ/mol) and molDMF as the moles of the solvent. The Ecoinvent process used was: Electricity, low voltage {IT}| electricity voltage transformation from medium to low voltage | APOS, U.
[14]Electric energy necessary to use the heating stirrer during the workup phase for 1 hour. It is calculated as power*time, considering 630W as the heating stirrer power. The Ecoinvent process used was: Electricity, low voltage {IT}| electricity voltage transformation from medium to low voltage | APOS, U.
[15]Electric energy necessary to use the rotavapor after the workup phase for 1 hour. It is calculated as power*time, considering 500W as the rotavapor power. The Ecoinvent process used was: Electricity, low voltage {IT}| electricity voltage transformation from medium to low voltage | APOS, U.
[16]Electric energy necessary to the use of the aspiration system for the whole synthesis time, i.e. 19 h. The power was calculated by considering the air flow rate of 250 m3/h, a total load loss of 110.8076 kg/m2, and an efficiency of 90%: Electricity, low voltage {IT}| electricity voltage transformation from medium to low voltage | APOS, U.
[17]Amount of DMF released into the atmosphere, as calculated by the formula reported in equation 4 of the main manuscript.
[18]Amount of HBr released into the atmosphere, as calculated by the formula reported in equation 4 of the main manuscript.
[19]Amount of Water released into the atmosphere, as calculated by the formula reported in equation 4 of the main manuscript.
[20]Amount of Diethyl ether released into the atmosphere, as calculated by the formula reported in equation 4 of the main manuscript.
[21]End of life of liquid waste, including DMF, Diethyl ether and water. The Ecoinvent process used was: Spent solvent mixture {Europe without Switzerland}| treatment of spent solvent mixture, hazardous waste incineration, with energy recovery | APOS, U.
[22]End of life of the solid waste recovered after the filtration following the first part of the synthesis. The Ecoinvent process used was: Hazardous waste, for incineration {RoW}| treatment of hazardous waste, hazardous waste incineration, with energy recovery | APOS, U.

Table S29. Contributions to the Life Cycle Inventory (LCI) for the synthesis of 0.14067 g of 4-butoxy-3-methoxybenzaldehyde. This is Reaction 6 (Run 1), carried in Acetone as solvent with a reaction time of 8 hours, as reported in Table 1 in the main manuscript.

Description			Amount	Process Data Source
Input	Materials	Acetone	15.68 g	Ecoinvent v 3.8[1]
		K_2CO_3	0.459 g	Ecoinvent v 3.8[2]
		KCl	0 g	Ecoinvent v 3.8[3]
		Bromobutane	0.922 g	Modelled from Ecoinvent v 3.8 database sub process as detailed in Table S23
		Vanillin	0.5056 g	Modelled from Ecoinvent v 3.8 database sub process as detailed in Table S20
		H_2O	5 g	Ecoinvent v 3.8[4]
		Diethyl ether	21.405 g	Ecoinvent v 3.8[5]
		Mg_2SO_4	1 g	Ecoinvent v 3.8[6]
		Silicon oil	$1.72*10^{-2}$ p	Ecoinvent v 3.8[7]
	Equipment/ plants	Magnetic stirrer/heater	$1.8*10^{-4}$ p	Modelled from Ecoinvent v 3.8 database sub process as detailed in Table S18
		Round bottom flask	$3.82*10^{-4}$ p	Modelled from Ecoinvent v 3.8 database sub process as detailed in Table S15
		Separatory funnel	$2.33*10^{-5}$ p	Modelled from Ecoinvent v 3.8 database sub process as detailed in Table S9
		Reflux condenser	$3.12*10^{-4}$ p	Modelled from Ecoinvent v 3.8 database sub process as detailed in Table S13
		Silicon oil container	$3.82*10^{-4}$ p	Modelled from Ecoinvent v 3.8 database sub process as detailed in Table S6
		Funnel	$2.90*10^{-6}$ p	Modelled from Ecoinvent v 3.8 database sub process as detailed in Table S8
		Filter	$3.18*10^{-6}$ p	Modelled from Ecoinvent v 3.8 database

				sub process as detailed in Table S7
		Fitting reflux condenser	$8.69*10^{-4}$ p	Modelled from Ecoinvent v 3.8 database sub process as detailed in Table S12
		Rubber tube	$1.67*10^{-3}$ p	Modelled from Ecoinvent v 3.8 database sub process as detailed in Table S14
		Activated carbon air filter	$5.5*10^{-4}$ p	Modelled from Ecoinvent v 3.8 database sub process as detailed in Table S4
		Aspiration system	$1.38*10^{-4}$ p	Modelled from Ecoinvent v 3.8 database sub process as detailed in Table S3
	Transport	Transport for large equipment	$2.29*10^{-2}$ tkm	Ecoinvent v 3.8[8]
		Transport for raw materials	8.50 kgkm	Ecoinvent v 3.8[9]
		Transport for small equipment	$6.95*10^{-2}$ kgkm	Ecoinvent v 3.8[10]
	Energy	Electric energy	11.40 kJ	Ecoinvent v 3.8[11]
			480 Wh	Ecoinvent v 3.8[12]
			8.64 kJ	Ecoinvent v 3.8[13]
			630 Wh	Ecoinvent v 3.8[14]
			500 Wh	Ecoinvent v 3.8[15]
			0.92 kWh	Ecoinvent v 3.8[16]
Output	Air emission	Acetone	$1.44*10^{-4}$ g	SimaPro airborne emission substance list[17]
		HBr	$2.80*10^{-3}$ g	SimaPro airborne emission substance list[18]
		H_2O	$1.15*10^{-6}$ g	SimaPro airborne emission substance list[19]
		Diethyl ether	$5.29*10^{-4}$ g	SimaPro airborne emission substance list[20]
	End of life	Spent solvent mixture	42.085 g	Ecoinvent v 3.8[21]
		Incineration	1.459 g	Ecoinvent v 3.8[22]

[1]The Ecoinvnet process used was: Acetone, liquid {RER}| production | APOS, U

[2]The Ecoinvent process used was: Potassium carbonate {RER}| oxidation of manganese dioxide | APOS, U.
[3]The Ecoinvent process used was: Potassium chloride {RER}| potassium chloride production | APOS, U. During the experimental work potassium iodide was used. Since it is not present in the database, potassium chloride was used as proxy. In this specific run, this reagent wad not used, for this reason its quantity is zero in this table.
[4]The Ecoinvent process use was: Water, deionised {Europe without Switzerland}| water production, deionised | APOS, U.
[5]The Ecoinvent process used was: Diethyl ether, without water, in 99.95% solution state {RER}| ethylene hydration | APOS, U.
[6]The Ecoinvent process used was: Magnesium sulfate {RER}| production | APOS, U.
[7]The Ecoinvent process used was: Silicone product {RER}| production | APOS, U.
[8]Transport of large equipment (aspiration system and activated air carbon filter). The Ecoinvet process used was: Transport, freight, lorry 16-32 metric ton, EURO6 {RER}| transport, freight, lorry 16-32 metric ton, EURO6 | APOS, U. An average distance of 100km was considered.
[9]Transport for reagents. The Ecoinvent process used was: Transport, freight, lorry 3.5-7.5 metric ton, EURO6 {RER}| transport, freight, lorry 3.5-7.5 metric ton, EURO6 | APOS, U. An average distance of 100km was considered.
[10]Transport for small laboratory equipment. The Ecoinvent process used was: Transport, freight, lorry 3.5-7.5 metric ton, EURO6 {RER}| transport, freight, lorry 3.5-7.5 metric ton, EURO6 | APOS, U. An average distance of 100 km was considered.
[11]Electric energy necessary for the heating up to 100°C of the reaction mixture containing Acetone, Vanillin and Bromobutane. It is calculated using the expression *m*Cp*DT*, considering the specific heat in J/kg°C for each of the substances. The Ecoinvent process used was: Electricity, low voltage {IT}| electricity voltage transformation from medium to low voltage | APOS, U.
[12]Electric energy necessary to maintain the heating at 100°C of the reaction mixture for 8 hours. It is calculated using the Fourier Equation. The Ecoinvent process used was: Electricity, low voltage {IT}| electricity voltage transformation from medium to low voltage | APOS, U.
[13]Electric energy necessary to eliminate the remaining solvent (ACN) using the rotavapor. It is calculated as HvapAcetone*molAcetone, considering HvapAcentone as its latent heat of vaporization (32 kJ/mol) and molAcetone as the moles of the solvent. The Ecoinvent process used was: Electricity, low voltage {IT}| electricity voltage transformation from medium to low voltage | APOS, U.
[14]Electric energy necessary to use the heating stirrer during the workup phase for 1 hour. It is calculated as power*time, considering 630W as the heating stirrer power. The Ecoinvent process used was: Electricity, low voltage {IT}| electricity voltage transformation from medium to low voltage | APOS, U.
[15]Electric energy necessary to use the rotavapor after the workup phase for 1 hour. It is calculated as power*time, considering 500W as the rotavapor power. The Ecoinvent process used was: Electricity, low voltage {IT}| electricity voltage transformation from medium to low voltage | APOS, U.
[16]Electric energy necessary to the use of the aspiration system for the whole synthesis time, i.e. 11 h. The power was calculated by considering the air flow rate of 250 m3/h, a total load loss of 110.8076 kg/m2, and an efficiency of 90%: Electricity, low voltage {IT}| electricity voltage transformation from medium to low voltage | APOS, U.
[17]Amount of Acetone released into the atmosphere, as calculated by the formula reported in equation 4 of the main manuscript.
[18]Amount of HBr released into the atmosphere, as calculated by the formula reported in equation 4 of the main manuscript.
[19]Amount of Water released into the atmosphere, as calculated by the formula reported in equation 4 of the main manuscript.
[20]Amount of Diethyl ether released into the atmosphere, as calculated by the formula reported in equation 4 of the main manuscript.
[21]End of life of liquid waste, including Acetone, Diethyl ether and water. The Ecoinvent process used was: Spent solvent mixture {Europe without Switzerland}| treatment of spent solvent mixture, hazardous waste incineration, with energy recovery | APOS, U.
[22]End of life of the solid waste recovered after the filtration following the first part of the synthesis. The Ecoinvent process used was: Hazardous waste, for incineration {RoW}| treatment of hazardous waste, hazardous waste incineration, with energy recovery | APOS, U.

Table S30. Contributions to the Life Cycle Inventory (LCI) for the synthesis of 0.25348 g of 4-butoxy-3-methoxybenzaldehyde. This is Reaction 7 (Run 16), carried in Acetone as solvent with a reaction time of 16 hours, as reported in Table 1 in the main manuscript.

Description			Amount	Process Data Source
Input	Materials	Acetone	15.68 g	Ecoinvent v 3.8[1]
		K_2CO_3	0.729 g	Ecoinvent v 3.8[2]
		KCl	0.526 g	Ecoinvent v 3.8[3]
		Bromobutane	0.699 g	Modelled from Ecoinvent v 3.8 database sub process as detailed in Table S23
		Vanillin	0.504 g	Modelled from Ecoinvent v 3.8 database sub process as detailed in Table S20
		H_2O	5 g	Ecoinvent v 3.8[4]
		Diethyl ether	21.405 g	Ecoinvent v 3.8[5]
		Mg_2SO_4	1 g	Ecoinvent v 3.8[6]
		Silicon oil	$1.72*10^{-2}$ p	Ecoinvent v 3.8[7]
	Equipment/ plants	Magnetic stirrer/heater	$3.6*10^{-4}$ p	Modelled from Ecoinvent v 3.8 database sub process as detailed in Table S18
		Round bottom flask	$6.25*10^{-4}$ p	Modelled from Ecoinvent v 3.8 database sub process as detailed in Table S15
		Separatory funnel	$2.33*10^{-5}$ p	Modelled from Ecoinvent v 3.8 database sub process as detailed in Table S9
		Reflux condenser	$5.90*10^{-4}$ p	Modelled from Ecoinvent v 3.8 database sub process as detailed in Table S13
		Silicon oil container	$6.25*10^{-4}$ p	Modelled from Ecoinvent v 3.8 database sub process as detailed in Table S6
		Funnel	$2.90*10^{-6}$ p	Modelled from Ecoinvent v 3.8 database sub process as detailed in Table S8
		Filter	$3.18*10^{-6}$ p	Modelled from Ecoinvent v 3.8 database

				sub process as detailed in Table S7
		Fitting reflux condenser	$5.90*10^{-4}$ p	Modelled from Ecoinvent v 3.8 database sub process as detailed in Table S12
		Rubber tube	$1.11*10^{-3}$ p	Modelled from Ecoinvent v 3.8 database sub process as detailed in Table S14
		Activated carbon air filter	$9.5*10^{-4}$ p	Modelled from Ecoinvent v 3.8 database sub process as detailed in Table S4
		Aspiration system	$2.38*10^{-4}$ p	Modelled from Ecoinvent v 3.8 database sub process as detailed in Table S3
	Transport	Transport for large equipment	$3.96*10^{-2}$ tkm	Ecoinvent v 3.8[8]
		Transport for raw materials	8.61 kgkm	Ecoinvent v 3.8[9]
		Transport for small equipment	$1.36*10^{-1}$ kgkm	Ecoinvent v 3.8[10]
	Energy	Electric energy	11.40 kJ	Ecoinvent v 3.8[11]
			960 Wh	Ecoinvent v 3.8[12]
			8.64 kJ	Ecoinvent v 3.8[13]
			630 Wh	Ecoinvent v 3.8[14]
			500 Wh	Ecoinvent v 3.8[15]
			1.59 kWh	Ecoinvent v 3.8[16]
Output	Air emission	Acetone	$1.44*10^{-4}$ g	SimaPro airborne emission substance list[17]
		HBr	$4.04*10^{-3}$ g	SimaPro airborne emission substance list[18]
		H_2O	$1.15*10^{-6}$ g	SimaPro airborne emission substance list[19]
		Diethyl ether	$5.29*10^{-4}$ g	SimaPro airborne emission substance list[20]
	End of life	Spent solvent mixture	42.085 g	Ecoinvent v 3.8[21]
		Incineration	2.255 g	Ecoinvent v 3.8[22]

[1]The Ecoinvnet process used was: Acetone, liquid {RER}| production | APOS, U

[2]The Ecoinvent process used was: Potassium carbonate {RER}| oxidation of manganese dioxide | APOS, U.
[3]The Ecoinvent process used was: Potassium chloride {RER}| potassium chloride production | APOS, U. During the experimental work potassium iodide was used. Since it is not present in the database, potassium chloride was used as proxy.
[4]The Ecoinvent process use was: Water, deionised {Europe without Switzerland}| water production, deionised | APOS, U.
[5]The Ecoinvent process used was: Diethyl ether, without water, in 99.95% solution state {RER}| ethylene hydration | APOS, U.
[6]The Ecoinvent process used was: Magnesium sulfate {RER}| production | APOS, U.
[7]The Ecoinvent process used was: Silicone product {RER}| production | APOS, U.
[8]Transport of large equipment (aspiration system and activated air carbon filter). The Ecoinvet process used was: Transport, freight, lorry 16-32 metric ton, EURO6 {RER}| transport, freight, lorry 16-32 metric ton, EURO6 | APOS, U. An average distance of 100km was considered.
[9]Transport for reagents. The Ecoinvent process used was: Transport, freight, lorry 3.5-7.5 metric ton, EURO6 {RER}| transport, freight, lorry 3.5-7.5 metric ton, EURO6 | APOS, U. An average distance of 100km was considered.
[10]Transport for small laboratory equipment. The Ecoinvent process used was: Transport, freight, lorry 3.5-7.5 metric ton, EURO6 {RER}| transport, freight, lorry 3.5-7.5 metric ton, EURO6 | APOS, U. An average distance of 100 km was considered.
[11]Electric energy necessary for the heating up to 100°C of the reaction mixture containing Acetone, Vanillin and Bromobutane. It is calculated using the expression *m*Cp*DT*, considering the specific heat in J/kg°C for each of the substances. The Ecoinvent process used was: Electricity, low voltage {IT}| electricity voltage transformation from medium to low voltage | APOS, U.
[12]Electric energy necessary to maintain the heating at 100°C of the reaction mixture for 16 hours. It is calculated using the Fourier Equation. The Ecoinvent process used was: Electricity, low voltage {IT}| electricity voltage transformation from medium to low voltage | APOS, U.
[13]Electric energy necessary to eliminate the remaining solvent (Acetone) using the rotavapor. It is calculated as HvapAcetone*molAcetone, considering HvapAcentone as its latent heat of vaporization (32 kJ/mol) and molAcetone as the moles of the solvent. The Ecoinvent process used was: Electricity, low voltage {IT}| electricity voltage transformation from medium to low voltage | APOS, U.
[14]Electric energy necessary to use the heating stirrer during the workup phase for 1 hour. It is calculated as power*time, considering 630W as the heating stirrer power. The Ecoinvent process used was: Electricity, low voltage {IT}| electricity voltage transformation from medium to low voltage | APOS, U.
[15]Electric energy necessary to use the rotavapor after the workup phase for 1 hour. It is calculated as power*time, considering 500W as the rotavapor power. The Ecoinvent process used was: Electricity, low voltage {IT}| electricity voltage transformation from medium to low voltage | APOS, U.
[16]Electric energy necessary to the use of the aspiration system for the whole synthesis time, i.e. 19 h. The power was calculated by considering the air flow rate of 250 m3/h, a total load loss of 110.8076 kg/m2, and an efficiency of 90%: Electricity, low voltage {IT}| electricity voltage transformation from medium to low voltage | APOS, U.
[17]Amount of Acetone released into the atmosphere, as calculated by the formula reported in equation 4 of the main manuscript.
[18]Amount of HBr released into the atmosphere, as calculated by the formula reported in equation 4 of the main manuscript.
[19]Amount of Water released into the atmosphere, as calculated by the formula reported in equation 4 of the main manuscript.
[20]Amount of Diethyl ether released into the atmosphere, as calculated by the formula reported in equation 4 of the main manuscript.
[21]End of life of liquid waste, including Acetone, Diethyl ether and water. The Ecoinvent process used was: Spent solvent mixture {Europe without Switzerland}| treatment of spent solvent mixture, hazardous waste incineration, with energy recovery | APOS, U.
[22]End of life of the solid waste recovered after the filtration following the first part of the synthesis. The Ecoinvent process used was: Hazardous waste, for incineration {RoW}| treatment of hazardous waste, hazardous waste incineration, with energy recovery | APOS, U.

Table S31. Contributions to the Life Cycle Inventory (LCI) for the synthesis of 0.23202 g of 4-butoxy-3-methoxybenzaldehyde. This is Reaction 8 (Run 17), carried in Acetone as solvent with a reaction time of 16 hours, as reported in Table 1 in the main manuscript.

Description			Amount	Process Data Source
Input	Materials	Acetone	15.68 g	Ecoinvent v 3.8[1]
		K_2CO_3	0.744 g	Ecoinvent v 3.8[2]
		KCl	0.556 g	Ecoinvent v 3.8[3]
		Bromobutane	0.693 g	Modelled from Ecoinvent v 3.8 database sub process as detailed in Table S23
		Vanillin	0.509 g	Modelled from Ecoinvent v 3.8 database sub process as detailed in Table S20
		H_2O	5 g	Ecoinvent v 3.8[4]
		Diethyl ether	21.405 g	Ecoinvent v 3.8[5]
		Mg_2SO_4	1 g	Ecoinvent v 3.8[6]
		Silicon oil	$1.72*10^{-2}$ p	Ecoinvent v 3.8[7]
	Equipment/ plants	Magnetic stirrer/heater	$3.6*10^{-4}$ p	Modelled from Ecoinvent v 3.8 database sub process as detailed in Table S18
		Round bottom flask	$6.25*10^{-4}$ p	Modelled from Ecoinvent v 3.8 database sub process as detailed in Table S15
		Separatory funnel	$2.33*10^{-5}$ p	Modelled from Ecoinvent v 3.8 database sub process as detailed in Table S9
		Reflux condenser	$5.90*10^{-4}$ p	Modelled from Ecoinvent v 3.8 database sub process as detailed in Table S13
		Silicon oil container	$6.25*10^{-4}$ p	Modelled from Ecoinvent v 3.8 database sub process as detailed in Table S6
		Funnel	$2.90*10^{-6}$ p	Modelled from Ecoinvent v 3.8 database sub process as detailed in Table S8

		Filter	$3.18*10^{-6}$ p	Modelled from Ecoinvent v 3.8 database sub process as detailed in Table S7
		Fitting reflux condenser	$5.90*10^{-4}$ p	Modelled from Ecoinvent v 3.8 database sub process as detailed in Table S12
		Rubber tube	$1.11*10^{-3}$ p	Modelled from Ecoinvent v 3.8 database sub process as detailed in Table S14
		Activated carbon air filter	$9.5*10^{-4}$ p	Modelled from Ecoinvent v 3.8 database sub process as detailed in Table S4
		Aspiration system	$2.38*10^{-4}$ p	Modelled from Ecoinvent v 3.8 database sub process as detailed in Table S3
	Transport	Transport for large equipment	$3.96*10^{-2}$ tkm	Ecoinvent v 3.8[8]
		Transport for raw materials	8.61 kgkm	Ecoinvent v 3.8[9]
		Transport for small equipment	$1.36*10^{-1}$ kgkm	Ecoinvent v 3.8[10]
	Energy	Electric energy	11.40 kJ	Ecoinvent v 3.8[11]
			960 Wh	Ecoinvent v 3.8[12]
			8.64 kJ	Ecoinvent v 3.8[13]
			630 Wh	Ecoinvent v 3.8[14]
			500 Wh	Ecoinvent v 3.8[15]
			1.59 kWh	Ecoinvent v 3.8[16]
Output	Air emission	Acetone	$1.44*10^{-4}$ g	SimaPro airborne emission substance list[17]
		HBr	$3.58*10^{-3}$ g	SimaPro airborne emission substance list[18]
		H_2O	$1.15*10^{-6}$ g	SimaPro airborne emission substance list[19]
		Diethyl ether	$5.29*10^{-4}$ g	SimaPro airborne emission substance list[20]
	End of life	Spent solvent mixture	42.085 g	Ecoinvent v 3.8[21]

	Incineration	2.300 g	Ecoinvent v 3.8[22]

[1]The Ecoinvnet process used was: Acetone, liquid {RER}| production | APOS, U

[2]The Ecoinvent process used was: Potassium carbonate {RER}| oxidation of manganese dioxide | APOS, U.

[3]The Ecoinvent process used was: Potassium chloride {RER}| potassium chloride production | APOS, U. During the experimental work potassium iodide was used. Since it is not present in the database, potassium chloride was used as proxy.

[4]The Ecoinvent process use was: Water, deionised {Europe without Switzerland}| water production, deionised | APOS, U.

[5]The Ecoinvent process used was: Diethyl ether, without water, in 99.95% solution state {RER}| ethylene hydration | APOS, U.

[6]The Ecoinvent process used was: Magnesium sulfate {RER}| production | APOS, U.

[7]The Ecoinvent process used was: Silicone product {RER}| production | APOS, U.

[8]Transport of large equipment (aspiration system and activated air carbon filter). The Ecoinvet process used was: Transport, freight, lorry 16-32 metric ton, EURO6 {RER}| transport, freight, lorry 16-32 metric ton, EURO6 | APOS, U. An average distance of 100km was considered.

[9]Transport for reagents. The Ecoinvent process used was: Transport, freight, lorry 3.5-7.5 metric ton, EURO6 {RER}| transport, freight, lorry 3.5-7.5 metric ton, EURO6 | APOS, U. An average distance of 100km was considered.

[10]Transport for small laboratory equipment. The Ecoinvent process used was: Transport, freight, lorry 3.5-7.5 metric ton, EURO6 {RER}| transport, freight, lorry 3.5-7.5 metric ton, EURO6 | APOS, U. An average distance of 100 km was considered.

[11]Electric energy necessary for the heating up to 100°C of the reaction mixture containing Acetone, Vanillin and Bromobutane. It is calculated using the expression *m*Cp*DT*, considering the specific heat in J/kg°C for each of the substances. The Ecoinvent process used was: Electricity, low voltage {IT}| electricity voltage transformation from medium to low voltage | APOS, U.

[12]Electric energy necessary to maintain the heating at 100°C of the reaction mixture for 16 hours. It is calculated using the Fourier Equation. The Ecoinvent process used was: Electricity, low voltage {IT}| electricity voltage transformation from medium to low voltage | APOS, U.

[13]Electric energy necessary to eliminate the remaining solvent (Acetone) using the rotavapor. It is calculated as HvapAcetone*molAcetone, considering HvapAcentone as its latent heat of vaporization (32 kJ/mol) and molAcetone as the moles of the solvent. The Ecoinvent process used was: Electricity, low voltage {IT}| electricity voltage transformation from medium to low voltage | APOS, U.

[14]Electric energy necessary to use the heating stirrer during the workup phase for 1 hour. It is calculated as power*time, considering 630W as the heating stirrer power. The Ecoinvent process used was: Electricity, low voltage {IT}| electricity voltage transformation from medium to low voltage | APOS, U.

[15]Electric energy necessary to use the rotavapor after the workup phase for 1 hour. It is calculated as power*time, considering 500W as the rotavapor power. The Ecoinvent process used was: Electricity, low voltage {IT}| electricity voltage transformation from medium to low voltage | APOS, U.

[16]Electric energy necessary to the use of the aspiration system for the whole synthesis time, i.e. 19 h. The power was calculated by considering the air flow rate of 250 m3/h, a total load loss of 110.8076 kg/m2, and an efficiency of 90%: Electricity, low voltage {IT}| electricity voltage transformation from medium to low voltage | APOS, U.

[17]Amount of Acetone released into the atmosphere, as calculated by the formula reported in equation 4 of the main manuscript.

[18]Amount of HBr released into the atmosphere, as calculated by the formula reported in equation 4 of the main manuscript.

[19]Amount of Water released into the atmosphere, as calculated by the formula reported in equation 4 of the main manuscript.

[20]Amount of Diethyl ether released into the atmosphere, as calculated by the formula reported in equation 4 of the main manuscript.

[21]End of life of liquid waste, including Acetone, Diethyl ether and water. The Ecoinvent process used was: Spent solvent mixture {Europe without Switzerland}| treatment of spent solvent mixture, hazardous waste incineration, with energy recovery | APOS, U.

[22]End of life of the solid waste recovered after the filtration following the first part of the synthesis. The Ecoinvent process used was: Hazardous waste, for incineration {RoW}| treatment of hazardous waste, hazardous waste incineration, with energy recovery | APOS, U.

Table S32. Contributions to the Life Cycle Inventory (LCI) for the synthesis of 0.34083 g of 4-butoxy-3-methoxybenzaldehyde. This is Reaction 9 (Run 12), carried in DMF as solvent with a reaction time of 24 hours, as reported in Table 1 in the main manuscript.

Description			Amount	Process Data Source
Input	Materials	DMF	4.72 g	Ecoinvent v 3.8[1]
		K_2CO_3	0.920 g	Ecoinvent v 3.8[2]
		KCl	0 g	Ecoinvent v 3.8[3]
		Bromobutane	0.931 g	Modelled from Ecoinvent v 3.8 database sub process as detailed in Table S23
		Vanillin	0.516 g	Modelled from Ecoinvent v 3.8 database sub process as detailed in Table S20
		H_2O	5 g	Ecoinvent v 3.8[4]
		Diethyl ether	21.405 g	Ecoinvent v 3.8[5]
		Mg_2SO_4	1 g	Ecoinvent v 3.8[6]
		Silicon oil	$1.72*10^{-2}$ p	Ecoinvent v 3.8[7]
	Equipment/ plants	Magnetic stirrer/heater	$1.8*10^{-4}$ p	Modelled from Ecoinvent v 3.8 database sub process as detailed in Table S18
		Round bottom flask	$3.47*10^{-4}$ p	Modelled from Ecoinvent v 3.8 database sub process as detailed in Table S15
		Separatory funnel	$2.33*10^{-5}$ p	Modelled from Ecoinvent v 3.8 database sub process as detailed in Table S9
		Reflux condenser	$3.12*10^{-4}$ p	Modelled from Ecoinvent v 3.8 database sub process as detailed in Table S13
		Silicon oil container	$3.47*10^{-4}$ p	Modelled from Ecoinvent v 3.8 database sub process as detailed in Table S6
		Funnel	$2.90*10^{-6}$ p	Modelled from Ecoinvent v 3.8 database sub process as detailed in Table S8

		Filter	$3.18*10^{-6}$ p	Modelled from Ecoinvent v 3.8 database sub process as detailed in Table S7
		Fitting reflux condenser	$3.12*10^{-4}$ p	Modelled from Ecoinvent v 3.8 database sub process as detailed in Table S12
		Rubber tube	$5.56*10^{-4}$ p	Modelled from Ecoinvent v 3.8 database sub process as detailed in Table S14
		Activated carbon air filter	$5.5*10^{-4}$ p	Modelled from Ecoinvent v 3.8 database sub process as detailed in Table S4
		Aspiration system	$1.38*10^{-4}$ p	Modelled from Ecoinvent v 3.8 database sub process as detailed in Table S3
	Transport	Transport for large equipment	$2.29*10^{-2}$ tkm	Ecoinvent v 3.8[8]
		Transport for raw materials	6.52 kgkm	Ecoinvent v 3.8[9]
		Transport for small equipment	$6.89*10^{-2}$ kgkm	Ecoinvent v 3.8[10]
	Energy	Electric energy	42.90 kJ	Ecoinvent v 3.8[11]
			1440 Wh	Ecoinvent v 3.8[12]
			3.015 kJ	Ecoinvent v 3.8[13]
			630 Wh	Ecoinvent v 3.8[14]
			500 Wh	Ecoinvent v 3.8[15]
			0.92 kWh	Ecoinvent v 3.8[16]
Output	Air emission	DMF	$7.28*10^{-7}$ g	SimaPro airborne emission substance list[17]
		HBr	$3.6*10^{-3}$ g	SimaPro airborne emission substance list[18]
		H_2O	$1.15*10^{-6}$ g	SimaPro airborne emission substance list[19]
		Diethyl ether	$5.291*10^{-4}$ g	SimaPro airborne emission substance list[20]
	End of life	Spent solvent mixture	31.125 g	Ecoinvent v 3.8[21]

	Incineration	1.920 g	Ecoinvent v 3.8[22]

[1]The Ecoinvnet process used was: N,N-dimethylformamide {RER}| production | APOS, U.

[2]The Ecoinvent process used was: Potassium carbonate {RER}| oxidation of manganese dioxide | APOS, U.

[3]The Ecoinvent process used was: Potassium chloride {RER}| potassium chloride production | APOS, U. During the experimental work potassium iodide was used. Since it is not present in the database, potassium chloride was used as proxy. In this specific run, this reagent wad not used, for this reason its quantity is zero in this table.

[4]The Ecoinvent process use was: Water, deionised {Europe without Switzerland}| water production, deionised | APOS, U.

[5]The Ecoinvent process used was: Diethyl ether, without water, in 99.95% solution state {RER}| ethylene hydration | APOS, U.

[6]The Ecoinvent process used was: Magnesium sulfate {RER}| production | APOS, U.

[7]The Ecoinvent process used was: Silicone product {RER}| production | APOS, U.

[8]Transport of large equipment (aspiration system and activated air carbon filter). The Ecoinvet process used was: Transport, freight, lorry 16-32 metric ton, EURO6 {RER}| transport, freight, lorry 16-32 metric ton, EURO6 | APOS, U. An average distance of 100km was considered.

[9]Transport for reagents. The Ecoinvent process used was: Transport, freight, lorry 3.5-7.5 metric ton, EURO6 {RER}| transport, freight, lorry 3.5-7.5 metric ton, EURO6 | APOS, U. An average distance of 100km was considered.

[10]Transport for small laboratory equipment. The Ecoinvent process used was: Transport, freight, lorry 3.5-7.5 metric ton, EURO6 {RER}| transport, freight, lorry 3.5-7.5 metric ton, EURO6 | APOS, U. An average distance of 100 km was considered.

[11]Electric energy necessary for the heating up to 100°C of the reaction mixture containing DMF, Vanillin and Bromobutane. It is calculated using the expression *m*Cp*DT*, considering the specific heat in J/kg°C for each of the substances. The Ecoinvent process used was: Electricity, low voltage {IT}| electricity voltage transformation from medium to low voltage | APOS, U.

[12]Electric energy necessary to maintain the heating at 100°C of the reaction mixture for 24 hours. It is calculated using the Fourier Equation. The Ecoinvent process used was: Electricity, low voltage {IT}| electricity voltage transformation from medium to low voltage | APOS, U.

[13]Electric energy necessary to eliminate the remaining solvent (DMF) using the rotavapor. It is calculated as HvapDMF*molDMF, considering HvapDMF as its latent heat of vaporization (46.7 kJ/mol) and molDMF as the moles of the solvent. The Ecoinvent process used was: Electricity, low voltage {IT}| electricity voltage transformation from medium to low voltage | APOS, U.

[14]Electric energy necessary to use the heating stirrer during the workup phase for 1 hour. It is calculated as power*time, considering 630W as the heating stirrer power. The Ecoinvent process used was: Electricity, low voltage {IT}| electricity voltage transformation from medium to low voltage | APOS, U.

[15]Electric energy necessary to use the rotavapor after the workup phase for 1 hour. It is calculated as power*time, considering 500W as the rotavapor power. The Ecoinvent process used was: Electricity, low voltage {IT}| electricity voltage transformation from medium to low voltage | APOS, U.

[16]Electric energy necessary to the use of the aspiration system for the whole synthesis time, i.e. 27 h. The power was calculated by considering the air flow rate of 250 m3/h, a total load loss of 110.8076 kg/m2, and an efficiency of 90%: Electricity, low voltage {IT}| electricity voltage transformation from medium to low voltage | APOS, U.

[17]Amount of DMF released into the atmosphere, as calculated by the formula reported in equation 4 of the main manuscript.

[18]Amount of HBr released into the atmosphere, as calculated by the formula reported in equation 4 of the main manuscript.

[19]Amount of Water released into the atmosphere, as calculated by the formula reported in equation 4 of the main manuscript.

[20]Amount of Diethyl ether released into the atmosphere, as calculated by the formula reported in equation 4 of the main manuscript.

[21]End of life of liquid waste, including DMF, Diethyl ether and water. The Ecoinvent process used was: Spent solvent mixture {Europe without Switzerland}| treatment of spent solvent mixture, hazardous waste incineration, with energy recovery | APOS, U.

[22]End of life of the solid waste recovered after the filtration following the first part of the synthesis. The Ecoinvent process used was: Hazardous waste, for incineration {RoW}| treatment of hazardous waste, hazardous waste incineration, with energy recovery | APOS, U.

Table S33. Contributions to the Life Cycle Inventory (LCI) for the synthesis of 0.0151 g of 4-butoxy-3-methoxybenzaldehyde. This is Reaction 10 (Run 4), carried in Acetone as solvent with a reaction time of 8 hours, as reported in Table 1 in the main manuscript.

Description			Amount	Process Data Source
Input	Materials	Acetone	15.68 g	Ecoinvent v 3.8[1]
		K_2CO_3	0.91169 g	Ecoinvent v 3.8[2]
		KCl	0 g	Ecoinvent v 3.8[3]
		Bromobutane	0.476 g	Modelled from Ecoinvent v 3.8 database sub process as detailed in Table S23
		Vanillin	0.519 g	Modelled from Ecoinvent v 3.8 database sub process as detailed in Table S20
		H_2O	5 g	Ecoinvent v 3.8[4]
		Diethyl ether	21.405 g	Ecoinvent v 3.8[5]
		Mg_2SO_4	1 g	Ecoinvent v 3.8[6]
		Silicon oil	$1.72*10^{-2}$ p	Ecoinvent v 3.8[7]
	Equipment/ plants	Magnetic stirrer/heater	$1.8*10^{-4}$ p	Modelled from Ecoinvent v 3.8 database sub process as detailed in Table S18
		Round bottom flask	$3.47*10^{-4}$ p	Modelled from Ecoinvent v 3.8 database sub process as detailed in Table S15
		Separatory funnel	$2.33*10^{-5}$ p	Modelled from Ecoinvent v 3.8 database sub process as detailed in Table S9
		Reflux condenser	$3.12*10^{-4}$ p	Modelled from Ecoinvent v 3.8 database sub process as detailed in Table S13
		Silicon oil container	$3.47*10^{-4}$ p	Modelled from Ecoinvent v 3.8 database sub process as detailed in Table S6
		Funnel	$2.90*10^{-6}$ p	Modelled from Ecoinvent v 3.8 database sub process as detailed in Table S8

		Filter	$3.18*10^{-6}$ p	Modelled from Ecoinvent v 3.8 database sub process as detailed in Table S7
		Fitting reflux condenser	$3.12*10^{-4}$ p	Modelled from Ecoinvent v 3.8 database sub process as detailed in Table S12
		Rubber tube	$5.56*10^{-4}$ p	Modelled from Ecoinvent v 3.8 database sub process as detailed in Table S14
		Activated carbon air filter	$5.5*10^{-4}$ p	Modelled from Ecoinvent v 3.8 database sub process as detailed in Table S4
		Aspiration system	$1.38*10^{-4}$ p	Modelled from Ecoinvent v 3.8 database sub process as detailed in Table S3
	Transport	Transport for large equipment	$2.29*10^{-2}$ tkm	Ecoinvent v 3.8[8]
		Transport for raw materials	8.50 kgkm	Ecoinvent v 3.8[9]
		Transport for small equipment	$5.36*10^{-1}$ kgkm	Ecoinvent v 3.8[10]
	Energy	Electric energy	11.40 kJ	Ecoinvent v 3.8[11]
			1440 Wh	Ecoinvent v 3.8[12]
			8.64 kJ	Ecoinvent v 3.8[13]
			630 Wh	Ecoinvent v 3.8[14]
			500 Wh	Ecoinvent v 3.8[15]
			0.92 kWh	Ecoinvent v 3.8[16]
Output	Air emission	Acetone	$1.44*10^{-4}$ g	SimaPro airborne emission substance list[17]
		HBr	$1.45*10^{-4}$ g	SimaPro airborne emission substance list[18]
		H_2O	$1.15*10^{-6}$ g	SimaPro airborne emission substance list[19]
		Diethyl ether	$5.29*10^{-4}$ g	SimaPro airborne emission substance list[20]
	End of life	Spent solvent mixture	42.085 g	Ecoinvent v 3.8[21]

	Incineration	1.912 g	Ecoinvent v 3.8[22]

[1]The Ecoinvnet process used was: Acetone, liquid {RER}| production | APOS, U

[2]The Ecoinvent process used was: Potassium carbonate {RER}| oxidation of manganese dioxide | APOS, U.

[3]The Ecoinvent process used was: Potassium chloride {RER}| potassium chloride production | APOS, U. During the experimental work potassium iodide was used. Since it is not present in the database, potassium chloride was used as proxy. In this specific run, this reagent wad not used, for this reason its quantity is zero in this table.

[4]The Ecoinvent process use was: Water, deionised {Europe without Switzerland}| water production, deionised | APOS, U.

[5]The Ecoinvent process used was: Diethyl ether, without water, in 99.95% solution state {RER}| ethylene hydration | APOS, U.

[6]The Ecoinvent process used was: Magnesium sulfate {RER}| production | APOS, U.

[7]The Ecoinvent process used was: Silicone product {RER}| production | APOS, U.

[8]Transport of large equipment (aspiration system and activated air carbon filter). The Ecoinvet process used was: Transport, freight, lorry 16-32 metric ton, EURO6 {RER}| transport, freight, lorry 16-32 metric ton, EURO6 | APOS, U. An average distance of 100km was considered.

[9]Transport for reagents. The Ecoinvent process used was: Transport, freight, lorry 3.5-7.5 metric ton, EURO6 {RER}| transport, freight, lorry 3.5-7.5 metric ton, EURO6 | APOS, U. An average distance of 100km was considered.

[10]Transport for small laboratory equipment. The Ecoinvent process used was: Transport, freight, lorry 3.5-7.5 metric ton, EURO6 {RER}| transport, freight, lorry 3.5-7.5 metric ton, EURO6 | APOS, U. An average distance of 100 km was considered.

[11]Electric energy necessary for the heating up to 100°C of the reaction mixture containing Acetone, Vanillin and Bromobutane. It is calculated using the expression *m*Cp*DT*, considering the specific heat in J/kg°C for each of the substances. The Ecoinvent process used was: Electricity, low voltage {IT}| electricity voltage transformation from medium to low voltage | APOS, U.

[12]Electric energy necessary to maintain the heating at 100°C of the reaction mixture for 8 hours. It is calculated using the Fourier Equation. The Ecoinvent process used was: Electricity, low voltage {IT}| electricity voltage transformation from medium to low voltage | APOS, U.

[13]Electric energy necessary to eliminate the remaining solvent (ACN) using the rotavapor. It is calculated as HvapAcetone*molAcetone, considering HvapAcentone as its latent heat of vaporization (32 kJ/mol) and molAcetone as the moles of the solvent. The Ecoinvent process used was: Electricity, low voltage {IT}| electricity voltage transformation from medium to low voltage | APOS, U.

[14]Electric energy necessary to use the heating stirrer during the workup phase for 1 hour. It is calculated as power*time, considering 630W as the heating stirrer power. The Ecoinvent process used was: Electricity, low voltage {IT}| electricity voltage transformation from medium to low voltage | APOS, U.

[15]Electric energy necessary to use the rotavapor after the workup phase for 1 hour. It is calculated as power*time, considering 500W as the rotavapor power. The Ecoinvent process used was: Electricity, low voltage {IT}| electricity voltage transformation from medium to low voltage | APOS, U.

[16]Electric energy necessary to the use of the aspiration system for the whole synthesis time, i.e. 11 h. The power was calculated by considering the air flow rate of 250 m3/h, a total load loss of 110.8076 kg/m2, and an efficiency of 90%: Electricity, low voltage {IT}| electricity voltage transformation from medium to low voltage | APOS, U.

[17]Amount of Acetone released into the atmosphere, as calculated by the formula reported in equation 4 of the main manuscript.

[18]Amount of HBr released into the atmosphere, as calculated by the formula reported in equation 4 of the main manuscript.

[19]Amount of Water released into the atmosphere, as calculated by the formula reported in equation 4 of the main manuscript.

[20]Amount of Diethyl ether released into the atmosphere, as calculated by the formula reported in equation 4 of the main manuscript.

[21]End of life of liquid waste, including Acetone, Diethyl ether and water. The Ecoinvent process used was: Spent solvent mixture {Europe without Switzerland}| treatment of spent solvent mixture, hazardous waste incineration, with energy recovery | APOS, U.

[22]End of life of the solid waste recovered after the filtration following the first part of the synthesis. The Ecoinvent process used was: Hazardous waste, for incineration {RoW}| treatment of hazardous waste, hazardous waste incineration, with energy recovery | APOS, U.

Table S34. Contributions to the Life Cycle Inventory (LCI) for the synthesis of 0.33116 g of 4-butoxy-3-methoxybenzaldehyde. This is Reaction 11 (Run 9), carried in Acetonitrile as solvent with a reaction time of 8 hours, as reported in Table 1 in the main manuscript.

Description			Amount	Process Data Source
Input	Materials	ACN	15.66 g	Ecoinvent v 3.8[1]
		K_2CO_3	0.974 g	Ecoinvent v 3.8[2]
		KCl	1.159 g	Ecoinvent v 3.8[3]
		Bromobutane	0.492 g	Modelled from Ecoinvent v 3.8 database sub process as detailed in Table S23
		Vanillin	0.504 g	Modelled from Ecoinvent v 3.8 database sub process as detailed in Table S20
		H_2O	5 g	Ecoinvent v 3.8[4]
		Diethyl ether	21.405 g	Ecoinvent v 3.8[5]
		Mg_2SO_4	1 g	Ecoinvent v 3.8[6]
		Silicon oil	$1.72*10^{-2}$ p	Ecoinvent v 3.8[7]
	Equipment/ plants	Magnetic stirrer/heater	$2*10^{-4}$ p	Modelled from Ecoinvent v 3.8 database sub process as detailed in Table S18
		Round bottom flask	$3.47*10^{-4}$ p	Modelled from Ecoinvent v 3.8 database sub process as detailed in Table S15
		Separatory funnel	$2.33*10^{-5}$ p	Modelled from Ecoinvent v 3.8 database sub process as detailed in Table S9
		Reflux condenser	$3.12*10^{-4}$ p	Modelled from Ecoinvent v 3.8 database sub process as detailed in Table S13
		Silicon oil container	$3.47*10^{-4}$ p	Modelled from Ecoinvent v 3.8 database sub process as detailed in Table S6
		Funnel	$2.90*10^{-6}$ p	Modelled from Ecoinvent v 3.8 database sub process as detailed in Table S8

		Filter	$3.18*10^{-6}$ p	Modelled from Ecoinvent v 3.8 database sub process as detailed in Table S7
		Fitting reflux condenser	$2.78*10^{-4}$ p	Modelled from Ecoinvent v 3.8 database sub process as detailed in Table S12
		Rubber tube	$5.56*10^{-4}$ p	Modelled from Ecoinvent v 3.8 database sub process as detailed in Table S14
		Activated carbon air filter	$5.5*10^{-4}$ p	Modelled from Ecoinvent v 3.8 database sub process as detailed in Table S4
		Aspiration system	$1.38*10^{-4}$ p	Modelled from Ecoinvent v 3.8 database sub process as detailed in Table S3
	Transport	Transport for large equipment	$2.29*10^{-2}$ tkm	Ecoinvent v 3.8[8]
		Transport for raw materials	8.73 kgkm	Ecoinvent v 3.8[9]
		Transport for small equipment	$7.44*10^{-2}$ kgkm	Ecoinvent v 3.8[10]
	Energy	Electric energy	28.25 kJ	Ecoinvent v 3.8[11]
			1056 Wh	Ecoinvent v 3.8[12]
			11.37 kJ	Ecoinvent v 3.8[13]
			630 Wh	Ecoinvent v 3.8[14]
			500 Wh	Ecoinvent v 3.8[15]
			0.92 kWh	Ecoinvent v 3.8[16]
Output	Air emission	ACN	$4.28*10^{-5}$ g	SimaPro airborne emission substance list[17]
		HBr	$3.42*10^{-3}$ g	SimaPro airborne emission substance list[18]
		H_2O	$1.15*10^{-6}$ g	SimaPro airborne emission substance list[19]
		Diethyl ether	$5.291*10^{-4}$ g	SimaPro airborne emission substance list[20]
	End of life	Spent solvent mixture	42.065 g	Ecoinvent v 3.8[21]

	Incineration	3.132 g	Ecoinvent v 3.8[22]

[1]The Ecoinvnet process used was: Acetonitrile {RER}| Sohio process | APOS, U.

[2]The Ecoinvent process used was: Potassium carbonate {RER}| oxidation of manganese dioxide | APOS, U.

[3]The Ecoinvent process used was: Potassium chloride {RER}| potassium chloride production | APOS, U. During the experimental work potassium iodide was used. Since it is not present in the database, potassium chloride was used as proxy.

[4]The Ecoinvent process use was: Water, deionised {Europe without Switzerland}| water production, deionised | APOS, U.

[5]The Ecoinvent process used was: Diethyl ether, without water, in 99.95% solution state {RER}| ethylene hydration | APOS, U.

[6]The Ecoinvent process used was: Magnesium sulfate {RER}| production | APOS, U.

[7]The Ecoinvent process used was: Silicone product {RER}| production | APOS, U.

[8]Transport of large equipment (aspiration system and activated air carbon filter). The Ecoinvet process used was: Transport, freight, lorry 16-32 metric ton, EURO6 {RER}| transport, freight, lorry 16-32 metric ton, EURO6 | APOS, U. An average distance of 100km was considered.

[9]Transport for reagents. The Ecoinvent process used was: Transport, freight, lorry 3.5-7.5 metric ton, EURO6 {RER}| transport, freight, lorry 3.5-7.5 metric ton, EURO6 | APOS, U. An average distance of 100km was considered.

[10]Transport for small laboratory equipment. The Ecoinvent process used was: Transport, freight, lorry 3.5-7.5 metric ton, EURO6 {RER}| transport, freight, lorry 3.5-7.5 metric ton, EURO6 | APOS, U. An average distance of 100 km was considered.

[11]Electric energy necessary for the heating up to 100°C of the reaction mixture containing ACN, Vanillin and Bromobutane. It is calculated using the expression *m*Cp*DT*, considering the specific heat in J/kg°C for each of the substances. The Ecoinvent process used was: Electricity, low voltage {IT}| electricity voltage transformation from medium to low voltage | APOS, U.

[12]Electric energy necessary to maintain the heating at 100°C of the reaction mixture for 8 hours. It is calculated using the Fourier Equation. The Ecoinvent process used was: Electricity, low voltage {IT}| electricity voltage transformation from medium to low voltage | APOS, U.

[13]Electric energy necessary to eliminate the remaining solvent (ACN) using the rotavapor. It is calculated as HvapACN*molACN, considering HvapACN as its latent heat of vaporization (29.8 kJ/mol) and molACN as the moles of the solvent. The Ecoinvent process used was: Electricity, low voltage {IT}| electricity voltage transformation from medium to low voltage | APOS, U.

[14]Electric energy necessary to use the heating stirrer during the workup phase for 1 hour. It is calculated as power*time, considering 630W as the heating stirrer power. The Ecoinvent process used was: Electricity, low voltage {IT}| electricity voltage transformation from medium to low voltage | APOS, U.

[15]Electric energy necessary to use the rotavapor after the workup phase for 1 hour. It is calculated as power*time, considering 500W as the rotavapor power. The Ecoinvent process used was: Electricity, low voltage {IT}| electricity voltage transformation from medium to low voltage | APOS, U.

[16]Electric energy necessary to the use of the aspiration system for the whole synthesis time, i.e. 11 h. The power was calculated by considering the air flow rate of 250 m3/h, a total load loss of 110.8076 kg/m2, and an efficiency of 90%: Electricity, low voltage {IT}| electricity voltage transformation from medium to low voltage | APOS, U.

[17]Amount of ACN released into the atmosphere, as calculated by the formula reported in equation 4 of the main manuscript.

[18]Amount of HBr released into the atmosphere, as calculated by the formula reported in equation 4 of the main manuscript.

[19]Amount of Water released into the atmosphere, as calculated by the formula reported in equation 4 of the main manuscript.

[20]Amount of Diethyl ether released into the atmosphere, as calculated by the formula reported in equation 4 of the main manuscript.

[21]End of life of liquid waste, including ACN, Diethyl ether and water. The Ecoinvent process used was: Spent solvent mixture {Europe without Switzerland}| treatment of spent solvent mixture, hazardous waste incineration, with energy recovery | APOS, U.

[22]End of life of the solid waste recovered after the filtration following the first part of the synthesis. The Ecoinvent process used was: Hazardous waste, for incineration {RoW}| treatment of hazardous waste, hazardous waste incineration, with energy recovery | APOS, U.

Table S35. Contributions to the Life Cycle Inventory (LCI) for the synthesis of 0.0151 g of 4-butoxy-3-methoxybenzaldehyde. This is Reaction 12 (Run 5), carried in Acetone as solvent with a reaction time of 24 hours, as reported in Table 1 in the main manuscript.

Description			Amount	Process Data Source
Input	Materials	Acetone	15.68 g	Ecoinvent v 3.8[1]
		K_2CO_3	0.9073 g	Ecoinvent v 3.8[2]
		KCl	1.159 g	Ecoinvent v 3.8[3]
		Bromobutane	0.492 g	Modelled from Ecoinvent v 3.8 database sub process as detailed in Table S23
		Vanillin	0.50447 g	Modelled from Ecoinvent v 3.8 database sub process as detailed in Table S20
		H_2O	5 g	Ecoinvent v 3.8[4]
		Diethyl ether	21.405 g	Ecoinvent v 3.8[5]
		Mg_2SO_4	1 g	Ecoinvent v 3.8[6]
		Silicon oil	$1.72*10^{-2}$ p	Ecoinvent v 3.8[7]
	Equipment/ plants	Magnetic stirrer/heater	$5*10^{-4}$ p	Modelled from Ecoinvent v 3.8 database sub process as detailed in Table S18
		Round bottom flask	$9.03*10^{-4}$ p	Modelled from Ecoinvent v 3.8 database sub process as detailed in Table S15
		Separatory funnel	$2.33*10^{-5}$ p	Modelled from Ecoinvent v 3.8 database sub process as detailed in Table S9
		Reflux condenser	$8.69*10^{-4}$ p	Modelled from Ecoinvent v 3.8 database sub process as detailed in Table S13
		Silicon oil container	$9.03*10^{-4}$ p	Modelled from Ecoinvent v 3.8 database sub process as detailed in Table S6
		Funnel	$2.90*10^{-6}$ p	Modelled from Ecoinvent v 3.8 database sub process as detailed in Table S8

		Filter	3.18*10^{-6} p	Modelled from Ecoinvent v 3.8 database sub process as detailed in Table S7
		Fitting reflux condenser	8.69*10^{-4} p	Modelled from Ecoinvent v 3.8 database sub process as detailed in Table S12
		Rubber tube	1.67*10^{-3} p	Modelled from Ecoinvent v 3.8 database sub process as detailed in Table S14
		Activated carbon air filter	1.35*10^{-3} p	Modelled from Ecoinvent v 3.8 database sub process as detailed in Table S4
		Aspiration system	3.38*10^{-4} p	Modelled from Ecoinvent v 3.8 database sub process as detailed in Table S3
	Transport	Transport for large equipment	5.63*10^{-2} tkm	Ecoinvent v 3.8[8]
		Transport for raw materials	8.72 kgkm	Ecoinvent v 3.8[9]
		Transport for small equipment	1.91*10^{-1} kgkm	Ecoinvent v 3.8[10]
	Energy	Electric energy	11.40 kJ	Ecoinvent v 3.8[11]
			1440 Wh	Ecoinvent v 3.8[12]
			8.64 kJ	Ecoinvent v 3.8[13]
			630 Wh	Ecoinvent v 3.8[14]
			500 Wh	Ecoinvent v 3.8[15]
			2.26 kWh	Ecoinvent v 3.8[16]
Output	Air emission	Acetone	1.44*10^{-4} g	SimaPro airborne emission substance list[17]
		HBr	1.57*10^{-3} g	SimaPro airborne emission substance list[18]
		H_2O	1.15*10^{-6} g	SimaPro airborne emission substance list[19]
		Diethyl ether	5.29*10^{-4} g	SimaPro airborne emission substance list[20]
	End of life	Spent solvent mixture	42.085 g	Ecoinvent v 3.8[21]

	Incineration	3.067 g	Ecoinvent v 3.8[22]

[1]The Ecoinvnet process used was: Acetone, liquid {RER}| production | APOS, U

[2]The Ecoinvent process used was: Potassium carbonate {RER}| oxidation of manganese dioxide | APOS, U.

[3]The Ecoinvent process used was: Potassium chloride {RER}| potassium chloride production | APOS, U. During the experimental work potassium iodide was used. Since it is not present in the database, potassium chloride was used as proxy.

[4]The Ecoinvent process use was: Water, deionised {Europe without Switzerland}| water production, deionised | APOS, U.

[5]The Ecoinvent process used was: Diethyl ether, without water, in 99.95% solution state {RER}| ethylene hydration | APOS, U.

[6]The Ecoinvent process used was: Magnesium sulfate {RER}| production | APOS, U.

[7]The Ecoinvent process used was: Silicone product {RER}| production | APOS, U.

[8]Transport of large equipment (aspiration system and activated air carbon filter). The Ecoinvet process used was: Transport, freight, lorry 16-32 metric ton, EURO6 {RER}| transport, freight, lorry 16-32 metric ton, EURO6 | APOS, U. An average distance of 100km was considered.

[9]Transport for reagents. The Ecoinvent process used was: Transport, freight, lorry 3.5-7.5 metric ton, EURO6 {RER}| transport, freight, lorry 3.5-7.5 metric ton, EURO6 | APOS, U. An average distance of 100km was considered.

[10]Transport for small laboratory equipment. The Ecoinvent process used was: Transport, freight, lorry 3.5-7.5 metric ton, EURO6 {RER}| transport, freight, lorry 3.5-7.5 metric ton, EURO6 | APOS, U. An average distance of 100 km was considered.

[11]Electric energy necessary for the heating up to 100°C of the reaction mixture containing Acetone, Vanillin and Bromobutane. It is calculated using the expression *m*Cp*DT*, considering the specific heat in J/kg°C for each of the substances. The Ecoinvent process used was: Electricity, low voltage {IT}| electricity voltage transformation from medium to low voltage | APOS, U.

[12]Electric energy necessary to maintain the heating at 100°C of the reaction mixture for 24 hours. It is calculated using the Fourier Equation. The Ecoinvent process used was: Electricity, low voltage {IT}| electricity voltage transformation from medium to low voltage | APOS, U.

[13]Electric energy necessary to eliminate the remaining solvent (ACN) using the rotavapor. It is calculated as HvapAcetone*molAcetone, considering HvapAcentone as its latent heat of vaporization (32 kJ/mol) and molAcetone as the moles of the solvent. The Ecoinvent process used was: Electricity, low voltage {IT}| electricity voltage transformation from medium to low voltage | APOS, U.

[14]Electric energy necessary to use the heating stirrer during the workup phase for 1 hour. It is calculated as power*time, considering 630W as the heating stirrer power. The Ecoinvent process used was: Electricity, low voltage {IT}| electricity voltage transformation from medium to low voltage | APOS, U.

[15]Electric energy necessary to use the rotavapor after the workup phase for 1 hour. It is calculated as power*time, considering 500W as the rotavapor power. The Ecoinvent process used was: Electricity, low voltage {IT}| electricity voltage transformation from medium to low voltage | APOS, U.

[16]Electric energy necessary to the use of the aspiration system for the whole synthesis time, i.e. 27 h. The power was calculated by considering the air flow rate of 250 m3/h, a total load loss of 110.8076 kg/m2, and an efficiency of 90%: Electricity, low voltage {IT}| electricity voltage transformation from medium to low voltage | APOS, U.

[17]Amount of Acetone released into the atmosphere, as calculated by the formula reported in equation 4 of the main manuscript.

[18]Amount of HBr released into the atmosphere, as calculated by the formula reported in equation 4 of the main manuscript.

[19]Amount of Water released into the atmosphere, as calculated by the formula reported in equation 4 of the main manuscript.

[20]Amount of Diethyl ether released into the atmosphere, as calculated by the formula reported in equation 4 of the main manuscript.

[21]End of life of liquid waste, including Acetone, Diethyl ether and water. The Ecoinvent process used was: Spent solvent mixture {Europe without Switzerland}| treatment of spent solvent mixture, hazardous waste incineration, with energy recovery | APOS, U.

[22]End of life of the solid waste recovered after the filtration following the first part of the synthesis. The Ecoinvent process used was: Hazardous waste, for incineration {RoW}| treatment of hazardous waste, hazardous waste incineration, with energy recovery | APOS, U.

Table S36. Contributions to the Life Cycle Inventory (LCI) for the synthesis of 0.37416 g of 4-butoxy-3-methoxybenzaldehyde. This is Reaction 13 (Run 8), carried in Acetonitrile as solvent with a reaction time of 24 hours, as reported in Table 1 in the main manuscript.

Description			Amount	Process Data Source
Input	Materials	ACN	15.66 g	Ecoinvent v 3.8[1]
		K_2CO_3	0.914 g	Ecoinvent v 3.8[2]
		KCl	0 g	Ecoinvent v 3.8[3]
		Bromobutane	0.922 g	Modelled from Ecoinvent v 3.8 database sub process as detailed in Table S23
		Vanillin	0.5181 g	Modelled from Ecoinvent v 3.8 database sub process as detailed in Table S20
		H_2O	5 g	Ecoinvent v 3.8[4]
		Diethyl ether	21.405 g	Ecoinvent v 3.8[5]
		Mg_2SO_4	1 g	Ecoinvent v 3.8[6]
		Silicon oil	$1.72*10^{-2}$ p	Ecoinvent v 3.8[7]
	Equipment/ plants	Magnetic stirrer/heater	$5*10^{-4}$ p	Modelled from Ecoinvent v 3.8 database sub process as detailed in Table S18
		Round bottom flask	$9.03*10^{-4}$ p	Modelled from Ecoinvent v 3.8 database sub process as detailed in Table S15
		Separatory funnel	$2.33*10^{-5}$ p	Modelled from Ecoinvent v 3.8 database sub process as detailed in Table S9
		Reflux condenser	$8.69*10^{-4}$ p	Modelled from Ecoinvent v 3.8 database sub process as detailed in Table S13
		Silicon oil container	$9.03*10^{-4}$ p	Modelled from Ecoinvent v 3.8 database sub process as detailed in Table S6
		Funnel	$2.90*10^{-6}$ p	Modelled from Ecoinvent v 3.8 database sub process as detailed in Table S8

		Filter	$3.18*10^{-6}$ p	Modelled from Ecoinvent v 3.8 database sub process as detailed in Table S7
		Fitting reflux condenser	$8.69*10^{-4}$ p	Modelled from Ecoinvent v 3.8 database sub process as detailed in Table S12
		Rubber tube	$1.67*10^{-2}$ p	Modelled from Ecoinvent v 3.8 database sub process as detailed in Table S14
		Activated carbon air filter	$1.35*10^{-3}$ p	Modelled from Ecoinvent v 3.8 database sub process as detailed in Table S4
		Aspiration system	$3.38*10^{-4}$ p	Modelled from Ecoinvent v 3.8 database sub process as detailed in Table S3
	Transport	Transport for large equipment	$5.63*10^{-2}$ tkm	Ecoinvent v 3.8[8]
		Transport for raw materials	8.58 kgkm	Ecoinvent v 3.8[9]
		Transport for small equipment	$1.91*10^{-1}$ kgkm	Ecoinvent v 3.8[10]
	Energy	Electric energy	28.25 kJ	Ecoinvent v 3.8[11]
			3168 Wh	Ecoinvent v 3.8[12]
			11.37 kJ	Ecoinvent v 3.8[13]
			630 Wh	Ecoinvent v 3.8[14]
			500 Wh	Ecoinvent v 3.8[15]
			2.26 kWh	Ecoinvent v 3.8[16]
Output	Air emission	ACN	$4.28*10^{-5}$ g	SimaPro airborne emission substance list[17]
		HBr	$3.83*10^{-3}$ g	SimaPro airborne emission substance list[18]
		H_2O	$1.15*10^{-6}$ g	SimaPro airborne emission substance list[19]
		Diethyl ether	$5.291*10^{-4}$ g	SimaPro airborne emission substance list[20]
	End of life	Spent solvent mixture	42.065 g	Ecoinvent v 3.8[21]

	Incineration	1.914g	Ecoinvent v 3.8[22]

[1]The Ecoinvnet process used was: Acetonitrile {RER}| Sohio process | APOS, U.

[2]The Ecoinvent process used was: Potassium carbonate {RER}| oxidation of manganese dioxide | APOS, U.

[3]The Ecoinvent process used was: Potassium chloride {RER}| potassium chloride production | APOS, U. During the experimental work potassium iodide was used. Since it is not present in the database, potassium chloride was used as proxy. In this specific run, this reagent wad not used, for this reason its quantity is zero in this table.

[4]The Ecoinvent process use was: Water, deionised {Europe without Switzerland}| water production, deionised | APOS, U.

[5]The Ecoinvent process used was: Diethyl ether, without water, in 99.95% solution state {RER}| ethylene hydration | APOS, U.

[6]The Ecoinvent process used was: Magnesium sulfate {RER}| production | APOS, U.

[7]The Ecoinvent process used was: Silicone product {RER}| production | APOS, U.

[8]Transport of large equipment (aspiration system and activated air carbon filter). The Ecoinvet process used was: Transport, freight, lorry 16-32 metric ton, EURO6 {RER}| transport, freight, lorry 16-32 metric ton, EURO6 | APOS, U. An average distance of 100km was considered.

[9]Transport for reagents. The Ecoinvent process used was: Transport, freight, lorry 3.5-7.5 metric ton, EURO6 {RER}| transport, freight, lorry 3.5-7.5 metric ton, EURO6 | APOS, U. An average distance of 100km was considered.

[10]Transport for small laboratory equipment. The Ecoinvent process used was: Transport, freight, lorry 3.5-7.5 metric ton, EURO6 {RER}| transport, freight, lorry 3.5-7.5 metric ton, EURO6 | APOS, U. An average distance of 100 km was considered.

[11]Electric energy necessary for the heating up to 100°C of the reaction mixture containing ACN, Vanillin and Bromobutane. It is calculated using the expression *m*Cp*DT*, considering the specific heat in J/kg°C for each of the substances. The Ecoinvent process used was: Electricity, low voltage {IT}| electricity voltage transformation from medium to low voltage | APOS, U.

[12]Electric energy necessary to maintain the heating at 100°C of the reaction mixture for 24 hours. It is calculated using the Fourier Equation. The Ecoinvent process used was: Electricity, low voltage {IT}| electricity voltage transformation from medium to low voltage | APOS, U.

[13]Electric energy necessary to eliminate the remaining solvent (ACN) using the rotavapor. It is calculated as HvapACN*molACN, considering HvapACN as its latent heat of vaporization (29.8 kJ/mol) and molACN as the moles of the solvent. The Ecoinvent process used was: Electricity, low voltage {IT}| electricity voltage transformation from medium to low voltage | APOS, U.

[14]Electric energy necessary to use the heating stirrer during the workup phase for 1 hour. It is calculated as power*time, considering 630W as the heating stirrer power. The Ecoinvent process used was: Electricity, low voltage {IT}| electricity voltage transformation from medium to low voltage | APOS, U.

[15]Electric energy necessary to use the rotavapor after the workup phase for 1 hour. It is calculated as power*time, considering 500W as the rotavapor power. The Ecoinvent process used was: Electricity, low voltage {IT}| electricity voltage transformation from medium to low voltage | APOS, U.

[16]Electric energy necessary to the use of the aspiration system for the whole synthesis time, i.e. 27 h. The power was calculated by considering the air flow rate of 250 m3/h, a total load loss of 110.8076 kg/m2, and an efficiency of 90%: Electricity, low voltage {IT}| electricity voltage transformation from medium to low voltage | APOS, U.

[17]Amount of ACN released into the atmosphere, as calculated by the formula reported in equation 4 of the main manuscript.

[18]Amount of HBr released into the atmosphere, as calculated by the formula reported in equation 4 of the main manuscript.

[19]Amount of Water released into the atmosphere, as calculated by the formula reported in equation 4 of the main manuscript.

[20]Amount of Diethyl ether released into the atmosphere, as calculated by the formula reported in equation 4 of the main manuscript.

[21]End of life of liquid waste, including ACN, Diethyl ether and water. The Ecoinvent process used was: Spent solvent mixture {Europe without Switzerland}| treatment of spent solvent mixture, hazardous waste incineration, with energy recovery | APOS, U.

[22]End of life of the solid waste recovered after the filtration following the first part of the synthesis. The Ecoinvent process used was: Hazardous waste, for incineration {RoW}| treatment of hazardous waste, hazardous waste incineration, with energy recovery | APOS, U.

Table S37. Contributions to the Life Cycle Inventory (LCI) for the synthesis of 0.11076 g of 4-butoxy-3-methoxybenzaldehyde. This is Reaction 14 (Run 19), carried in Acetonitrile as solvent with a reaction time of 16 hours, as reported in Table 1 in the main manuscript.

Description			Amount	Process Data Source
Input	Materials	ACN	15.66 g	Ecoinvent v 3.8[1]
		K_2CO_3	0.676 g	Ecoinvent v 3.8[2]
		KCl	0.649 g	Ecoinvent v 3.8[3]
		Bromobutane	0.679 g	Modelled from Ecoinvent v 3.8 database sub process as detailed in Table S23
		Vanillin	0.520 g	Modelled from Ecoinvent v 3.8 database sub process as detailed in Table S20
		H_2O	5 g	Ecoinvent v 3.8[4]
		Diethyl ether	21.405 g	Ecoinvent v 3.8[5]
		Mg_2SO_4	1 g	Ecoinvent v 3.8[6]
		Silicon oil	$1.72*10^{-2}$ p	Ecoinvent v 3.8[7]
	Equipment/ plants	Magnetic stirrer/heater	$3.4*10^{-4}$ p	Modelled from Ecoinvent v 3.8 database sub process as detailed in Table S18
		Round bottom flask	$6.25*10^{-4}$ p	Modelled from Ecoinvent v 3.8 database sub process as detailed in Table S15
		Separatory funnel	$2.33*10^{-5}$ p	Modelled from Ecoinvent v 3.8 database sub process as detailed in Table S9
		Reflux condenser	$5.90*10^{-4}$ p	Modelled from Ecoinvent v 3.8 database sub process as detailed in Table S13
		Silicon oil container	$9.03*10^{-4}$ p	Modelled from Ecoinvent v 3.8 database sub process as detailed in Table S6
		Funnel	$2.90*10^{-6}$ p	Modelled from Ecoinvent v 3.8 database sub process as detailed in Table S8
		Filter	$3.18*10^{-6}$ p	Modelled from Ecoinvent v 3.8 database

				sub process as detailed in Table S7
		Fitting reflux condenser	$5.56*10^{-4}$ p	Modelled from Ecoinvent v 3.8 database sub process as detailed in Table S12
		Rubber tube	$1.11*10^{-2}$ p	Modelled from Ecoinvent v 3.8 database sub process as detailed in Table S14
		Activated carbon air filter	$9.5*10^{-4}$ p	Modelled from Ecoinvent v 3.8 database sub process as detailed in Table S4
		Aspiration system	$2.38*10^{-4}$ p	Modelled from Ecoinvent v 3.8 database sub process as detailed in Table S3
	Transport	Transport for large equipment	$3.97*10^{-2}$ tkm	Ecoinvent v 3.8[8]
		Transport for raw materials	8.62 kgkm	Ecoinvent v 3.8[9]
		Transport for small equipment	$1.30*10^{-1}$ kgkm	Ecoinvent v 3.8[10]
	Energy	Electric energy	28.25 kJ	Ecoinvent v 3.8[11]
			2112 Wh	Ecoinvent v 3.8[12]
			11.37 kJ	Ecoinvent v 3.8[13]
			630 Wh	Ecoinvent v 3.8[14]
			500 Wh	Ecoinvent v 3.8[15]
			2.26 kWh	Ecoinvent v 3.8[16]
Output	Air emission	ACN	$4.28*10^{-5}$ g	SimaPro airborne emission substance list[17]
		HBr	$1.21*10^{-3}$ g	SimaPro airborne emission substance list[18]
		H_2O	$1.15*10^{-6}$ g	SimaPro airborne emission substance list[19]
		Diethyl ether	$5.291*10^{-4}$ g	SimaPro airborne emission substance list[20]
	End of life	Spent solvent mixture	42.065 g	Ecoinvent v 3.8[21]
		Incineration	2.326 g	Ecoinvent v 3.8[22]

[1]The Ecoinvnet process used was: Acetonitrile {RER}| Sohio process | APOS, U.

[2]The Ecoinvent process used was: Potassium carbonate {RER}| oxidation of manganese dioxide | APOS, U.
[3]The Ecoinvent process used was: Potassium chloride {RER}| potassium chloride production | APOS, U. During the experimental work potassium iodide was used. Since it is not present in the database, potassium chloride was used as proxy.
[4]The Ecoinvent process use was: Water, deionised {Europe without Switzerland}| water production, deionised | APOS, U.
[5]The Ecoinvent process used was: Diethyl ether, without water, in 99.95% solution state {RER}| ethylene hydration | APOS, U.
[6]The Ecoinvent process used was: Magnesium sulfate {RER}| production | APOS, U.
[7]The Ecoinvent process used was: Silicone product {RER}| production | APOS, U.
[8]Transport of large equipment (aspiration system and activated air carbon filter). The Ecoinvet process used was: Transport, freight, lorry 16-32 metric ton, EURO6 {RER}| transport, freight, lorry 16-32 metric ton, EURO6 | APOS, U. An average distance of 100km was considered.
[9]Transport for reagents. The Ecoinvent process used was: Transport, freight, lorry 3.5-7.5 metric ton, EURO6 {RER}| transport, freight, lorry 3.5-7.5 metric ton, EURO6 | APOS, U. An average distance of 100km was considered.
[10]Transport for small laboratory equipment. The Ecoinvent process used was: Transport, freight, lorry 3.5-7.5 metric ton, EURO6 {RER}| transport, freight, lorry 3.5-7.5 metric ton, EURO6 | APOS, U. An average distance of 100 km was considered.
[11]Electric energy necessary for the heating up to 100°C of the reaction mixture containing ACN, Vanillin and Bromobutane. It is calculated using the expression *m*Cp*DT*, considering the specific heat in J/kg°C for each of the substances. The Ecoinvent process used was: Electricity, low voltage {IT}| electricity voltage transformation from medium to low voltage | APOS, U.
[12]Electric energy necessary to maintain the heating at 100°C of the reaction mixture for 16 hours. It is calculated using the Fourier Equation. The Ecoinvent process used was: Electricity, low voltage {IT}| electricity voltage transformation from medium to low voltage | APOS, U.
[13]Electric energy necessary to eliminate the remaining solvent (ACN) using the rotavapor. It is calculated as HvapACN*molACN, considering HvapACN as its latent heat of vaporization (29.8 kJ/mol) and molACN as the moles of the solvent. The Ecoinvent process used was: Electricity, low voltage {IT}| electricity voltage transformation from medium to low voltage | APOS, U.
[14]Electric energy necessary to use the heating stirrer during the workup phase for 1 hour. It is calculated as power*time, considering 630W as the heating stirrer power. The Ecoinvent process used was: Electricity, low voltage {IT}| electricity voltage transformation from medium to low voltage | APOS, U.
[15]Electric energy necessary to use the rotavapor after the workup phase for 1 hour. It is calculated as power*time, considering 500W as the rotavapor power. The Ecoinvent process used was: Electricity, low voltage {IT}| electricity voltage transformation from medium to low voltage | APOS, U.
[16]Electric energy necessary to the use of the aspiration system for the whole synthesis time, i.e. 19 h. The power was calculated by considering the air flow rate of 250 m3/h, a total load loss of 110.8076 kg/m2, and an efficiency of 90%: Electricity, low voltage {IT}| electricity voltage transformation from medium to low voltage | APOS, U.
[17]Amount of ACN released into the atmosphere, as calculated by the formula reported in equation 4 of the main manuscript.
[18]Amount of HBr released into the atmosphere, as calculated by the formula reported in equation 4 of the main manuscript.
[19]Amount of Water released into the atmosphere, as calculated by the formula reported in equation 4 of the main manuscript.
[20]Amount of Diethyl ether released into the atmosphere, as calculated by the formula reported in equation 4 of the main manuscript.
[21]End of life of liquid waste, including ACN, Diethyl ether and water. The Ecoinvent process used was: Spent solvent mixture {Europe without Switzerland}| treatment of spent solvent mixture, hazardous waste incineration, with energy recovery | APOS, U.
[22]End of life of the solid waste recovered after the filtration following the first part of the synthesis. The Ecoinvent process used was: Hazardous waste, for incineration {RoW}| treatment of hazardous waste, hazardous waste incineration, with energy recovery | APOS, U.

Table S38. Contributions to the Life Cycle Inventory (LCI) for the synthesis of 0.32881 g of 4-butoxy-3-methoxybenzaldehyde. This is Reaction 15 (Run 10), carried in DMF as solvent with a reaction time of 24 hours, as reported in Table 1 in the main manuscript.

Description			Amount	Process Data Source
Input	Materials	DMF	4.72 g	Ecoinvent v 3.8[1]
		K_2CO_3	0.452 g	Ecoinvent v 3.8[2]
		KCl	0 g	Ecoinvent v 3.8[3]
		Bromobutane	0.456 g	Modelled from Ecoinvent v 3.8 database sub process as detailed in Table S23
		Vanillin	0.504 g	Modelled from Ecoinvent v 3.8 database sub process as detailed in Table S20
		H_2O	5 g	Ecoinvent v 3.8[4]
		Diethyl ether	21.405 g	Ecoinvent v 3.8[5]
		Mg_2SO_4	1 g	Ecoinvent v 3.8[6]
		Silicon oil	$1.72*10^{-2}$ p	Ecoinvent v 3.8[7]
	Equipment/ plants	Magnetic stirrer/heater	$5*10^{-4}$ p	Modelled from Ecoinvent v 3.8 database sub process as detailed in Table S18
		Round bottom flask	$9.03*10^{-4}$ p	Modelled from Ecoinvent v 3.8 database sub process as detailed in Table S15
		Separatory funnel	$2.33*10^{-5}$ p	Modelled from Ecoinvent v 3.8 database sub process as detailed in Table S9
		Reflux condenser	$8.68*10^{-4}$ p	Modelled from Ecoinvent v 3.8 database sub process as detailed in Table S13
		Silicon oil container	$9.03*10^{-4}$ p	Modelled from Ecoinvent v 3.8 database sub process as detailed in Table S6
		Funnel	$2.90*10^{-6}$ p	Modelled from Ecoinvent v 3.8 database sub process as detailed in Table S8
		Filter	$3.18*10^{-6}$ p	Modelled from Ecoinvent v 3.8 database

				sub process as detailed in Table S7
		Fitting reflux condenser	$8.68*10^{-4}$ p	Modelled from Ecoinvent v 3.8 database sub process as detailed in Table S12
		Rubber tube	$1.67*10^{-3}$ p	Modelled from Ecoinvent v 3.8 database sub process as detailed in Table S14
		Activated carbon air filter	$1.35*10^{-3}$ p	Modelled from Ecoinvent v 3.8 database sub process as detailed in Table S4
		Aspiration system	$3.38*10^{-4}$ p	Modelled from Ecoinvent v 3.8 database sub process as detailed in Table S3
	Transport	Transport for large equipment	$5.63*10^{-2}$ tkm	Ecoinvent v 3.8[8]
		Transport for raw materials	6.34 kgkm	Ecoinvent v 3.8[9]
		Transport for small equipment	$1.91*10^{-1}$ kgkm	Ecoinvent v 3.8[10]
	Energy	Electric energy	42.90 kJ	Ecoinvent v 3.8[11]
			4320 Wh	Ecoinvent v 3.8[12]
			3.015 kJ	Ecoinvent v 3.8[13]
			630 Wh	Ecoinvent v 3.8[14]
			500 Wh	Ecoinvent v 3.8[15]
			2.26 kWh	Ecoinvent v 3.8[16]
Output	Air emission	DMF	$7.28*10^{-7}$ g	SimaPro airborne emission substance list[17]
		HBr	$5.25*10^{-3}$ g	SimaPro airborne emission substance list[18]
		H_2O	$1.15*10^{-6}$ g	SimaPro airborne emission substance list[19]
		Diethyl ether	$5.291*10^{-4}$ g	SimaPro airborne emission substance list[20]
	End of life	Spent solvent mixture	31.125 g	Ecoinvent v 3.8[21]
		Incineration	1.452 g	Ecoinvent v 3.8[22]

[1]The Ecoinvnet process used was: N,N-dimethylformamide {RER}| production | APOS, U.

[2]The Ecoinvent process used was: Potassium carbonate {RER}| oxidation of manganese dioxide | APOS, U.
[3]The Ecoinvent process used was: Potassium chloride {RER}| potassium chloride production | APOS, U. During the experimental work potassium iodide was used. Since it is not present in the database, potassium chloride was used as proxy. In this specific run, this reagent wad not used, for this reason its quantity is zero in this table.
[4]The Ecoinvent process use was: Water, deionised {Europe without Switzerland}| water production, deionised | APOS, U.
[5]The Ecoinvent process used was: Diethyl ether, without water, in 99.95% solution state {RER}| ethylene hydration | APOS, U.
[6]The Ecoinvent process used was: Magnesium sulfate {RER}| production | APOS, U.
[7]The Ecoinvent process used was: Silicone product {RER}| production | APOS, U.
[8]Transport of large equipment (aspiration system and activated air carbon filter). The Ecoinvet process used was: Transport, freight, lorry 16-32 metric ton, EURO6 {RER}| transport, freight, lorry 16-32 metric ton, EURO6 | APOS, U. An average distance of 100km was considered.
[9]Transport for reagents. The Ecoinvent process used was: Transport, freight, lorry 3.5-7.5 metric ton, EURO6 {RER}| transport, freight, lorry 3.5-7.5 metric ton, EURO6 | APOS, U. An average distance of 100km was considered.
[10]Transport for small laboratory equipment. The Ecoinvent process used was: Transport, freight, lorry 3.5-7.5 metric ton, EURO6 {RER}| transport, freight, lorry 3.5-7.5 metric ton, EURO6 | APOS, U. An average distance of 100 km was considered.
[11]Electric energy necessary for the heating up to 100°C of the reaction mixture containing DMF, Vanillin and Bromobutane. It is calculated using the expression *m*Cp*DT*, considering the specific heat in J/kg°C for each of the substances. The Ecoinvent process used was: Electricity, low voltage {IT}| electricity voltage transformation from medium to low voltage | APOS, U.
[12]Electric energy necessary to maintain the heating at 100°C of the reaction mixture for 24 hours. It is calculated using the Fourier Equation. The Ecoinvent process used was: Electricity, low voltage {IT}| electricity voltage transformation from medium to low voltage | APOS, U.
[13]Electric energy necessary to eliminate the remaining solvent (DMF) using the rotavapor. It is calculated as HvapDMF*molDMF, considering HvapDMF as its latent heat of vaporization (46.7 kJ/mol) and molDMF as the moles of the solvent. The Ecoinvent process used was: Electricity, low voltage {IT}| electricity voltage transformation from medium to low voltage | APOS, U.
[14]Electric energy necessary to use the heating stirrer during the workup phase for 1 hour. It is calculated as power*time, considering 630W as the heating stirrer power. The Ecoinvent process used was: Electricity, low voltage {IT}| electricity voltage transformation from medium to low voltage | APOS, U.
[15]Electric energy necessary to use the rotavapor after the workup phase for 1 hour. It is calculated as power*time, considering 500W as the rotavapor power. The Ecoinvent process used was: Electricity, low voltage {IT}| electricity voltage transformation from medium to low voltage | APOS, U.
[16]Electric energy necessary to the use of the aspiration system for the whole synthesis time, i.e. 27 h. The power was calculated by considering the air flow rate of 250 m3/h, a total load loss of 110.8076 kg/m2, and an efficiency of 90%: Electricity, low voltage {IT}| electricity voltage transformation from medium to low voltage | APOS, U.
[17]Amount of DMF released into the atmosphere, as calculated by the formula reported in equation 4 of the main manuscript.
[18]Amount of HBr released into the atmosphere, as calculated by the formula reported in equation 4 of the main manuscript.
[19]Amount of Water released into the atmosphere, as calculated by the formula reported in equation 4 of the main manuscript.
[20]Amount of Diethyl ether released into the atmosphere, as calculated by the formula reported in equation 4 of the main manuscript.
[21]End of life of liquid waste, including DMF, Diethyl ether and water. The Ecoinvent process used was: Spent solvent mixture {Europe without Switzerland}| treatment of spent solvent mixture, hazardous waste incineration, with energy recovery | APOS, U.
[22]End of life of the solid waste recovered after the filtration following the first part of the synthesis. The Ecoinvent process used was: Hazardous waste, for incineration {RoW}| treatment of hazardous waste, hazardous waste incineration, with energy recovery | APOS, U.

Table S39. Contributions to the Life Cycle Inventory (LCI) for the synthesis of 0.11867 g of 4-butoxy-3-methoxybenzaldehyde. This is Reaction 16 (Run 18), carried in Acetonitrile as solvent with a reaction time of 16 hours, as reported in Table 1 in the main manuscript.

Description			Amount	Process Data Source
Input	Materials	ACN	15.66 g	Ecoinvent v 3.8[1]
		K_2CO_3	0.683 g	Ecoinvent v 3.8[2]
		KCl	0.543 g	Ecoinvent v 3.8[3]
		Bromobutane	0.687 g	Modelled from Ecoinvent v 3.8 database sub process as detailed in Table S23
		Vanillin	0.503 g	Modelled from Ecoinvent v 3.8 database sub process as detailed in Table S20
		H_2O	5 g	Ecoinvent v 3.8[4]
		Diethyl ether	21.405 g	Ecoinvent v 3.8[5]
		Mg_2SO_4	1 g	Ecoinvent v 3.8[6]
		Silicon oil	$1.72*10^{-2}$ p	Ecoinvent v 3.8[7]
	Equipment/ plants	Magnetic stirrer/heater	$3.4*10^{-4}$ p	Modelled from Ecoinvent v 3.8 database sub process as detailed in Table S18
		Round bottom flask	$6.25*10^{-4}$ p	Modelled from Ecoinvent v 3.8 database sub process as detailed in Table S15
		Separatory funnel	$2.33*10^{-5}$ p	Modelled from Ecoinvent v 3.8 database sub process as detailed in Table S9
		Reflux condenser	$5.90*10^{-4}$ p	Modelled from Ecoinvent v 3.8 database sub process as detailed in Table S13
		Silicon oil container	$6.25*10^{-4}$ p	Modelled from Ecoinvent v 3.8 database sub process as detailed in Table S6
		Funnel	$2.90*10^{-6}$ p	Modelled from Ecoinvent v 3.8 database sub process as detailed in Table S8
		Filter	$3.18*10^{-6}$ p	Modelled from Ecoinvent v 3.8 database

				sub process as detailed in Table S7
		Fitting reflux condenser	$5.56*10^{-4}$ p	Modelled from Ecoinvent v 3.8 database sub process as detailed in Table S12
		Rubber tube	$1.11*10^{-2}$ p	Modelled from Ecoinvent v 3.8 database sub process as detailed in Table S14
		Activated carbon air filter	$9.5*10^{-4}$ p	Modelled from Ecoinvent v 3.8 database sub process as detailed in Table S4
		Aspiration system	$2.38*10^{-4}$ p	Modelled from Ecoinvent v 3.8 database sub process as detailed in Table S3
	Transport	Transport for large equipment	$3.96*10^{-2}$ tkm	Ecoinvent v 3.8[8]
		Transport for raw materials	8.59 kgkm	Ecoinvent v 3.8[9]
		Transport for small equipment	$1.30*10^{-1}$ kgkm	Ecoinvent v 3.8[10]
	Energy	Electric energy	28.25 kJ	Ecoinvent v 3.8[11]
			2112 Wh	Ecoinvent v 3.8[12]
			11.37 kJ	Ecoinvent v 3.8[13]
			630 Wh	Ecoinvent v 3.8[14]
			500 Wh	Ecoinvent v 3.8[15]
			2.26 kWh	Ecoinvent v 3.8[16]
Output	Air emission	ACN	$4.28*10^{-5}$ g	SimaPro airborne emission substance list[17]
		HBr	$1.23*10^{-3}$ g	SimaPro airborne emission substance list[18]
		H_2O	$1.15*10^{-6}$ g	SimaPro airborne emission substance list[19]
		Diethyl ether	$5.291*10^{-4}$ g	SimaPro airborne emission substance list[20]
	End of life	Spent solvent mixture	42.065 g	Ecoinvent v 3.8[21]
		Incineration	2.223 g	Ecoinvent v 3.8[22]

[1]The Ecoinvnet process used was: Acetonitrile {RER}| Sohio process | APOS, U.

[2]The Ecoinvent process used was: Potassium carbonate {RER}| oxidation of manganese dioxide | APOS, U.
[3]The Ecoinvent process used was: Potassium chloride {RER}| potassium chloride production | APOS, U. During the experimental work potassium iodide was used. Since it is not present in the database, potassium chloride was used as proxy.
[4]The Ecoinvent process use was: Water, deionised {Europe without Switzerland}| water production, deionised | APOS, U.
[5]The Ecoinvent process used was: Diethyl ether, without water, in 99.95% solution state {RER}| ethylene hydration | APOS, U.
[6]The Ecoinvent process used was: Magnesium sulfate {RER}| production | APOS, U.
[7]The Ecoinvent process used was: Silicone product {RER}| production | APOS, U.
[8]Transport of large equipment (aspiration system and activated air carbon filter). The Ecoinvet process used was: Transport, freight, lorry 16-32 metric ton, EURO6 {RER}| transport, freight, lorry 16-32 metric ton, EURO6 | APOS, U. An average distance of 100km was considered.
[9]Transport for reagents. The Ecoinvent process used was: Transport, freight, lorry 3.5-7.5 metric ton, EURO6 {RER}| transport, freight, lorry 3.5-7.5 metric ton, EURO6 | APOS, U. An average distance of 100km was considered.
[10]Transport for small laboratory equipment. The Ecoinvent process used was: Transport, freight, lorry 3.5-7.5 metric ton, EURO6 {RER}| transport, freight, lorry 3.5-7.5 metric ton, EURO6 | APOS, U. An average distance of 100 km was considered.
[11]Electric energy necessary for the heating up to 100°C of the reaction mixture containing ACN, Vanillin and Bromobutane. It is calculated using the expression *m*Cp*DT*, considering the specific heat in J/kg°C for each of the substances. The Ecoinvent process used was: Electricity, low voltage {IT}| electricity voltage transformation from medium to low voltage | APOS, U.
[12]Electric energy necessary to maintain the heating at 100°C of the reaction mixture for 16 hours. It is calculated using the Fourier Equation. The Ecoinvent process used was: Electricity, low voltage {IT}| electricity voltage transformation from medium to low voltage | APOS, U.
[13]Electric energy necessary to eliminate the remaining solvent (ACN) using the rotavapor. It is calculated as HvapACN*molACN, considering HvapACN as its latent heat of vaporization (29.8 kJ/mol) and molACN as the moles of the solvent. The Ecoinvent process used was: Electricity, low voltage {IT}| electricity voltage transformation from medium to low voltage | APOS, U.
[14]Electric energy necessary to use the heating stirrer during the workup phase for 1 hour. It is calculated as power*time, considering 630W as the heating stirrer power. The Ecoinvent process used was: Electricity, low voltage {IT}| electricity voltage transformation from medium to low voltage | APOS, U.
[15]Electric energy necessary to use the rotavapor after the workup phase for 1 hour. It is calculated as power*time, considering 500W as the rotavapor power. The Ecoinvent process used was: Electricity, low voltage {IT}| electricity voltage transformation from medium to low voltage | APOS, U.
[16]Electric energy necessary to the use of the aspiration system for the whole synthesis time, i.e. 19 h. The power was calculated by considering the air flow rate of 250 m3/h, a total load loss of 110.8076 kg/m2, and an efficiency of 90%: Electricity, low voltage {IT}| electricity voltage transformation from medium to low voltage | APOS, U.
[17]Amount of ACN released into the atmosphere, as calculated by the formula reported in equation 4 of the main manuscript.
[18]Amount of HBr released into the atmosphere, as calculated by the formula reported in equation 4 of the main manuscript.
[19]Amount of Water released into the atmosphere, as calculated by the formula reported in equation 4 of the main manuscript.
[20]Amount of Diethyl ether released into the atmosphere, as calculated by the formula reported in equation 4 of the main manuscript.
[21]End of life of liquid waste, including ACN, Diethyl ether and water. The Ecoinvent process used was: Spent solvent mixture {Europe without Switzerland}| treatment of spent solvent mixture, hazardous waste incineration, with energy recovery | APOS, U.
[22]End of life of the solid waste recovered after the filtration following the first part of the synthesis. The Ecoinvent process used was: Hazardous waste, for incineration {RoW}| treatment of hazardous waste, hazardous waste incineration, with energy recovery | APOS, U.

Table S40. Contributions to the Life Cycle Inventory (LCI) for the synthesis of 0.31514 g of 4-butoxy-3-methoxybenzaldehyde. This is Reaction 17 (Run 2), carried in Acetone as solvent with a reaction time of 8 hours, as reported in Table 1 in the main manuscript.

Description			Amount	Process Data Source
Input	Materials	Acetone	15.68 g	Ecoinvent v 3.8[1]
		K_2CO_3	0.458 g	Ecoinvent v 3.8[2]
		KCl	1.123 g	Ecoinvent v 3.8[3]
		Bromobutane	0.947 g	Modelled from Ecoinvent v 3.8 database sub process as detailed in Table S23
		Vanillin	0.512 g	Modelled from Ecoinvent v 3.8 database sub process as detailed in Table S20
		H_2O	5 g	Ecoinvent v 3.8[4]
		Diethyl ether	21.405 g	Ecoinvent v 3.8[5]
		Mg_2SO_4	1 g	Ecoinvent v 3.8[6]
		Silicon oil	$1.72*10^{-2}$ p	Ecoinvent v 3.8[7]
	Equipment/ plants	Magnetic stirrer/heater	$1.8*10^{-4}$ p	Modelled from Ecoinvent v 3.8 database sub process as detailed in Table S18
		Round bottom flask	$3.47*10^{-4}$ p	Modelled from Ecoinvent v 3.8 database sub process as detailed in Table S15
		Separatory funnel	$2.33*10^{-5}$ p	Modelled from Ecoinvent v 3.8 database sub process as detailed in Table S9
		Reflux condenser	$3.12*10^{-4}$ p	Modelled from Ecoinvent v 3.8 database sub process as detailed in Table S13
		Silicon oil container	$3.47*10^{-4}$ p	Modelled from Ecoinvent v 3.8 database sub process as detailed in Table S6
		Funnel	$2.90*10^{-6}$ p	Modelled from Ecoinvent v 3.8 database sub process as detailed in Table S8
		Filter	$3.18*10^{-6}$ p	Modelled from Ecoinvent v 3.8 database

				sub process as detailed in Table S7
		Fitting reflux condenser	$2.78*10^{-4}$ p	Modelled from Ecoinvent v 3.8 database sub process as detailed in Table S12
		Rubber tube	$5.56*10^{-3}$ p	Modelled from Ecoinvent v 3.8 database sub process as detailed in Table S14
		Activated carbon air filter	$5.5*10^{-4}$ p	Modelled from Ecoinvent v 3.8 database sub process as detailed in Table S4
		Aspiration system	$1.38*10^{-4}$ p	Modelled from Ecoinvent v 3.8 database sub process as detailed in Table S3
	Transport	Transport for large equipment	$2.29*10^{-2}$ tkm	Ecoinvent v 3.8[8]
		Transport for raw materials	8.72 kgkm	Ecoinvent v 3.8[9]
		Transport for small equipment	$6.88*10^{-2}$ kgkm	Ecoinvent v 3.8[10]
	Energy	Electric energy	11.40 kJ	Ecoinvent v 3.8[11]
			480 Wh	Ecoinvent v 3.8[12]
			8.64 kJ	Ecoinvent v 3.8[13]
			630 Wh	Ecoinvent v 3.8[14]
			500 Wh	Ecoinvent v 3.8[15]
			0.92 kWh	Ecoinvent v 3.8[16]
Output	Air emission	Acetone	$1.44*10^{-4}$ g	SimaPro airborne emission substance list[17]
		HBr	$6.62*10^{-3}$ g	SimaPro airborne emission substance list[18]
		H_2O	$1.15*10^{-6}$ g	SimaPro airborne emission substance list[19]
		Diethyl ether	$5.29*10^{-4}$ g	SimaPro airborne emission substance list[20]
	End of life	Spent solvent mixture	42.085 g	Ecoinvent v 3.8[21]
		Incineration	2.581 g	Ecoinvent v 3.8[22]

[1]The Ecoinvnet process used was: Acetone, liquid {RER}| production | APOS, U

[2]The Ecoinvent process used was: Potassium carbonate {RER}| oxidation of manganese dioxide | APOS, U.
[3]The Ecoinvent process used was: Potassium chloride {RER}| potassium chloride production | APOS, U. During the experimental work potassium iodide was used. Since it is not present in the database, potassium chloride was used as proxy.
[4]The Ecoinvent process use was: Water, deionised {Europe without Switzerland}| water production, deionised | APOS, U.
[5]The Ecoinvent process used was: Diethyl ether, without water, in 99.95% solution state {RER}| ethylene hydration | APOS, U.
[6]The Ecoinvent process used was: Magnesium sulfate {RER}| production | APOS, U.
[7]The Ecoinvent process used was: Silicone product {RER}| production | APOS, U.
[8]Transport of large equipment (aspiration system and activated air carbon filter). The Ecoinvet process used was: Transport, freight, lorry 16-32 metric ton, EURO6 {RER}| transport, freight, lorry 16-32 metric ton, EURO6 | APOS, U. An average distance of 100km was considered.
[9]Transport for reagents. The Ecoinvent process used was: Transport, freight, lorry 3.5-7.5 metric ton, EURO6 {RER}| transport, freight, lorry 3.5-7.5 metric ton, EURO6 | APOS, U. An average distance of 100km was considered.
[10]Transport for small laboratory equipment. The Ecoinvent process used was: Transport, freight, lorry 3.5-7.5 metric ton, EURO6 {RER}| transport, freight, lorry 3.5-7.5 metric ton, EURO6 | APOS, U. An average distance of 100 km was considered.
[11]Electric energy necessary for the heating up to 100°C of the reaction mixture containing Acetone, Vanillin and Bromobutane. It is calculated using the expression *m*Cp*DT*, considering the specific heat in J/kg°C for each of the substances. The Ecoinvent process used was: Electricity, low voltage {IT}| electricity voltage transformation from medium to low voltage | APOS, U.
[12]Electric energy necessary to maintain the heating at 100°C of the reaction mixture for 8 hours. It is calculated using the Fourier Equation. The Ecoinvent process used was: Electricity, low voltage {IT}| electricity voltage transformation from medium to low voltage | APOS, U.
[13]Electric energy necessary to eliminate the remaining solvent (ACN) using the rotavapor. It is calculated as HvapAcetone*molAcetone, considering HvapAcentone as its latent heat of vaporization (32 kJ/mol) and molAcetone as the moles of the solvent. The Ecoinvent process used was: Electricity, low voltage {IT}| electricity voltage transformation from medium to low voltage | APOS, U.
[14]Electric energy necessary to use the heating stirrer during the workup phase for 1 hour. It is calculated as power*time, considering 630W as the heating stirrer power. The Ecoinvent process used was: Electricity, low voltage {IT}| electricity voltage transformation from medium to low voltage | APOS, U.
[15]Electric energy necessary to use the rotavapor after the workup phase for 1 hour. It is calculated as power*time, considering 500W as the rotavapor power. The Ecoinvent process used was: Electricity, low voltage {IT}| electricity voltage transformation from medium to low voltage | APOS, U.
[16]Electric energy necessary to the use of the aspiration system for the whole synthesis time, i.e. 11 h. The power was calculated by considering the air flow rate of 250 m3/h, a total load loss of 110.8076 kg/m2, and an efficiency of 90%: Electricity, low voltage {IT}| electricity voltage transformation from medium to low voltage | APOS, U.
[17]Amount of Acetone released into the atmosphere, as calculated by the formula reported in equation 4 of the main manuscript.
[18]Amount of HBr released into the atmosphere, as calculated by the formula reported in equation 4 of the main manuscript.
[19]Amount of Water released into the atmosphere, as calculated by the formula reported in equation 4 of the main manuscript.
[20]Amount of Diethyl ether released into the atmosphere, as calculated by the formula reported in equation 4 of the main manuscript.
[21]End of life of liquid waste, including Acetone, Diethyl ether and water. The Ecoinvent process used was: Spent solvent mixture {Europe without Switzerland}| treatment of spent solvent mixture, hazardous waste incineration, with energy recovery | APOS, U.
[22]End of life of the solid waste recovered after the filtration following the first part of the synthesis. The Ecoinvent process used was: Hazardous waste, for incineration {RoW}| treatment of hazardous waste, hazardous waste incineration, with energy recovery | APOS, U.

Table S41. Contributions to the Life Cycle Inventory (LCI) for the synthesis of 0.084 g of 4-butoxy-3-methoxybenzaldehyde. This is Reaction 18 (Run 6), carried in Acetonitrile as solvent with a reaction time of 8 hours, as reported in Table 1 in the main manuscript.

Description			Amount	Process Data Source
Input	Materials	ACN	15.66 g	Ecoinvent v 3.8[1]
		K_2CO_3	0.451 g	Ecoinvent v 3.8[2]
		KCl	0 g	Ecoinvent v 3.8[3]
		Bromobutane	0.465 g	Modelled from Ecoinvent v 3.8 database sub process as detailed in Table S23
		Vanillin	0.503 g	Modelled from Ecoinvent v 3.8 database sub process as detailed in Table S20
		H_2O	5 g	Ecoinvent v 3.8[4]
		Diethyl ether	21.405 g	Ecoinvent v 3.8[5]
		Mg_2SO_4	1 g	Ecoinvent v 3.8[6]
		Silicon oil	$1.72*10^{-2}$ p	Ecoinvent v 3.8[7]
	Equipment/ plants	Magnetic stirrer/heater	$1.8*10^{-4}$ p	Modelled from Ecoinvent v 3.8 database sub process as detailed in Table S18
		Round bottom flask	$3.47*10^{-4}$ p	Modelled from Ecoinvent v 3.8 database sub process as detailed in Table S15
		Separatory funnel	$2.33*10^{-5}$ p	Modelled from Ecoinvent v 3.8 database sub process as detailed in Table S9
		Reflux condenser	$3.12*10^{-4}$ p	Modelled from Ecoinvent v 3.8 database sub process as detailed in Table S13
		Silicon oil container	$3.47*10^{-4}$ p	Modelled from Ecoinvent v 3.8 database sub process as detailed in Table S6
		Funnel	$2.90*10^{-6}$ p	Modelled from Ecoinvent v 3.8 database sub process as detailed in Table S8
		Filter	$3.18*10^{-6}$ p	Modelled from Ecoinvent v 3.8 database

				sub process as detailed in Table S7
		Fitting reflux condenser	$2.78*10^{-4}$ p	Modelled from Ecoinvent v 3.8 database sub process as detailed in Table S12
		Rubber tube	$5.56*10^{-4}$ p	Modelled from Ecoinvent v 3.8 database sub process as detailed in Table S14
		Activated carbon air filter	$5.5*10^{-4}$ p	Modelled from Ecoinvent v 3.8 database sub process as detailed in Table S4
		Aspiration system	$1.38*10^{-4}$ p	Modelled from Ecoinvent v 3.8 database sub process as detailed in Table S3
	Transport	Transport for large equipment	$2.29*10^{-2}$ tkm	Ecoinvent v 3.8[8]
		Transport for raw materials	8.41 kgkm	Ecoinvent v 3.8[9]
		Transport for small equipment	$5.36*10^{-1}$ kgkm	Ecoinvent v 3.8[10]
	Energy	Electric energy	28.25 kJ	Ecoinvent v 3.8[11]
			1056 Wh	Ecoinvent v 3.8[12]
			11.37 kJ	Ecoinvent v 3.8[13]
			630 Wh	Ecoinvent v 3.8[14]
			500 Wh	Ecoinvent v 3.8[15]
			0.92 kWh	Ecoinvent v 3.8[16]
Output	Air emission	ACN	$4.28*10^{-5}$ g	SimaPro airborne emission substance list[17]
		HBr	$9.64*10^{-4}$ g	SimaPro airborne emission substance list[18]
		H_2O	$1.15*10^{-6}$ g	SimaPro airborne emission substance list[19]
		Diethyl ether	$5.291*10^{-4}$ g	SimaPro airborne emission substance list[20]
	End of life	Spent solvent mixture	42.065 g	Ecoinvent v 3.8[21]
		Incineration	1.451 g	Ecoinvent v 3.8[22]

[1]The Ecoinvnet process used was: Acetonitrile {RER}| Sohio process | APOS, U.

[2]The Ecoinvent process used was: Potassium carbonate {RER}| oxidation of manganese dioxide | APOS, U.
[3]The Ecoinvent process used was: Potassium chloride {RER}| potassium chloride production | APOS, U. During the experimental work potassium iodide was used. Since it is not present in the database, potassium chloride was used as proxy.
[4]The Ecoinvent process use was: Water, deionised {Europe without Switzerland}| water production, deionised | APOS, U.
[5]The Ecoinvent process used was: Diethyl ether, without water, in 99.95% solution state {RER}| ethylene hydration | APOS, U.
[6]The Ecoinvent process used was: Magnesium sulfate {RER}| production | APOS, U.
[7]The Ecoinvent process used was: Silicone product {RER}| production | APOS, U.
[8]Transport of large equipment (aspiration system and activated air carbon filter). The Ecoinvet process used was: Transport, freight, lorry 16-32 metric ton, EURO6 {RER}| transport, freight, lorry 16-32 metric ton, EURO6 | APOS, U. An average distance of 100km was considered.
[9]Transport for reagents. The Ecoinvent process used was: Transport, freight, lorry 3.5-7.5 metric ton, EURO6 {RER}| transport, freight, lorry 3.5-7.5 metric ton, EURO6 | APOS, U. An average distance of 100km was considered.
[10]Transport for small laboratory equipment. The Ecoinvent process used was: Transport, freight, lorry 3.5-7.5 metric ton, EURO6 {RER}| transport, freight, lorry 3.5-7.5 metric ton, EURO6 | APOS, U. An average distance of 100 km was considered.
[11]Electric energy necessary for the heating up to 100°C of the reaction mixture containing ACN, Vanillin and Bromobutane. It is calculated using the expression *m*Cp*DT*, considering the specific heat in J/kg°C for each of the substances. The Ecoinvent process used was: Electricity, low voltage {IT}| electricity voltage transformation from medium to low voltage | APOS, U.
[12]Electric energy necessary to maintain the heating at 100°C of the reaction mixture for 8 hours. It is calculated using the Fourier Equation. The Ecoinvent process used was: Electricity, low voltage {IT}| electricity voltage transformation from medium to low voltage | APOS, U.
[13]Electric energy necessary to eliminate the remaining solvent (ACN) using the rotavapor. It is calculated as HvapACN*molACN, considering HvapACN as its latent heat of vaporization (29.8 kJ/mol) and molACN as the moles of the solvent. The Ecoinvent process used was: Electricity, low voltage {IT}| electricity voltage transformation from medium to low voltage | APOS, U.
[14]Electric energy necessary to use the heating stirrer during the workup phase for 1 hour. It is calculated as power*time, considering 630W as the heating stirrer power. The Ecoinvent process used was: Electricity, low voltage {IT}| electricity voltage transformation from medium to low voltage | APOS, U.
[15]Electric energy necessary to use the rotavapor after the workup phase for 1 hour. It is calculated as power*time, considering 500W as the rotavapor power. The Ecoinvent process used was: Electricity, low voltage {IT}| electricity voltage transformation from medium to low voltage | APOS, U.
[16]Electric energy necessary to the use of the aspiration system for the whole synthesis time, i.e. 11 h. The power was calculated by considering the air flow rate of 250 m3/h, a total load loss of 110.8076 kg/m2, and an efficiency of 90%: Electricity, low voltage {IT}| electricity voltage transformation from medium to low voltage | APOS, U.
[17]Amount of ACN released into the atmosphere, as calculated by the formula reported in equation 4 of the main manuscript.
[18]Amount of HBr released into the atmosphere, as calculated by the formula reported in equation 4 of the main manuscript.
[19]Amount of Water released into the atmosphere, as calculated by the formula reported in equation 4 of the main manuscript.
[20]Amount of Diethyl ether released into the atmosphere, as calculated by the formula reported in equation 4 of the main manuscript.
[21]End of life of liquid waste, including ACN, Diethyl ether and water. The Ecoinvent process used was: Spent solvent mixture {Europe without Switzerland}| treatment of spent solvent mixture, hazardous waste incineration, with energy recovery | APOS, U.
[22]End of life of the solid waste recovered after the filtration following the first part of the synthesis. The Ecoinvent process used was: Hazardous waste, for incineration {RoW}| treatment of hazardous waste, hazardous waste incineration, with energy recovery | APOS, U.

Table S42. Contributions to the Life Cycle Inventory (LCI) for the synthesis of 0.4324 g of 4-butoxy-3-methoxybenzaldehyde. This is Reaction 19 (Run 10), carried in DMF as solvent with a reaction time of 8 hours, as reported in Table 1 in the main manuscript.

Description			Amount	Process Data Source
Input	Materials	DMF	4.72 g	Ecoinvent v 3.8[1]
		K_2CO_3	0.917 g	Ecoinvent v 3.8[2]
		KCl	1.065 g	Ecoinvent v 3.8[3]
		Bromobutane	0.927 g	Modelled from Ecoinvent v 3.8 database sub process as detailed in Table S23
		Vanillin	0.528 g	Modelled from Ecoinvent v 3.8 database sub process as detailed in Table S20
		H_2O	5 g	Ecoinvent v 3.8[4]
		Diethyl ether	21.405 g	Ecoinvent v 3.8[5]
		Mg_2SO_4	1 g	Ecoinvent v 3.8[6]
		Silicon oil	$1.72*10^{-2}$ p	Ecoinvent v 3.8[7]
	Equipment/ plants	Magnetic stirrer/heater	$1.8*10^{-4}$ p	Modelled from Ecoinvent v 3.8 database sub process as detailed in Table S18
		Round bottom flask	$3.47*10^{-4}$ p	Modelled from Ecoinvent v 3.8 database sub process as detailed in Table S15
		Separatory funnel	$2.33*10^{-5}$ p	Modelled from Ecoinvent v 3.8 database sub process as detailed in Table S9
		Reflux condenser	$3.12*10^{-4}$ p	Modelled from Ecoinvent v 3.8 database sub process as detailed in Table S13
		Silicon oil container	$3.47*10^{-4}$ p	Modelled from Ecoinvent v 3.8 database sub process as detailed in Table S6
		Funnel	$2.90*10^{-6}$ p	Modelled from Ecoinvent v 3.8 database sub process as detailed in Table S8
		Filter	$3.18*10^{-6}$ p	Modelled from Ecoinvent v 3.8 database

				sub process as detailed in Table S7
		Fitting reflux condenser	3.12*10^{-4} p	Modelled from Ecoinvent v 3.8 database sub process as detailed in Table S12
		Rubber tube	5.56*10^{-4} p	Modelled from Ecoinvent v 3.8 database sub process as detailed in Table S14
		Activated carbon air filter	5.5*10^{-4} p	Modelled from Ecoinvent v 3.8 database sub process as detailed in Table S4
		Aspiration system	1.38*10^{-4} p	Modelled from Ecoinvent v 3.8 database sub process as detailed in Table S3
	Transport	Transport for large equipment	2.29*10^{-2} tkm	Ecoinvent v 3.8[8]
		Transport for raw materials	6.72 kgkm	Ecoinvent v 3.8[9]
		Transport for small equipment	6.89*10^{-2} kgkm	Ecoinvent v 3.8[10]
	Energy	Electric energy	42.90 kJ	Ecoinvent v 3.8[11]
			1440 Wh	Ecoinvent v 3.8[12]
			3.015 kJ	Ecoinvent v 3.8[13]
			630 Wh	Ecoinvent v 3.8[14]
			500 Wh	Ecoinvent v 3.8[15]
			0.92 kWh	Ecoinvent v 3.8[16]
Output	Air emission	DMF	7.28*10^{-7} g	SimaPro airborne emission substance list[17]
		HBr	4.41*10^{-3} g	SimaPro airborne emission substance list[18]
		H_2O	1.15*10^{-6} g	SimaPro airborne emission substance list[19]
		Diethyl ether	5.291*10^{-4} g	SimaPro airborne emission substance list[20]
	End of life	Spent solvent mixture	31.125 g	Ecoinvent v 3.8[21]
		Incineration	2.982 g	Ecoinvent v 3.8[22]

[1]The Ecoinvnet process used was: N,N-dimethylformamide {RER}∣ production ∣ APOS, U.

[2]The Ecoinvent process used was: Potassium carbonate {RER}| oxidation of manganese dioxide | APOS, U.
[3]The Ecoinvent process used was: Potassium chloride {RER}| potassium chloride production | APOS, U. During the experimental work potassium iodide was used. Since it is not present in the database, potassium chloride was used as proxy.
[4]The Ecoinvent process use was: Water, deionised {Europe without Switzerland}| water production, deionised | APOS, U.
[5]The Ecoinvent process used was: Diethyl ether, without water, in 99.95% solution state {RER}| ethylene hydration | APOS, U.
[6]The Ecoinvent process used was: Magnesium sulfate {RER}| production | APOS, U.
[7]The Ecoinvent process used was: Silicone product {RER}| production | APOS, U.
[8]Transport of large equipment (aspiration system and activated air carbon filter). The Ecoinvet process used was: Transport, freight, lorry 16-32 metric ton, EURO6 {RER}| transport, freight, lorry 16-32 metric ton, EURO6 | APOS, U. An average distance of 100km was considered.
[9]Transport for reagents. The Ecoinvent process used was: Transport, freight, lorry 3.5-7.5 metric ton, EURO6 {RER}| transport, freight, lorry 3.5-7.5 metric ton, EURO6 | APOS, U. An average distance of 100km was considered.
[10]Transport for small laboratory equipment. The Ecoinvent process used was: Transport, freight, lorry 3.5-7.5 metric ton, EURO6 {RER}| transport, freight, lorry 3.5-7.5 metric ton, EURO6 | APOS, U. An average distance of 100 km was considered.
[11]Electric energy necessary for the heating up to 100°C of the reaction mixture containing DMF, Vanillin and Bromobutane. It is calculated using the expression *m*Cp*DT*, considering the specific heat in J/kg°C for each of the substances. The Ecoinvent process used was: Electricity, low voltage {IT}| electricity voltage transformation from medium to low voltage | APOS, U.
[12]Electric energy necessary to maintain the heating at 100°C of the reaction mixture for 8 hours. It is calculated using the Fourier Equation. The Ecoinvent process used was: Electricity, low voltage {IT}| electricity voltage transformation from medium to low voltage | APOS, U.
[13]Electric energy necessary to eliminate the remaining solvent (DMF) using the rotavapor. It is calculated as HvapDMF*molDMF, considering HvapDMF as its latent heat of vaporization (46.7 kJ/mol) and molDMF as the moles of the solvent. The Ecoinvent process used was: Electricity, low voltage {IT}| electricity voltage transformation from medium to low voltage | APOS, U.
[14]Electric energy necessary to use the heating stirrer during the workup phase for 1 hour. It is calculated as power*time, considering 630W as the heating stirrer power. The Ecoinvent process used was: Electricity, low voltage {IT}| electricity voltage transformation from medium to low voltage | APOS, U.
[15]Electric energy necessary to use the rotavapor after the workup phase for 1 hour. It is calculated as power*time, considering 500W as the rotavapor power. The Ecoinvent process used was: Electricity, low voltage {IT}| electricity voltage transformation from medium to low voltage | APOS, U.
[16]Electric energy necessary to the use of the aspiration system for the whole synthesis time, i.e. 11 h. The power was calculated by considering the air flow rate of 250 m3/h, a total load loss of 110.8076 kg/m2, and an efficiency of 90%: Electricity, low voltage {IT}| electricity voltage transformation from medium to low voltage | APOS, U.
[17]Amount of DMF released into the atmosphere, as calculated by the formula reported in equation 4 of the main manuscript.
[18]Amount of HBr released into the atmosphere, as calculated by the formula reported in equation 4 of the main manuscript.
[19]Amount of Water released into the atmosphere, as calculated by the formula reported in equation 4 of the main manuscript.
[20]Amount of Diethyl ether released into the atmosphere, as calculated by the formula reported in equation 4 of the main manuscript.
[21]End of life of liquid waste, including DMF, Diethyl ether and water. The Ecoinvent process used was: Spent solvent mixture {Europe without Switzerland}| treatment of spent solvent mixture, hazardous waste incineration, with energy recovery | APOS, U.
[22]End of life of the solid waste recovered after the filtration following the first part of the synthesis. The Ecoinvent process used was: Hazardous waste, for incineration {RoW}| treatment of hazardous waste, hazardous waste incineration, with energy recovery | APOS, U.

References

1. Douglass F. Taber, Shweta Patel, Travis M. Hambleton and Emma E. Winkel. Vanillin Synthesis from 4-Hydroxybenzaldehyde. *Journal of Chemical Education*, **2007**, v84 n7 p1158. http://dx.doi.org/10.1021/ed084p1158

2. Yoffe, D., Frim, R., Ukeles, S.D., Dagani, M. J., Barda, H.J., Benya, T.J., Sanders, D.C. Bromine Compounds. Bromine Componuds. *Ullmann's Encyclopedia of Industrial Chemistry*, 7th edition, **2013,** 1-31, doi: 10.1002/14356007.a04_405.pub210.1002/14356007.a04_405.pub2.

3. Williamson, K.L., Minard, R.D., Masters, K.M. *Macroscale and Microscale Organic Experiments*, 5th edition. Houghton Mifflin Company: Boston, **2007**.
